# Organ-on-chip platforms for nanoparticle toxicity and efficacy assessment: Advancing beyond traditional in vitro and in vivo models

**DOI:** 10.1016/j.mtbio.2025.102053

**Published:** 2025-07-04

**Authors:** Ana Regina Sampaio, Renata Faria Maia, Maria Camilla Ciardulli, Hélder A. Santos, Bruno Sarmento

**Affiliations:** a i3S – Instituto de Investigação e Inovação em Saúde, Universidade do Porto, Rua Alfredo Allen 208, Porto, 4200-135, Portugal; b Department of Biomaterials and Biomedical Technology, The Personalized Medicine Research Institute (PRECISION), The University Medical Center Groningen (UMCG), University of Groningen, the Netherlands; c INEB – Instituto de Engenharia Biomédica, Universidade do Porto, Rua Alfredo Allen 208, Porto, 4200-135, Portugal; d IUCS – Instituto Universitário de Ciências da Saúde, CESPU, Rua Central de Gandra 1317, Gandra, 4585-116, Portugal

**Keywords:** Organ-on-Chip, Microfluidics, Nanoparticles, Personalized medicine, Translational research

## Abstract

Nanoparticles (NPs) have unique properties, such as their high surface-to-volume ratio, nanoscale size, and functionalization potential, make them promising for a variety of biomedical applications, including drug delivery, diagnostics, and targeted therapy. With rapid progress in NPs research, increasing efforts are being made to develop new technologies for *in vitro* modeling and analysis of the efficacy and safety of nanotherapeutics in human physiological systems. Organ-on-chip (OoC) platforms are a cutting-edge alternative to traditional *in vitro* and *in vivo* models that provide a ground-breaking way to evaluate the toxicity and therapeutic efficacy of NPs. This review explores the advancements and achievements in OoC technology, focusing on explore the recent advances in healthy and diseases models and its potential to enhance NPs safety and efficacy. By simulating human biological systems, OoC help identify negative effects and mechanisms of action, reducing preclinical research failures and promoting targeted treatments. These platforms can cut down on drug development time and costs, making them invaluable for personalized medicine. However, challenges such as scalability and regulatory hurdles must be addressed before OoC can become a standard in NPs research and drug development. This review offers a critical and current summary of recent developments in OoC models for nanoformulation screening, emphasizing how they affect preclinical results and how they might be incorporated into the development of nanomedicine. It seeks to assist researchers and professionals in the industry in making well-informed choices regarding the use of these platforms.

## Graphical abstract

**Figure:**
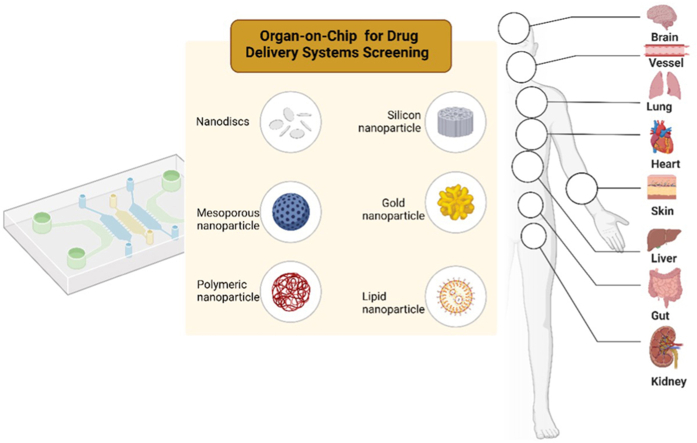

## 1 Introduction

The translation from laboratory research to clinical application remains a major challenge, particularly in the field of nanomedicine. Conventional preclinical evaluation tools, such as two-dimensional (2D) cell cultures and animal models, have significant limitations in predicting human responses. In 2D cultures, cells grow as flat monolayers, leading to altered morphology, impaired cell–extracellular matrix (ECM) interactions, and disrupted signaling. These systems fail to replicate the complex three-dimensional (3D) architecture, ECM interactions, and dynamic biochemical gradients present *in vivo*. In addition, the lack of tissue-level organization and physiological flow, results in altered nanoparticle uptake, diffusion, and cellular responses that do not reflect human physiological conditions. Moreover, they fail to capture key factors such as oxygen gradients, mechanical stress, and intercellular signaling that strongly influence NP behavior and toxicity profiles [[1], [2], [3], [4], [5]]. Animal models, while useful for studying systemic responses, often fail to accurately recapitulate human physiology due to interspecies differences in immune responses, metabolism, and organ function. These differences contribute to the high attrition rate of drugs that perform well in preclinical trials but show limited efficacy in humans [3,6]. These limitations are especially critical when assessing nanoparticle (NP)-based formulations, whose efficacy, toxicity, biodistribution, clearance, and immunogenicity are highly dependent on interactions with human-specific microenvironments [7].

In this context, organ-on-chip (OoC) technology has emerged as a promising alternative, combining controlled microenvironments, living human cells, and microfluidic engineering to mimic the structural, mechanical, and functional characteristics of tissues and organs [3,4,6]. Compared to static models, these platforms provide more physiologically and pathologically relevant environments by dynamically simulating biomechanical forces, such as oxygen gradients, shear stress, and rhythmic contraction [1,2,5]. 3D cell cultures, hydrogels, and co-culture systems enhances tissue microenvironment reconstruction and supports the assessment of relevant cellular phenotypes [8,9]. Recent technological advances have expanded the capabilities of OoC systems. The integration of biosensors and nanosensors enables real-time monitoring of physiological parameters such as pH, oxygen levels, metabolic activity, and toxicity biomarkers, allowing continuous, non-invasive functional analysis [10]. By precisely replicating the tissue architectures and microvascular networks of native organs, advanced techniques such as 3D bioprinting and microfabrication have significantly enhanced the relevance and applicability of experimental data [8,9]. Moreover, the development of complex co-cultures and interconnected multi-organ chips has opened avenues for studying systemic interactions, such as liver–intestine or liver–kidney crosstalk, which are crucial for understanding the metabolism and toxicity of NPs on a whole-body level [11].

However, important technological trade-offs remain. There is a constant tension between physiological fidelity, achieved through highly complex and tailored models, and the scalability and reproducibility required for high-throughput screening and industrial standardization [12]. Complex models, while biologically accurate, tend to be costly and technically demanding, limiting their scalability. Simpler systems, on the other hand, might be more scalable but miss important biological characteristics. In addition, the broad use of OoC systems in the pharmaceutical industry and regulatory bodies is also still hampered by issues with regulatory acceptance, inter-laboratory validation, and integration into pharmaceutical development pipelines [10,13].

Parallel to these developments, NPs-based therapies have demonstrated significant promise in improving drug stability, bioavailability, and controlled release. These include lipid-based, metallic, polymeric, and hybrid NPs [14,15]. Surface functionalization allows for targeted delivery to specific tissues, promoting selective accumulation and reducing off-target effects [7,15]. However, reliable and human-relevant models are necessary for an accurate assessment of their safety and efficacy, and OoC platforms are becoming more capable of meeting this need. The application of OoC systems in particular nanomedicine contexts has been the subject of multiple reviews in recent years. For instance, Chen et al. [13] reviewed recent advancements in 3D tissue engineering and organ-on-a-chip systems, highlighting materials, fabrication methods, and monitoring technologies; Stavrou et al. [11] examined the use of OoC systems in nanomedicine, emphasizing their role in studying NPs interactions, toxicity, and biological barrier modeling; Singh et al. [16] analyzed recent developments in OoC technology, discussing fabrication methods, disease modeling applications, and the potential of these systems; and Rodrigues et al. [12] discussed recent advances in organ-on-a-chip platforms for preclinical validation of nanomaterials, highlighting their potential to bridge the gap between preclinical and clinical outcomes in cancer nanomedicine. Although useful, these reviews frequently focus on isolated organ models or highlight toxicological or technological features without providing a thorough comparison of healthy and diseased organ models or their use in screening nanoformulations in clinically relevant settings.

This review aims are exploring recent developments in OoC models for nanoformulation screening by contrasting platforms that represent healthy and diseased tissues and emphasizing how these differences affect preclinical results. It also aims to clarify important research trends, discuss technological trade-offs, and identify challenges and opportunities for integrating these platforms into NP-based drug discovery and development pipelines. This review aims to address a significant gap in the literature by providing a critical and current viewpoint, assisting researchers and industry professionals in making more informed decisions regarding the adoption of OoC platforms for nanomedicine applications.

## 2 OoC technologies

The field of OoC receive great attention because of the potential to better understand drug responses and therapeutic effects *in vitro*. OoC allow the simulation of biomechanical forces present *in vivo*, such as blood flow and mechanical stress. For example, shear stress can influence the uptake and metabolism of NPs [17]. Among the most widely developed OoC platforms are those mimicking the liver, lung, heart, kidney, intestine, and blood–brain barrier (BBB). Each model captures key physiological features: the lung-on-chip (LUoC) recreates the dynamic air–blood interface and simulates respiratory mechanics through cyclic stretching; the liver-on-chip (LoC) replicates hepatic zonation and metabolic gradients [11]; and the heart-on-chip (HoC) enables real-time monitoring of contractility and electrophysiological responses, making it particularly valuable for cardiotoxicity screening [16].

Vascularization is essential for regulating the distribution and removal of NPs from the body. To increase the safety and effectiveness of NPs in biological applications, it is essential to comprehend these interactions. The uses of OoC enhances the predictive power of preclinical studies, as describe by Lin et al. which demonstrated that physiological shear stress (PSS) significantly enhances glycocalyx integrity and barrier function, effectively replicate healthy vascular responses. Furthermore, the incorporation of sevoflurane was shown to restore glycocalyx integrity after exposure to low shear stress (LSS), further supporting the relevance of the model in studying vascular health and potential therapeutic interventions [18].

The integration of multiple organ systems—so-called multi-organ or body-on-a-chip platforms—further expands the utility of OoC by enabling the study of inter-organ communication and systemic pharmacokinetics. These interconnected systems are particularly valuable for investigating metabolic toxicity and drug metabolism, offering a more holistic view of compound behavior in the human body [11,12]. Huang et al. describes a liver-kidney-on-chip system (LKOCBCS) for drug toxicity assessment [19]. The LKOCBCS demonstrated superior sensitivity in detecting cyclosporin A (CsA)-induced toxicity at lower concentrations compared to traditional static monocellular models. This enhanced sensitivity is particularly important given that CsA toxicity in animal models often fails to replicate human-specific responses due to interspecies differences in metabolism and immune function. A key advantage of the LKOCBCS is its ability to isolate and analyze the organ-specific effects of drug metabolites, such as the impact of hepatic metabolism on renal tubules—an interaction that is difficult to dissect in whole-animal systems. In this model, repeated CsA exposure led to both hepatotoxicity and nephrotoxicity, reflecting clinical observations. Biomarker analysis revealed significant increases in alanine aminotransferase (ALT), aspartate aminotransferase (AST), and kidney injury molecule-1 (KIM-1), underscoring the importance of inter-organ communication in toxicity mechanisms [19].

Another advancement is the incorporation of induced pluripotent stem cells (iPSCs), which allows for the generation of patient-specific OoC models. This approach supports the development of personalized medicine platforms, enabling the study of rare or genetically driven diseases in a patient-relevant context and advancing the goals of precision medicine [10,13,16]. In the following section—Table 1—we provide an overview of various OoC platforms and their evolving role in replicating human physiology with increasing precision. Recent innovations have expanded the capabilities of these systems, enabling more fidelity modeling of both healthy tissue function and disease-specific microenvironments.

**Table 1:** OoC models, fabrication techniques and readouts.

Cell types	Type of model	Fabrication technique or device	Readouts	Ref.
Vascularization-on-chip				
human umbilical vein EC (HUVEC), Primary human intestinal fibroblasts	Healthy model	3D-printed polylactic acid (PLA) core, which supports the hydrogel (fibronectin-collagen) and embedded channels	Immunohistochemistry, Gene expression	[27]
Renal proximal tubular epithelial cells (RPTEC), HUVEC	Healthy model	pre-wrapping seeding technique combined with 3D sugar printing technology	Shear stress (low flow conditions), Protein expression, Cell viability, and Barrier function assay	[28]
Human induced Pluripotent Stem Cell (hiPSC) and stem cell-derived EC	Healthy model and atherosclerosis model	3D stereolithography printing to create a chip that holds six identical unit cells, each containing a hydrogel chamber and two fluid reservoirs. After filling the hydrogel chamber with a collagen solution and removing a nylon filament to form a microchannel	Shear stress (flow conditions), Transcriptomic and Functional analysis	[29]
HUVEC (extra organ: Pancreatic Isle)	Healthy model	3D printing and PDMS chip: Viscous finger patterning technique	Fluorescent staining	[30]
hiPSC, human brain vascular pericytes, and hiPSC-derived hAs (hAs)	Healthy model	Commercially available AIM biotech identx9; 3D culture chip	Morphology, Protein expression, Barrier function assay	[31]
Adipose stem cells, HUVEC	Healthy model	Soft litography to create five layers: (1) a bottom glass layer, (2) a hydrogel guide layer, (3) a microfluidic channel layer, (4) a top glass layer and (5) a medium reservoir layer	Morphology, Cytochemical staining, Flow conditions	[32]
All blood cells	Diseases model	soft lithography to create PDMS microfluidic channels	Thrombus formation, Thrombus characteristics, such as porosity	[33]
All blood cells	Diseases model	soft lithography to create PDMS microfluidic channels, which were then bonded to unplasticized polyvinyl chloride (PVC) and polycarbonate (PC) sheets	Platelet adhesion and activation	[34]
HUVEC	Diseases model (hypoxia)	soft lithography to create PDMS microfluidic channels,	Collective cell migration, expression of intercellular adhesion molecule-1 (ICAM-1) and the area of VE-cadherin were evaluated to understand the functional differences in the EC monolayer under various conditions of hypoxia and flow	[35]
HUVEC	Diseases model (hypoxia)	soft lithography to create PDMS layers (a top layer with five individual channels, a middle layer containing a thin membrane, and a bottom layer featuring a main channel that splits into the five branch channels. The channels on the top layer were filled with UV-curable resin, which was injected at a specific pressure to induce permanent deformation of the membrane	Effects of Shear Stress Morphology and Adhesiveness	[36]
Human coronary artery EC (HCAEC)	Diseases model	soft lithography to create PDMS microfluidic channels	Morphological changes and expression levels of proteins	[37]
ECs and smooth muscle cells (SMC)	Heatly Model	soft lithography techniques three glass layers that are stacked and compressed using a connection clamp and a porous membrane (commercial polyethylene terephthalate (PET))	Flow-Mediated Transcriptomic Changes and Drug Testing	[38]
Human aortic smooth muscle cells (HASMC) and HUVEC	Heatly Model	soft lithography to create PDMS microfluidic channels,	Permeability Assays, Impact of Shear Stress and Drug Testing	[18]
HUVEC	Diseases Model	Organoready Blood Vessel HUVEC 3-lane 64 plates (Mimetas B.V, MI-OR-BV-02)	Vascular Leakage Assessment, Cell Viability and Snake Venome Testing	[39]
Murine brain microvascular EC	Diseases Model	Organoplate® 2-lane platform	Evaluation of Oligonucleotide-Induced Platelet Aggregation, Inhibition Test Using SYK Inhibitor, Effect of Perfusion Method	[40]
**Heart-on-chip**				
hiPSC-Derived Cardiomyocytes (hiPSC-CMs), Human Adult Cardiac Fibroblasts (CFB) and Human Induced Pluripotent Stem Cell-Derived EC (hiPSC-ECs)	Healthy model	PDMS and an injection molding-like technique with negative replicate molds and integration of 3D-printed pyrolytic carbon electrodes	Chronotropic Response to Isoprenaline, Eletrical Stimulation and Cell Viability	[20,22]
hiPSC-CM and CFB	Healthy model	Thermally bonding a bottomless well plate to the electrode-embedded base, ensuring that the microwires were properly aligned within each well (bioware II platform)	Tissue Maturation, Contractility, Electrical Stimulation and Drug Testing	[41]
hiPSC-CM, CFB And Primarily Epicardial Cells	Ischemia-reperfusion injury (IRI) model	Biowire II platform	Active Tension; Active to Passive Tension Ratio; Contraction Slope; Excitation Threshold; and Maximum Capture Rate; Immunostaining and Cell Tracking	[42]
hiPSC-CM	Healthy model	soft lithography to create PDMS microfluidic channels	Delivery Of mRNA, The Contractile Functionality and Maturation of The CM	[43]
hiPSCS-CM, hiPSC-EC and Human Brain Vascular Pericytes (hBVP),	Healthy and inflammatory model	Commercially available AIM biotech microfluidic chip	Vascular Network Formation, Perfusion, Anastomosis, Contractile Force	[44]
hiPSCS-CM	Healty model	Hydrogel scaffold and soft lithography to create PDMS microfluidic channels es	Hormone Stimulation Test, Cell Viability, Morphology Assessment, Optical and Electrical Performance	[45]
hiPSC-CM, CFB	Healthy model	PDMS, Hydrogel pillar using a combination of photolithography and standard microfabrication techniques	Contractile Forces, Electrophysiological Signals and Drug Testing	
hiPSC-CM, CFB	Diseases model	A 3D-printed heart-on-a-chip platform that incorporated thermoplastic elastomer/quantum dot nanocomposite wires	Exposure of the cardiac tissues to severe acute respiratory syndrome coronavirus 2 (SARS-Cov-2) at Various Multiplicities Of Infection, Contractile Force, Calcium Transients and Effects of Extracellular Vesicles (EVs)	[46]
hiPSC-CM, CFB ECs, SMC	Healthy model	soft lithography to create PDMS microfluidic channels with four chambers containing flexible micropillars	Contractile Force, Cell Viability and Transcriptomic	[23]
hiPSC-CM	Healthy model (effect of gravity)	embedded in a composite hydrogel scaffold of decellularized ECM (dECM) and reduced graphene oxide (rGO). then placed in custom-designed, gas-permeable chambers equipped with giant magnetoresistance (GMR) sensors for real-time monitoring	Contractile Dysfunction, Mitochondrial Damage, Transcriptomic and Effect of Gravity	[47]
H9C2 Rat Cardiomyoblasts	Diseases model (Hypoxia)	soft lithography to create PDMS microfluidic channel	Evaluation of Myocardial Ischemia Markers (Adenosine, ADP, and Lactic Acid)	[48]
Cardiomyocytes (CM) and CFB	Diseases model (TGF-β1)	Janus structural color film (SCF) and a microfluidic system. First, the Janus SCFs were fabricated using a template method that involved creating colloidal crystal templates through a vertical deposition approach, followed by the replication of microgroove patterns on a PDMS film	Collagen Deposition, Contractility, and Electrophysiological Properties	[49]
**Brain-on-chip**				
Human cerebral microvascular EC, pericytes and astrocytes (hAs)	BBB model for barrier insults	A microfluidic chip composed of a glass-ITO coverslip with patterned electrodes, a vascular microchannel layer, a porous PET membrane, a PDMS interlayer, a PET-Pt electrode layer, a hanging-drop layer, and two open reservoirs	Transendothelial Electrical Resistance (TEER) measurements, permeability measurements, live imaging, P-gp activity on-chip, immunofluorescence and confocal imaging, RT-qPCR, numerical simulations	[24]
Vero E6, Human Pulmonary Alveolar Epithelial Cells (HPAEpiCs), Human Umbilical Vein Endothelial Cells (HULEC-5a), Human Brain Microvascular Endothelial Cells (HBMECs), Human Adipocytes cells (HA), Human Microglial Cells (HMC3), hiPSC-PCs and Human Primary Peripheral Blood Mononuclear Cells (PBMCs)	BBB model for injury and neuroinflammation induced by SARS-CoV-2	Soft lithography to fabricate two interconnected microfluidic chips, each consisting of two channels separated by a fibronectin-coated porous PET membrane	Immunofluorescence imaging, qRT-PCR, PBMC adhesion assay, permeability assay, multiplex assay for cytokine detection, western blot, RNA-seq, Transmission Electron Microscopy (TEM)	[50]
Human brain microvascular EC, pericytes and hAs	BBB model for postoperative delirium	A PDMS microfluidic chip constructed by soft lithography and featuring two layers, assembled by plasma bonding, separated by a PETE membrane	Immunofluorescence and live cell imaging, TEER measurements, flow cytometry, NanoString, microglia-vasculature contact analysis, 5-choice serial reaction time task (5-CSRTT)	[51]
Human Cardiovascular Endothelial Cells (HCMEC), human hAs, iPSC-NPCs	neurovascular unit (NVU) model for neuroinflammation	Thermal curing to fabricate a PDMS microfluidic chip coated with Matrigel, featuring two vertically separated parallel channels	Measurement of TEER value, permeability test, measurement of proinflammatory cytokines	[52]
patient-derived iPSC lines with LRRK2 G2019S mutation, primary human brain vascular pericytes, iPSC-derived hAs	BBB model for Parkinson's disease	Commercial OrganoPlate 3-lane 40 platform	RNA-seq, GSEA analysis, BBB-chip barrier integrity assay, transwell barrier integrity assay, immunofluorescence and immunocytochemistry, RT-qPCR, confocal imaging, western blot, angiogenesis membrane array, ELISA	[25]
Human brain microvascular EC, immortalized Human Microglia–SV40 cell line (IMhu), ReN VM human neural progenitor cells	NGV model for neurodegeneration	Thermal curing to fabricate a microfluidic chip consisting of three plasma-bonded layers: two PDMS layers and a bottom glass slide	Measurement and estimation of vascular permeability, analysis of cell viability, immunofluorescence, western blot, cytokine level analysis, detection of oxidative modifications of proteins, measurement of H2O2 production, calcium imaging of neurons, detection of ROS production	[26]
Primary mouse brain microvascular EC	BBBoC model with immunosensors for cytokine secretion monitoring	DigiTACK device composed of two components reversibly sealed. Component 1: NPN membrane chip mounted onto an acrylic membrane and bonded to a PDMS thin-film channel using a PSA; Component 2: a glass wafer equipped with arrayed digital sensor patches fabricated via DRIE and micropatterning.	DigiTACK longitudinal assay, data analysis by a convolutional neural network immunofluorescence staining, COMSOL finite element analysis	[53]
**Liver-on-chip**				
Primary human hepatocytes (PHH), HepaRG, HUVEC, EC	Healthy	PhysioMimix® 3D MPS comprising a LOC device	Liver function markers, cell viability, metabolic activity	[20]
PHH, HUVEC, HepaRG,	Healthy	Direct contact (DC) platform by assembling a microfluidic channel layer and a tank layer for direct cell-to-cell contact and ECM device with 100 μm collagen-fibrin layer between hepatocytes and endothelial cells	Cell viability, endothelial markers, formation of circular channels	[54]
PHH, HepaRG, hiPSCs-derived hepatocyte-like cells (HLCs)	Healthy	Double compartment S1 liver microfluidic chip (Chip S1TM) (Emulate Bio)	Hepatocyte viability, maturation, drug response	[21]
Hepatocytes, Kupffer cells (KCs), EC, hepatic stellate cells (HSCs (LX-2))	Diseased (NASH)	Hydrogel triplet microchannel device with central channel filled with collagen hydrogel containing hepatocytes (HCs), HSCs, and KCs, surrounded by lateral channels lined with liver sinusoidal endothelial cells (LSECs) fabricated by photolithography	Lipotoxicity, liver inflammation, drug response	[55]
HepaRG, human hepatic sinusoidal EC (HHSECs)	Healthy	Triangular prismatic structure based on the hepatic acinus with layers made of PMMA plates combined with silicone membranes by means of Computer-Aided Design (CAD) modelling and Computer Numerical Control (CNC-assisted) manufacturing; 3D microneedles (2 μm); chip integrated into a perfusion system	Hepatocyte function, formation of sinusoids, metabolic biomarkers	[56]
PHH	Healthy	Recirculating mili-fluidic liver tissue chip (LTC) made of cyclic olefin copolymer (COC), consisting of two chambers: one for cell culture coated with type I collagen and fibronectin, and the other for oxygenation, with an integrated pumping system with piezoelectric pumps	Hepatocyte function, albumin (ALB) production, enzyme activity	[57]
HepG2, LX-2, EA. hy926 (EC)	Diseased (fibrosis)	ofluidic chip made by soft lithography and PDMS replica molding and with two chambers (parenchymal layer (containing hepatocytes and HSCs) and vascular layer (with LSECs and Kupffer cells)) separated by a porous membrane	Fibrosis progression, drug response, ECM stiffness, cell viability	[58]
**Gut-on-chip**				
Caco-2 cells (derived from human colon adenocarcinoma)	Healthy	OrganoPlate: a membrane-free 3D system perfused with 64 tissue model microfluidic chips (each with three different channels: the centre channel filled with ECM and the side channels used for perfusion and cell culture) incorporated into a standard 384-well plate	TEER (intestinal barrier integrity), cytotoxicity (LDH release), cell permeability (DRAQ7 staining), cellular morphology changes, cytoskeletal reorganization	[59]
Caco-2 cells (epithelial barrier in upper channel), HUVEC (simulate endothelial layer in lower channel)	Healthy	Organ-Chips S-1® made of PDMS with 2 parallel channels (upper and lower separated by a 50 μm thick porous membrane with 7 μm pores and coated with ECM) overlapping and separated by a porous membrane	ADME characteristics metabolic enzyme activity, drug transporter function (P-gp), barrier integrity (apafant test), metabolic clearance	[60]
Caco-2, LS 174T	Healthy	Single-channel GOAC model made by a custom mold using 3D printing, where PDMS is poured and moulded to form the structure of the chip and coated with a solution of type I collagen and Matrigel; connection to a microfluidic pump	Mucin Quantification (MUC2, MUC5AC), ELISA Assays (for mucus secretion), Cell Viability and Secretion Patterns (MUC2 vs. MUC5AC)	[61]
Caco-2	Healthy	PDMS microfluidic device with three layers: apical layer, porous membrane (7 μm) and basolateral layer; lining of channels with Matrigel	Barrier Integrity (Monolayer formation), Permeability Assays (dextrane, insulin, octreotide), TEER, Flux Measurement, Effect of Permeability Enhancers (sodium caprate, sucrose monolaurate)	[62]
Caco-2 cells	Healthy	OrganoPlates 3-lane 3D microfluidic chip (Mimetas BV) with 3 microfluidic channels (apical channel (Caco-2 cells), ECM made of type I collagen in the central channel and a basolateral channel) in a standard 384-well plate configuration	TEER, LDH activity, IL-8 secretion, Immunofluorescence staining	[63]
Colonic organoids	Diseased (Intestinal Aging)	3D PDMS microfluidic chip (GoC model) with upper and lower channels (2 mm wide and 0.25 mm high) separated by a porous membrane coated with type I collagen	TEER, cell viability, cell markers (p16, p21), qPCR for gene expression, Immunofluorescence (for cell markers such as MUC2, Occludin, ZO-1)	[64]
Caco-2 (human colon adenocarcinoma cells)	Healthy	Microfluidic device made of PDMS with upper and lower microfluidic channels (2.0 mm wide and 0.25 mm high) separated by a central porous membrane (20 μm thick, pores 5 μm in diameter), equipped with a three-electrode electrochemical biosensor (worker, reference and counter electrode)	CEA secretion (measured by electrochemical biosensors), cell viability (LIVE/DEAD assay), epithelial barrier integrity (TEER, ZO-1 expression), immunofluorescence (Ezrin), structure (intestinal villi formation), dynamic monitoring of CEA secretion over time	[65]
**Lung-on-chip**				
NCI-H1703 (epithelial type I), NCI-H441 (epithelial type II)	Healthy	Formation of microchannels and cavities for perfusion of the culture medium by 3D multi-jet printing with photocurable resin and deposition of PDMS in the mold; fibroblasts, NCI-H1703 and NCI-H441 incorporated into the collagen matrix (structure approximately 8 μm thick)	Cell viability, TEER, histological analysis, biomarker expression	[66]
Human alveolar epithelial cells (HPAEpiC), HUVEC, and macrophages (THP-1)	Diseased (COPD)	Lung-on-a-chip device made with a network of microfluidic channels (upper (air chamber with HPAEpiC) and lower (perfusion chamber with HUVEC)) and a porous membrane made of PDMS coated with ECM (collagen type I and fibronectin) simulating the air-liquid interface (ALI)	Cell viability, TEER (barrier function), permeability, inflammation (IL-6, TNF-α, MCP-1), ROS levels, AAT expression, monocyte adhesion, nanoparticle internalization	[67]
Human alveolar epithelial cells, macrophages differentiated from THP-1 cell line	Diseased (Inflammation)	AX12 microfluidic chip (AlveoliX AG) made of biocompatible silicone membrane with an ultra-thin porous membrane connected to the electro-pneumatic control units (AXExchanger, used for media exchange, basolateral sampling and TEER measurements, and AXBreather, used to apply 3D cyclic stretching) via the AXDock	Glucocorticoids (Budesonide) responses on inflammation (IL-6, IL-8), epithelial barrier disruption	[68]
Pulmonary vascular EC, type I and II alveolar cells, monocyte-derived macrophages	Diseased (Infection)	Microfluidic devices (ALoCs) made of PDMS and with parallel microfluidic channels (one apical coated with collagen IV, fibronectin and laminin for alveolar cell culture and the other basal coated with collagen IV and fibronectin for endothelial cell culture), separated by a porous PDMS membrane, air-liquid interface (ALI) and mechanical stretching	Cytokine and chemokine expression (IL-6, IL-8), immune responses, macrophage infection by M. fortuitum	[69]
**Kidney-on-chip**				
RPTECs, HUVEC, primary monocytes	Healthy and Diseased (renal inflammation and injury mediated by the immune system)	OrganoPlate 3-lane Narrow ECM microfluidic device made from PDMS with a polymerised type I collagen matrix (central channel are 200 μm × 165 μm), upper and lower channels are 300 μm × 165 μm (width x height) incorporating RPTECs and HUVECs in an oscillating perfusion system.	TEER, cytokine release (IL-6), WST-8 and LDH viability assay, immunofluorescence, monocyte adhesion/migration, intercellular adhesion molecule 1 (ICAM-1) expression, morphological changes	[70]
immortalized human renal proximal tubule epithelial cells (RPTEC/hTERT1)	Diseased (nephrotoxicity)	3D microfluidic platform in triple channel polycarbonate (TSC chip) (Nortis Bio), with ECM of type I collagen and fibronectin and porous membrane in polytetrafluoroethylene (PTFE) with 3 μm pores; apical side (proximal renal tubule formed by RPTECs and hTERT1) and basal side (vascular layer formed by endothelial cells)	Kidney injury biomarkers (kidney injury molecule-1 (KIM-1), cystatin-C, clusterin, NGAL, osteopontin (OPN)), inflammatory markers (IL-6, IL-8), ATP, LDH, Caspase activation (3, 8, 9, 12), cytochrome C, live/dead staining, tubular morphology	[71]
HRMVECs, hRPTECs	Diseased (Cardiorenal Syndrome - CRS)	Microfluidic perfusion system made of PDMS and moulded to form microfluidic channels (epithelial layer with hRPTECs to represent the proximal tubule and endothelial layer with human renal microvascular endothelial cells (HRMVECs) to represent the vascular system) and with a porous membrane coated with collagen and fibronectin	Renal injury markers (IL18, LCN2, HAVCR1), TGF-β pathway analysis, miRNA profiling, cystatin C levels, Gene expression (qRT-PCR), EV uptake (confocal microscopy), cytokine release, renal fibrosis markers	[73]
hRPTECs, HUVEC	Healthy and Diseased (nephrotoxicity)	Polycarbonate device with an apical chamber (hRPTECs, renal tubule) and a basolateral chamber (HUVECs, vascular system) connected by a microfluidic pathway (PET membrane with 3 μm pores and coated with a type I collagen ECM)	TEER, glucose reabsorption, KIM-1, NGAL, cell viability under shear stress (0.13 dyne/cm^2^)	[74]
Primary human renal progenitor cells, human EC, primary human white blood cells	Diseased (Acute Kidney Injury - AKI)	OrganoPlate 3-lane (Mimetas) made of PDMS with 3 channels: C1 channel (luminal) (human renal epithelial cells), C2 channel (interstitial) (type I collagen matrix), C3 channel (vascular) (human endothelial cells)	Immune cell migration, barrier integrity (fluorescence-labeled dextran), protein expression (ZO-1, Na-K-ATPase)	[75]
Mouse PCT cells, mIMCD-3 cells	Diseased (ADPKD)	Chip made of PDMS with a collagen matrix and microfluidic channels made by photolithography and filled with PDMS (80 μm wide)	Flow shear stress, permeability (fluorescent dextran), tubule dilation, cellular organization	[76]
**Skin-on-chip**				
Normal human dermal fibroblasts and epidermal keratinocytes, and HUVEC	Vascularized skin model	Perfusable devices consisting of nylon wires strung across connectors and filled with type I collagen	Histological analyses, measurement of skin permeability, systemic transport of benzo[a]pyrene pollutant	[77]
HUVEC, human primary pericytes, HCA2 fibroblasts, N/TERT cells	Micro-vascularized human tissue equivalent model	Microlithography to fabricate a PDMS microfluidic chip on a glass coverslip constituted of three parallel channels separated by micro-posts and connecting media reservoirs	Vasculogenesis assay, on-chip immunological staining	[78]
Human N-TERT1 keratinocytes, human primary foreskin-derived dermal fibroblasts	Full thickness skin equivalent	Four-units microfluidic device in which each unit was composed of five PMMA) layers thermally bonded together. Fluidic features were crafted using CNC micromilling.	Immunostaining, Two-Photon microscopy, confocal Raman spectroscopy, transepidermal electrical resistance and permeation assays	[79]
Human epidermal cells (keratinocytes and melanocytes), dermal fibroblasts, and dermal endothelia cells, MUTZ-3 progenitor cell line	Reconstructed human skin (RhS) with immune cells	A microfluidic device combining a modular multiwell adapter (MMA) with a commercial Costar 6-well plate. The MMA was composed of: Two CNC machined blocks, two patterned 142 μm-thick biocompatible double-side adhesive tape layers (ARseal 90880) and one high transparency and auto-fluorescence-free cycloolefin polymer (COP) 188-μm-thick layer (Zeonor 1420R)	Histology, immunohistochemistry, and immunofluorescent stainings, MTT assay, LDH assay, ICP-MS Measurements of NiSO4, flow cytometry	[80]
Human primary dermal fibroblasts and microvascular EC, human primary epidermal keratinocytes, human primary neutrophils	Vascularized immune-competent skin model	A perfusable microfluidic platform made of a collagen matrix placed between two plexiglass layers; a PDMS stamp with channel geometry was fabricated by photolithography and soft lithography and placed at the bottom of the top plexiglass	Vessel permeability measurement, immunofluorescence and confocal imaging, fluorescence *in situ* hybridization, immunohistochemistry, HSV detection, cytokine measurement	[81]
Primary rat sensory neurons, adult normal human keratinocyte **(**HEKs)	Innervated skin model	A PDMS microfluidic device fabricated through soft lithography on an SU-8 patterned wafer, forming two channels filled with collagen	Immunofluorescence, qRT-PCR, calcium imaging, ROS measurements, epidermal permeability assay	[82]
Immortalized human HaCaT keratinocytes, dermal fibroblasts, Propionibacterium acnes	Inflammatory skin model	A microfluidic platform made by cast PDMS and composed of four major layers and a PET membrane	Histological and immunofluorescence analysis, TEER measurement and skin permeation, cytokine detection, tissue cellular viability	[83]
**Cancer-on-chip**				
Primary cancer cells and stromal cells, and U937	Pancreatic cancer	Thermal curing to fabricate a microfluidic chip consisting of two PDMS chambers separated by a porous polyester membrane	Proliferation assay, immunostaining, qPCR, IC50 and drug perfusion test	[84]
SUM-159 breast cancer cells and primary CAFs	Breast cancer	Thermal curing to fabricate a PDMS microfluidic device with two concentric chambers	Invasion assay in time-lapse imaging	[85]
Breast tumor cells MDA-MB-231 and HUVEC	Breast cancer	A bioprinted microfluidic channel integrated atop a customized Transwell membrane. The membrane, magnetically aligned, was attached to a microplate made of a PDMS-PAAm composite	Immunostaining, TEER measurements	[86]
non-small cell lung cancer (NSCLC), CAFs, CD8^+^ T cells	Diseased (Cancer)	3D moulded LoC device (PDMS pour) with microchannels and microporous polycarbonate membrane with pores (approx. 5 μm)	Tumor responsiveness to anti-programmed death-1 antibody (anti-PD-1) therapy, immune response	[87]
A549-RFP, HUVEC-GFP	Diseased (Cancer)	Microfluidic chip manufactured by the demolding method for its layers with top layer (0.6 mm high and 0.6 mm wide) and bottom layer (1.4 mm high and 0.6 mm wide) separated by a porous SBS/PDMS nanofibre membrane manufactured by soft lithography	Cell invasion, HUVEC apoptosis, cell adhesion and proliferation	[72]
HFL-1, HUVEC, HCC827	Diseased (Cancer)	Resin molds via 3D printing with top layers of PDMS, microporous polycarbonate membrane with 5 μm pores, and bottom layer of PDMS	Epithelial-mesenchymal transition, drug resistance, cytokine secretion (IL-6)	[88]
NHLF and HUVEC	Lung cancer	Thermal curing to fabricate a microfluidic system composed of a PDMS base attached to a glass coverslip, paired with a small rectangular piece of porous ePTFE membrane secured within a U-shaped acrylic holder	Immunocytochemistry, permeability analysis, SEM imaging, porometry, live/dead assays	[89]
MCF7 breast cancer cells, and HUVEC	Breast cancer	Thermal curing to fabricate a three-layer PDMS microfluidic device with concentric circular regions, separated with trapezioidal microposts	Invasion and intravasation assays, immunofluorescence, permeability analysis, time-lapse imaging, quantification of angiogenic factors	[90]
SKOV3 cancer cells and HUVEC	Ovarian cancer	PDMS microfluidic chips fabricated with a 3D-printed SLA mold, consisting of a coverglass and two PDMS layers incorporating microchannels and perforations; pV4D4 polymer was utilized to create hydrophobic surface-coated culture plates for spheroids fabrication, using the iCVD process	qRT-PCR, transwell migration assay, extravasation assay, cytokine array, proteomics, NanoflowLC-ESI-MS/MS analysis	[91]
Human monocytes, MDA-MB-231-BoM 1833 human breast carcinoma cell line and SH-SY5Y cells	Breast cancer	Microfluidic components fabricated from PDMS using custom-designed molds, 3D-printed with a Form 3 printer and Grey V4 resin	Immunocytochemistry, flow cytometry, ELISA assay, quantification of bone resorption, preoteomic analysis, protein array	[92]
HUVEC, MCF7 and MDA-MB-231 breast cancer cells, THP-1, and TALL-104 T-cells	Breast cancer	Fabrication of the microfluidic device in three steps: methacrylation of a glass coverslip, polymerization of PAm hydrogels, and photopatterning of cell-laden GelMA hydrogels	Mass transfer study, hypoxia detection, cell infiltration and proliferation, flow cytometry, chemokine analysis	[93]
HUVEC, NHLF, human primary monocytes, MDA-MB-231 breast cancer cells and MDA-MB-435 melanoma cancer cells	Breast cancer and melanoma	Thermal curing to fabricate a PDMS microfluidic device consisting of four channels, each connected to two reservoirs and surrounding three rectangular compartments	Permeability measurements, flow cytometry, transwell migration assay, immunostaining	[94]
MDA-MB-31 cancer cells, primary human: fibroblasts and breast cancer cells, CAR-T cells and hiPSCs generated from human PBMCs	Breast cancer	Two stacked microfluidic channels microstructured using PDMS replica molding from two distinct master wafers created through photolithography and supported by a glass base	Immunofluorescence with confocal imaging, cytokine quantification assay, flow cytometry	[95]
HUVEC, NHLF, SKOV3 and A594 cancer cells	Ovarian carcinoma and lung adenocarcinoma	Thermal curing to fabricate a PDMS hydrogel-laden microfluidic device	Vessel permeability test, diffusivity measurements, chemotherapeutic test, efflux assays, 3D P-gp inhibitor assays, immunostaining, flow cytometry, western blot	[96]
**Spleen-on-chip**				
Red Blood Cells (RBCs), infected RBCs	Diseased	Microfluidic device	Transit Time, Deformation Rate, RBC Retention, Pressure Gradient and RBC Suspension Passage Rate.	[97]
RBCs (normal and sickle), PBMC-derived macrophages	Diseased (Sickle Cell)	Microfluidic OoC	Phagocytosis Rates, RBC Rigidity and Molecular Signals (e.g., CD47/SIRPα).	[98]
RBCs (normal and sickle), macrophages, PBMCs	Diseased (Sickle Cell)	soft lithography to create PDMS microfluidic channels	RBC Retention, Phagocytosis Rates, Effect of Hypoxia on RBC Rigidity and Reoxygenation Effects.	[99]
**Placenta-on-chip**				
Human trophoblast stem cells (hTSCs): cytotrophoblasts (CT), syncytiotrophoblasts (ST), and extravillous CT (EVT) and HUVEC	Healthy Model	soft lithography to create PDMS microfluidic channels and After curing, the upper and lower layers of the chip, each containing microchannel, were prepared and assembled with a PET nuclear pore membrane sandwiched between them.	Integrity of the Placental Barrier, human chorionic gonadotropin (hCG) secretion, Analyze gene expression changes in response to fluid shear stress and Exposure to mono-2-ethylhexyl phthalate (MEHP).	[100]
hiPSC-derived trophoblast	Healthy Model	OrganoPlate	Barrier Integrity Assay, Analyze Gene, Expression Changes and Protein Expression.	[101]
Placental vascular EC (PVECs), decidual cells (DECs), CT, STs, placental stromal cells (STRs), and HUVEC	Healthy Model	soft lithography to create PDMS microfluidic channels	Effects of endocrine-disrupting compounds (EDCs). Cell viability, Oxidative stress through glutathione (GSH) levels, Hormone production (e.g., β-hCG, progesterone) and cytokine profiles to assess inflammatory response.	[102]
HTR-8/SVneo cells and HUVEC	Healthy Model (Hypoxia Model)	soft lithography to create PDMS microfluidic channels	Trophoblast invasion and vessel permeability.	[103]
**Lymph node-on-chip**				
Lymphatic – ECs and blood - ECs derived from neonatal donors	Disease Model (lymphedema)	soft lithography to create PDMS microfluidic channels	Investigation of interstitial fluid drainage and the Effects of inflammatory cytokines.	[104]
Human dermal lymphatic microvascular EC	Healthy Model	soft lithography to create PDMS microfluidic channels	Lymphatic microvascular growth, Vessel diameter, Area coverage, Solute drainage rates and Immune cell recruitment.	[105]
Human fibroblastic reticular cells (FRC)	Healthy Model	soft lithography to create PDMS microfluidic channels	Migration of TNF-α Matured DCs and T-cells.	[106]
**Ovarian-on-chip**				
Human fallopian tube epithelium (HFTE)	Healthy Model	PREDICT-MOS microfluidic	Characterization of hFTE-derived sEVs (transcriptomics).	[107]
**Stomach-on-chip**				
Human-derived adenocarcinoma epithelial cell line MKN74 and the normal stomach fibroblast cell line NST-20	Healthy Model	soft lithography to create PDMS microfluidic channels	Barrier Function, Selective permeability and Pepsin activity assays.	[108]
**Pancreas-on-chip**				
Rat insulinoma-derived pancreatic β-cell line INS-1E and commercially available human pancreatic islet microtissues	Healthy Model	soft lithography to create PDMS microfluidic channels	Glucose-stimulated insulin secretion (GSIS) assays and the effects of antidiabetic drugs such as exendin-4 and tolbutamide.	[109]

Despite the significant progress and capabilities of OoC platforms, each model type has limitations that must be considered when interpreting results and designing experiments. LoC systems are useful for simulating drug metabolism, but they frequently struggle to sustain hepatocyte viability and complete metabolic functionality over long periods of time [20,21]. The technical complexity of HoC platforms is increased by the need for sophisticated electromechanical stimulation systems and precise cellular alignment to replicate cardiac contractility and electrophysiology [22,23]. Similarly, the ability of BBB models to mimic neuroinflammatory responses observed *in vivo* can be limited due to the absence of immune components such as microglia or circulating leukocytes [[24], [25], [26]]. Furthermore, pharmacokinetic and toxicity data can be skewed by material-related limitations, such as the use of polydimethylsiloxane (PDMS), which can result in the non-specific adsorption of hydrophobic medications and Nps [10,12]. Reproducibility and standardization across laboratories are further complicated by variations in cell sources, such as donor-to-donor heterogeneity in primary cells or variations in differentiation protocols for iPSCs [13,16]. Therefore, biological complexity and system scalability need to be carefully balanced, particularly for high-throughput applications and regulatory adoption.

### 2.1 Vessel-on-chip

The development of functional vascular networks is fundamental to advancing OoC technologies for both therapeutic regeneration and disease modelling applications [110]. Given that nearly every cell in the human body resides a capillary, the inclusion of vascular structures is essential for replicating native tissue physiology. In recent years, significant progress has been made in vascularizing a wide range of organ-specific systems. Microfabricated vessels—often referred to as “vessels-on-a-chip”—have emerged as a powerful platform that integrates microscale flow dynamics, tissue-level biomolecular transport, cell–cell interactions, and ECM environments under tightly controlled conditions. These systems enable the study of vascular biology in a context that closely mimics *in vivo* physiology. While vascularization is often considered a modular component in the construction of complex organ models, in some cases, vessel-on-chip platforms are treated as secondary structures. In other models, endothelial cells (EC) or vascular structures are incorporated into co-culture systems to enhance physiological accuracy and better replicate organ-specific microenvironments. Vascularized OoC models thus represent a critical advancement in the field, enabling more accurate simulation of both normal and pathological processes across a variety of human tissues. Their integration into multi-organ systems holds promise for improving the predictive power of *in vitro* models in drug development, toxicology, and regenerative medicine.

Orge et al. introduced a novel approach using specialized “vascular units” (VUs) to generate 3D microvascular networks within fibrin hydrogels [27]. The vascularized OoC system was fabricated by inserting an acupuncture needle into a fibrin precursor solution to create a hollow channel (∼300 μm in diameter), which served as a scaffold for VU integration. These VUs, composed of endothelial progenitor cells and organ-specific fibroblasts, exhibited robust angiogenic potential. Immunofluorescence imaging revealed that endothelial colony-forming cells (ECFCs) within the VUs organized into distinct layers and formed lumens, indicative of capillary-like structures. Furthermore, migration of hypoxia-inducible factor (HIF)-expressing cells contributed to stromal tissue formation within the hydrogel. The ratio of human umbilical vein endothelial cells (HUVECs) to fibroblasts was found to be a key determinant of vascular organization. A 5:1 HUVEC-to-fibroblast ratio resulted in enhanced expression of endothelial and ECM markers, including PECAM1 (CD31) and VEGF receptor 2 (KDR), compared to a 1:1 ratio, which led to ECFC depletion and reduced vascular integrity. These findings underscore the importance of cellular composition in optimizing vascular network formation and suggest that VUs can be integrated with other organ systems to enhance physiological relevance [27].

**Fig. 1:**
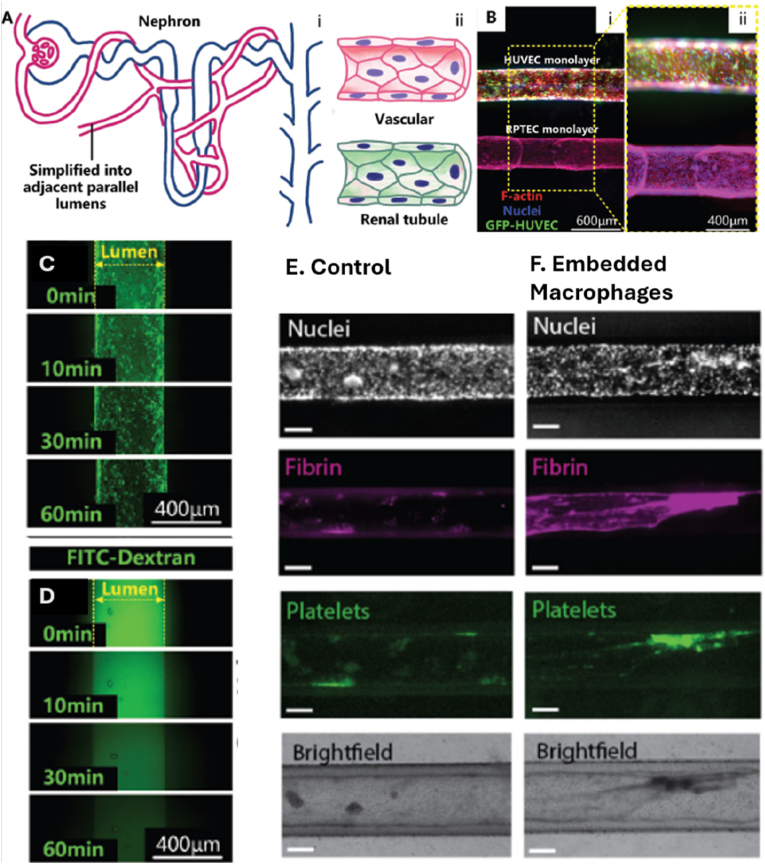
Demonstration of preliminary vascular-renal tubule interaction model constructed by the cell pre-wrapping seeding technique. **A:** Schematics showing (i) the nephron structure with renal tubules surrounded by vasculature, and (ii) a simplified parallel channel model used in the study. **B:** Fluorescence microscopy images at various magnifications showing the engineered model. HUVECs (green, GFP), F-actin (red), and nuclei (blue) are visualized. **C–D:** Time-lapse confocal imaging of FITC-dextran permeability through (C) endothelialized and (D) blank lumens, highlighting barrier function. Reprinted with permission from Ref. [14]; © 2024 The Author(s). Advanced Science published by Wiley-VCH GmbH. **E-F:** Blood perfusion assay results in a higher deposition of fibrin in the channels containing embedded macrophages; **E:** Blood vessel channel with a monoculture of hiPSC-EC (‘Control’). F. Blood vessel channel with co-cultured macrophages with a high % area coverage of fibrin; All scale bars represent 150 μm. Reprinted with permission from Ref. [36] © 2024 Advanced Materials Technologies published by Wiley-VCH GmbH. (For interpretation of the references to color in this figure legend, the reader is referred to the Web version of this article.)

Lou et al. developed a complementary strategy using a cell pre-wrapping seeding protocol to construct circular, perfusable tubular OoC models [28]. This method employed 3D-printed sacrificial sugar fiber templates to create branched and curved lumens. Renal proximal tubule epithelial cells (RPTECs) were encapsulated in a fibrin matrix and pre-wrapped around the sugar templates to ensure uniform cell adhesion. After hydrogel casting and template dissolution, the resulting lumens were lined with a continuous epithelial monolayer. This technique enabled the creation of co-culture models, including a vascular–renal tubule system, by printing parallel sugar fibers and pre-wrapping HUVECs and RPTECs on separate templates. Fluorescein isothiocyanate (FITC)-dextran permeability assays demonstrated that the endothelialized lumens exhibited significantly lower permeability than blank controls, confirming the formation of functional vascular barriers (Fig. 1) [28].

Creating healthy models is crucial for understanding processes, such as NPs transport and drug delivery, as well as replicating complex interactions that occur *in vivo*. However, it is equally important to consider the therapeutic effects in disease models. Therefore, Middelkamp et al. engineered a 3D blood vessel-on-chip model using human induced pluripotent stem cells (hiPSC)-derived EC and polarized human monocytic cell line THP-1 (THP-1) monocytes to study immune responses and thrombus formation [111]. The microfluidic chip, fabricated using PDMS-based soft lithography and a PMMA mold, featured six channels designed in SolidWorks. Co-culture with lipid-laden macrophages led to increased fibrin deposition, suggesting a pro-inflammatory environment conducive to thrombogenesis as show in Fig. 1E and F [111].

One of the main challenges is improving the complexity and maturity of these models to better mimic *in vivo* conditions. Enhancing reproducibility and standardization is also crucial for their widespread adoption. Future advancements may involve integrating vessel-on-chip models with other OoC systems to create comprehensive multiorgan platforms that can simulate complex interactions within the human body such as the blood circulation.

### 2.2 Heart-on-chip

**Fig. 2:**
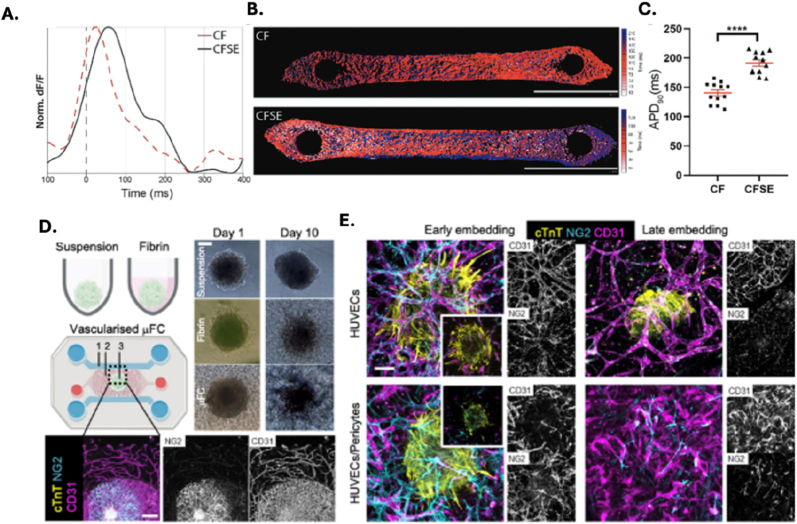
Schematic representation of cardiac models developed on HoC platforms. **A-C.** Electrical performance in CF- and CFSE-μEHTs. **A**. Representative changes in the fluorescence intensity of the FluoVolt-AP indicator over time in CF- (red) and CFSE- (black) μEHTs. **B.** Representative activation maps of CF- (top) and CFSE- (bottom) μEHTs. **C.** Action potential duration at 90 % (APD90) repolarization in CF- and CFSE-μEHTs stimulated at 2 Hz (unpaired *t*-test, n = 12 tissues/condition, from 3 batches). Data are presented as means ± SEM. ∗∗∗∗p < 0.0001 Reprinted with permission from Ref. [23]; © 2024 Advanced Healthcare Materials published by Wiley-VCH GmbH. **D-E**. Overview of embedding conditions. **D.** CMEF spheroids were cultured in ULA plates (suspension), in ULA plate with fibrin (fibrin) and in a microfluidic device (vascularized μFC). BF images show the spheroids at day 1 and day 10 of the experiments. Scale bar is 100 μm. **E**. Confocal z-projections of spheroids embedded at early or late stages, with vasculature formed by HUVECs or HUVEC/pericyte co-cultures under VEGF stimulation. Scale bar is 100 μm. Reprinted with permission from Ref. [112] © 2024 Scientific Reports published by PubMed Central. (For interpretation of the references to color in this figure legend, the reader is referred to the Web version of this article.)

HoC platforms have emerged as powerful tools for modeling a wide range of cardiac pathologies, including arrhythmias, fibrosis, and cardiomyopathies. These microengineered systems are designed to recapitulate the structural, mechanical, and electrophysiological properties of the human heart, while enabling real-time monitoring of biophysical and biochemical parameters. Importantly, HoC models can incorporate patient-derived iPSCs, allowing for the development of personalized disease models that enhance translational relevance and support the advancement of precision medicine. For example, Vivas et al. established a co-culture system using human iPSC-EC and cardiac microtissues to mimic human cardiac physiology [22]. Spontaneous beating of the cardiac microtissues was observed between days 3 and 7 post-seeding, with functional activity maintained for up to 30 days—an essential feature for long-term pharmacological and toxicological studies. In a follow-up study by Cofiño-Fabres et al., developed microengineered heart tissues (μEHTs) composed of iPSC-derived cardiomyocytes (CMs), EC, smooth muscle cells (SMCs), and cardiac fibroblasts (CFBs), cultured under flow conditions to promote self-organization [23]. The study demonstrated that the cellular composition of the tissue critically influences its structural and functional integrity. A CMs to non-CMs ratio of 60:40 yielded optimal contractile performance. Moreover, the inclusion of EC and SMCs significantly enhanced the electrical and mechanical properties of the μEHTs, as evidenced by increased contraction force and improved electrophysiological behavior, Fig. 2A–C [23].

Another interesting study by Di Cio et al., a novel approach by embedding cardiac spheroids, derived iPSCs, within vascularized microfluidic chips [112]. Most of the studies with OoC using heart tissues with this method is possible asses the cardiac function and toxicity in a more representative environment. Different protocols for embedding spheroids were explored, revealing that the timing of spheroid integration with vascular cells significantly impacts the resulting tissue architecture and functionality (Fig. 2D and E). The study also emphasizes the importance of using pericytes alongside EC to enhance the stability and barrier properties of the vascular networks. As a proof of concept, the toxicity of the therapeutic agent Vandetanib was evaluated using the developed model. Results indicated that in suspension cultures, the beat rates of spheroids dropped significantly after exposure to vandetanib, with rates decreasing from 29 ± 3 to 23 ± 1 beats/min after just 3 min at 1 μM10. In contrast, spheroids embedded in the microfluidic devices exhibited a transient drop in beat rates, but they recovered after 60 min, suggesting a protective effect of the vascularized environment [112].

Despite these advances, challenges remain—particularly in ensuring adequate oxygen and nutrient delivery to densely packed cardiac microtissues. Recent innovations, such as the integration of peristaltic pumps and vascularized microenvironments, show promise in addressing these limitations. Overall, HoC technology represents a significant leap forward in cardiac research. By enabling the study of complex disease mechanisms and therapeutic responses in a human-relevant context, these platforms are poised to transform drug development, disease modeling, and personalized medicine.

### 2.3 Brain-on-chip

The complex nature of the human brain, challenges in directly studying it in living organisms, and the significant disparities between animal models and human physiology hinder the understanding of brain functions, limiting progress in disease prevention and treatment [113]. Advancements in OoC technology has made it possible to create highly biomimetic brain models *in vitro* in both healthy and diseased states and offer an alternative to using human brains for a variety of experimental studies [114,115]. Current brain-on-chip (BoC) approaches focus on key areas such as the BBB, the neurovascular unit, neural signal transmission (specialized neural channel interfaces), and *in vitro* culture models derived from the differentiation of pluripotent stem cells, such as brain organoids. The BBB is a critical interface that regulates molecular exchange between the circulatory system and the central nervous system, playing a fundamental role in maintaining brain homeostasis. Its unique architecture comprises EC in the cerebral vasculature, pericytes embedded within the basement membrane, and astrocytic (hAs) that extend toward the vessel lumen, collectively forming a highly selective and dynamic barrier [116]. These platforms facilitated progress in fundamental research areas, including cell migration, the investigation of disease mechanisms (e.g., neurodegenerative diseases), drug permeability studies, and the pathology of SARS-CoV-2 [117,118].

Disruption of the BBB is a hallmark of numerous central nervous system (CNS) disorders, including stroke, neuroinflammation, and neurodegenerative diseases. BoC platforms offer a powerful means to investigate how the BBB responds to pathological insults in a controlled, human-relevant environment. In a notable study, Wei et al. developed a human-cell-based BBB-on-a-chip system that integrates transparent electrodes for real-time, high-resolution monitoring of barrier integrity. The platform incorporated multiple human cell types—cerebral microvascular EC, hAs, and pericytes (hPs)—co-cultured within a multilayered open microfluidic device. EC were seeded along the walls of a PDMS microchannel to form a monolayer mimicking the vascular endothelium. This channel was connected to open reservoirs that enabled gravity-driven flow via platform inclination. A porous polyethylene terephthalate (PET) membrane separated the vascular compartment from the brain compartment, where hAs and hPs were embedded in a hydrogel matrix to replicate the three-dimensional cellular architecture of the brain. The open design of the microfluidic chip allowed direct sampling from both sides of the barrier, facilitating detailed analysis of molecular transport and diffusion. Integrated indium tin oxide (ITO) and platinum (Pt) electrodes enabled continuous measurement of trans endothelial electrical resistance (TEER), providing dynamic insights into barrier formation and disruption. The transparency of the ITO electrodes also permitted live, high-resolution confocal imaging of the BBB structure and its reorganization in response to external stimuli.

**Fig. 3:**
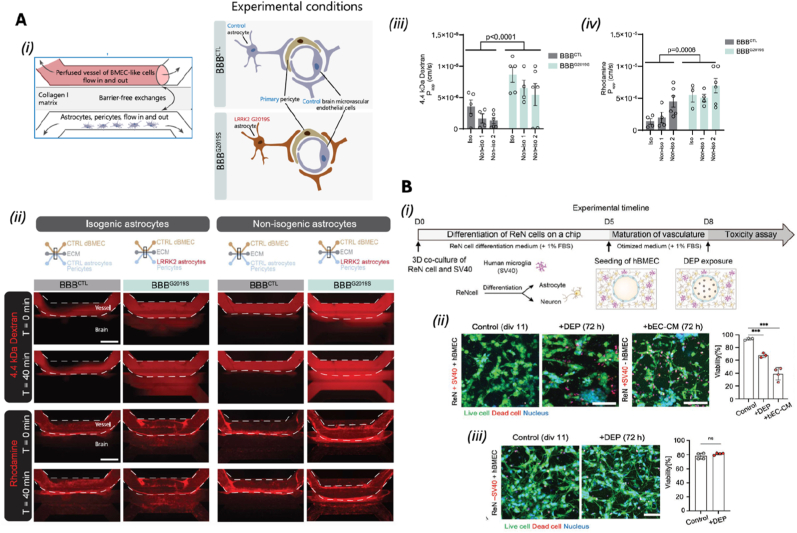
BoC models for studying neurodegeneration. **(Ai)** Schematic of the BBB chip and experimental setup. **(Aii)** Fluorescence images showing differential distribution of 4.4 kDa dextran-TMRE and rhodamine in BBB chips with the LRRK2 G2019S mutation (BBBG2019S) versus control (BBBCTL). **(Aiii–iv)** Quantification of apparent permeability (Papp) for both tracers across chips derived from three iPSC pairs. Reprinted with permission from Ref. [25]; © 2023 Nature Communications. **(Bi)** Timeline for generating the neurovascular-glia (NGV)-on-a-chip model. **(Bii)** Exposure to diesel exhaust particles (DEPs) in the presence of microglia induced neurotoxicity and reduced cell viability; similar effects were observed with conditioned medium from DEP-exposed EC. (**Biii)** In the absence of microglia, DEPs did not significantly affect viability. Live/dead staining: Calcein-AM (green), PI (red). Reprinted with permission from Ref. [26] © 2022 Advanced Functional Materials published by Wiley-VCH GmbH. (For interpretation of the references to color in this figure legend, the reader is referred to the Web version of this article.)

To assess the BBB functionality and its ability to replicate barrier responses to traumatic events, oxygen/glucose deprivation (OGD) was applied to simulate cerebral ischemia. This insult triggered rapid cytoskeletal remodeling within the endothelial layer, characterized by the formation of prominent actin stress fibers, cell shrinkage, and compromised barrier integrity. Notably, TEER measurements revealed a decline in electrical resistance preceding visible structural changes, indicating that functional impairment of the barrier occurs early during ischemic stress. These findings highlighted the ability of BoC models to replicate key BBB functions *in vitro* and to study the dynamic reorganization of the barrier in response to external stimuli [24]. Choi et al. developed an NVU-on-a-chip model featuring vertically aligned microchannels seeded with human cerebral microvascular endothelial cells (HCMECs), iPSC-derived neural progenitor cells, and astrocytes. A graphene oxide-based aptamer sensor enabled real-time detection of proinflammatory cytokines. This platform provided a more physiologically relevant model of neuroinflammation than traditional animal systems, facilitating the study of CNS disorders and therapeutic screening [52]. De Rus Jacquet et al. employed a 3D BBB-on-chip (BBBoC) model using the OrganoPlate 3-lane system to study Parkinson's disease (PD). The model incorporated iPSC-derived astrocytes carrying the LRRK2 G2019S mutation, which exhibited a pro-inflammatory phenotype and disrupted vascular integrity (Fig. 3Ai). Inhibition of the MEK1/2 pathway restored barrier function, and the observed vascular changes mirrored those found in PD patient brain tissue, highlighting the model's translational relevance (Fig. 3Aii-iv) [26].

A key advancement in BoC and BBBoC models lies in the incorporation of sensor technology. These systems employ electrical, electrochemical, and optical biosensors to enable real-time tracking of various biological processes and markers. These include monitoring growth factor release, receptor and biomarker expression, immune cell activation, cell viability, interactions between cells, and the transport of drugs and NPs across the BBB [119,120]. In this context, Su et al. introduced an innovative platform called "Digital Tissue-Barrier-Cytokine-Counting-on-a-Chip (DigiTACK). This system integrated digital immunosensors into a tissue-like chip, allowing highly sensitive, multiplexed, and longitudinal analysis of cytokine secretion from cultured brain endothelial barriers. The DigiTACK device comprised two components designed to be reversibly sealed. Component 1 consisted of a nanoporous silicon nitride (NPN) membrane chip mounted onto an acrylic housing block. The bottom surface of this housing block was irreversibly bonded to a PDMS thin-film cutout channel using a double-sided pressure-sensitive adhesive (PSA). Together, Component 1 presented a Transwell-style open-well reservoir for luminal (blood) fluid or stimulation in the top compartment, and a bottom abluminal microfluidic channel compartment. These compartments were connected exclusively through the suspended NPN/cell membrane interface. Component 2 was a glass wafer equipped with digital sensor patches fabricated via deep reactive ion etching (DRIE) and micropatterning. Each sensor patch contained three rectangular arrays (89,760 microwells per array), positioned under the PDMS abluminal compartment, with each array designed to target a specific analyte. The soft PDMS thin-film channel layer in Component 1 acted as an O-ring, enabling reversible sealing to the Component 2 wafer. Prior to use, the NPN membranes in Component 1 were coated with gelatin to support the seeding of primary mouse brain microvascular EC. The digital sensors in Component 2 used a unique bead-free microwell approach to achieve rapid "digital fingerprinting" of the analytes, with detection limits as low as 100–500 fg/mL for mouse MCP1 (CCL2), IL-6, and KC (CXCL1). The DigiTACK platform is versatile and can be used to study temporal cytokine secretion in other barrier-based OoC systems. This capability provides valuable insights into the dynamic secretory behavior of the BBB using precisely controlled experimental setups [53].

Despite their promise, BoC technologies have progressed more slowly than other OoC systems, largely due to the inherent complexity of the brain. This complexity arises from the diversity and multifunctionality of neural cell types, the intricate architecture of neural circuits, and the region-specific structural and functional heterogeneity of the brain.

### 2.4 Liver-on-chip

**Fig. 4:**
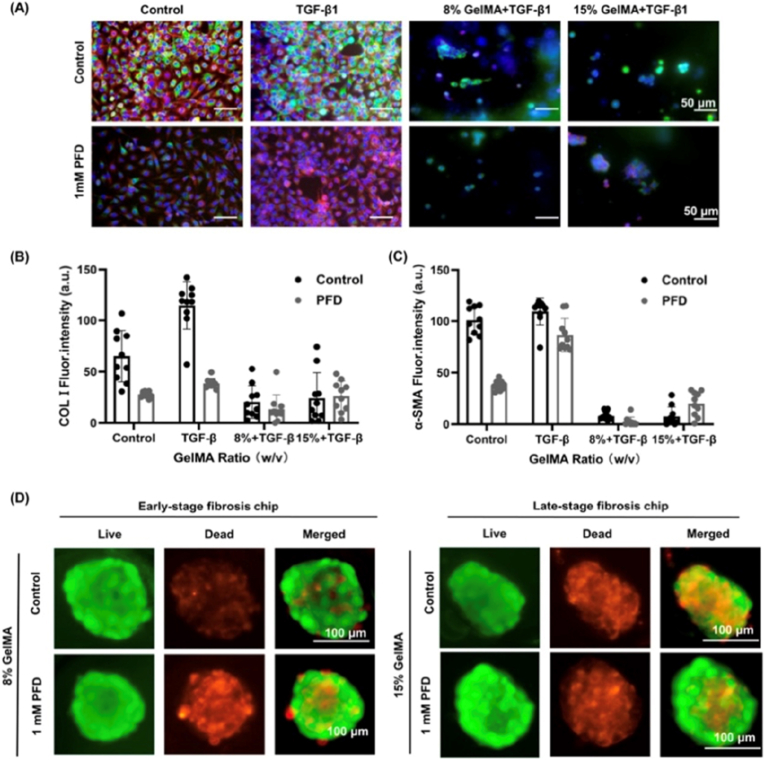
Drug resistance test was performed for 24 h after the liver fibrosis model was cultured for 5 days. **(A)** Representative images of LX-2 cells within different degrees of GelMA hydrogel after PFD treatment for 24 h Col1 (green), *α*-SMA (red), and nuclei (DAPI, blue) in HSCs (scale bars, 50 μm) and image-based quantification of Col1 **(B)** and *α*-SMA **(C)** The experiment was conducted with ten independent view samples (*n* = 10). **(D)** Fluorescent imaging of the HepG2 spheroid viability in an early-stage fibrosis chip (8 % GelMA) and a late-stage fibrosis chip (15 % GelMA) treated with vehicle (control) or PFD (1 μM) treatment. Green and red fluorescence represented living and dead cells, respectively. Reprinted with permission from Ref. [58] © 2023 ACS Biomaterials Science & Engineering, American Chemical Society. (For interpretation of the references to color in this figure legend, the reader is referred to the Web version of this article.)

Since the liver is the primary organ involved in drug metabolism and detoxification, LoC devices are extremely important for *in vitro* simulation of hepatic function [121,122]. Generally, LoC models employ co-cultures of human hepatocytes, as primary hepatocytes are the best alternative due to their metabolic capacity, which closely resembles human liver; however, their functionality in culture gradually decreases over time. Thus, a significant technical challenge is obtaining viable hepatocytes that can be maintained in long-term culture [123]. Kopp et al. developed a micro physiological LoC system (MPS-T12) to evaluate genotoxic responses using co-cultures of primary human hepatocytes (PHHs) or HepaRG cells with HUVECs embedded in a collagen I/fibronectin matrix. The 3D liver constructs were transferred to microfluidic plates and perfused to simulate hepatic blood flow. After stabilization, lymphoblastoid TK6 cells were introduced via Transwell inserts to assess DNA damage using micronucleus assays and Duplex Sequencing. Exposure to genotoxins such as methyl methanesulphonate (MMS) and benzo[a]pyrene (B[a]P) revealed chromosomal abnormalities and specific mutations. Functional markers including urea synthesis, albumin production, and cytochrome P450 activity confirmed the metabolic competence of the model, supporting its utility for genotoxicity testing in pharmaceutical research [20]. Zhang et al. utilized iPSC-derived human liver organoids (HLOs) to construct a LoC model for hepatotoxicity assessment. After 20 days of differentiation, organoids were dissociated and seeded into ECM-coated microfluidic channels. Continuous perfusion with hepatic growth medium supported maturation, as evidenced by increased albumin secretion and CYP450 expression. Upon exposure to hepatotoxic agents such as paracetamol and fialuridine, the model demonstrated dose-dependent toxicity responses. Single-cell RNA sequencing and biomarker analysis (ALT, AST) provided mechanistic insights, confirming the platform's relevance for high-throughput drug screening [21]. Liu et al. developed a LoC model to simulate liver fibrosis and evaluate antifibrotic therapies. The device featured two chambers separated by a porous membrane: one containing a vascular layer with Liver Sinusoidal EC (LSEC) and Kupffer cells, and the other a parenchymal layer with HepG2 hepatocytes and LX-2 hepatic stellate cells. Fibrosis was induced using TGF-β1, leading to ECM deposition, inflammatory cytokine release, and gene expression changes associated with fibrogenesis. The model enabled real-time monitoring of metabolic and inflammatory markers in the perfusate and was used to assess the efficacy of antifibrotic drugs such as sorafenib and pirfenidone. Furthermore, as illustrated in Fig. 4 the model allowed for the evaluation of how medications such as sorafenib and pirfenidone affected patients according to the stage of fibrosis [58].

In summary, the development of LoC models has increased the research of liver physiology, disease, and pharmacology. These models provide an alternative for drug screening, genotoxic testing, and the study of complex liver diseases such as fibrosis by simulating the hepatic milieu in a dynamic and physiologically relevant platform [20,21]. These models' ability to replicate vascular architecture and cellular interactions in a controlled microfluidic setting increases their prediction accuracy for human responses. The inclusion of diverse cell types and the use of 3D structures have improved the fidelity of liver functions, making these platforms invaluable for both basic research and the development of therapeutic strategies [54,124]. LoC models are positioned to become essential instruments for personalized medicine as the technology develops further with improvements in integration and miniaturization. They provide information on drug toxicity and efficacy in a more patient-specific setting [20,54,58].

### 2.5 Gut-on-chip

The intestinal lumen and circulatory system are replicated in parallel by the gut-on-chip (GoC), which is made up of microchannels separated by a microporous membrane that allows for the selective flow of nutrients and molecular signals [125,126]. These channels support the co-culture of intestinal cells, which form a barrier similar to the human intestinal epithelium, facilitating the study of processes as nutrient absorption, mucus secretion, and barrier function [125,[127], [128], [129]]. An important feature of GoC model is its ability to reproduce peristalsis through lateral pressure application, resulting in cyclic contractions that influence the morphology and functionality of epithelial cells, promoting the formation of 3D villi and the secretion of mucins [130,131]. Intestinal homeostasis depends on the formation of a cohesive epithelial barrier with tight junctions, which is made possible by these peristaltic motions and shear stress, which mimic the dynamic conditions of the intestine [125,127,132]. Furthermore, under continuous flow circumstances, the apparatus enables the co-cultivation of living microorganisms in close contact with intestinal cells, rendering it a useful tool for investigating intestinal permeability, drug absorption, and inflammatory reactions to viral and bacterial infections [128,130].

Morelli et al. developed a high-throughput GoC platform using the OrganoPlate, which integrates 64 microfluidic chips within a 384-well plate format. Each chip features three channels, with the central ECM-filled channel flanked by perfusion and cell culture channels. Caco-2 cells were cultured adjacent to the ECM to form an intestinal barrier under dynamic flow. Upon exposure to enterotoxins such as nigericin, patulin, ochratoxin A, and melittin, TEER measurements revealed dose-dependent barrier disruption. Cytotoxicity assays and confocal imaging showed increased permeability, LDH release, and actin cytoskeleton disorganization, particularly with melittin and ochratoxin A. The platform enabled detailed assessment of toxin-induced epithelial damage and morphological changes [59].

Carius et al. employed a dual-channel GoC device to investigate drug absorption and transporter function. The model featured Caco-2 cells in the upper (epithelial) channel and HUVECs in the lower (endothelial) channel, separated by a porous ECM-coated membrane. The system was connected to an automated perfusion module that applied continuous flow and cyclic strain to mimic peristalsis. The model demonstrated enhanced expression of metabolic enzymes and transporters, including P-glycoprotein (P-gp), compared to static 2D cultures. Drug permeability assays confirmed the formation of a functional epithelial barrier, although PDMS absorption of small molecules was noted as a limitation [60].

More representative *in vitro* models are needed since these devices make it possible to analyze components as the GoC flora. The ability of the GoC to replicate real-time interactions between different cell types and microbes makes it a valuable tool for scientific research [126,127,133,134].

### 2.6 Lung-on-chip

The LUoC model can be used as a biomedical platform for studying pulmonary diseases and developing treatments [72]. The main goal of LUoC devices is to simulate the dynamics of gas exchange and the respiratory movements that influence the interactions between alveolar epithelial and EC [72,135]. Compared to conventional *in vitro* or animal models, LUoC systems allow for more in-depth research into complex respiratory diseases, such as acute lung injury, fibrosis, and infections [66,136,137].

Kim et al. developed a LUoC model through soft lithography and 3D printing to produce a platform that simulates the physiological conditions of human lung tissue. First, a mold was created using a multi-jet 3D printer that utilizes photopolymerizable resin to fabricate the device. The PDMS was prepared in a 15:1 ratio of base to curing agent, poured into the mold, and cured at 60 °C for 5 h. Type I (NCI-H1703) and type II (NCI-H441) alveolar epithelial cell lines were obtained and cultured in RPMI-1640 medium supplemented with fetal bovine serum (FBS) and an antibiotic solution. Along with the two types of alveolar epithelial cells, fibroblasts were integrated into a collagen matrix to form a structure approximately 8 μm thick. The results demonstrated that the localization of the tight junction protein ZO-1 and the TEER values were elevated, indicating uniform cell distribution and intact barrier integrity. Pulmonary markers, such as tight junction proteins (ZO-1, occludin, E-cadherin), epithelial sodium channels (α-, β-, γ-ENaC), and surfactant proteins (SP-A, SP-B), showed high expression in gene expression analysis, and the presence of SP-B protein highlights the model's potential for research on respiratory conditions [66].

Gu et al. used single-cell RNA sequencing (scRNA-seq) to uncover molecular insights into lung barrier dysfunction by introducing a human LUoC model to study the immune response to co-infection with influenza A virus (IAV) and Streptococcus pneumoniae (SP). The device was composed of overlapping PDMS microfluidic channels separated by a porous PET membrane and seeded with alveolar epithelial cells (NCI-H441), HUVECs, and Peripheral Blood Mononuclear Cells (PBMC)-derived macrophages. The study demonstrated how co-infection compromised the integrity of the epithelial barrier by significantly down-regulating and mislocalizing the tight junction protein ZO-1. Cytokine analysis verified increased levels of inflammatory mediators, and scRNA-seq data also showed distinct transcriptional alterations in epithelial cells under co-infection circumstances. This model established that ZO-1 disruption is a key mechanism underlying lung injury during IAV-SP co-infection, showcasing the ability of LUoC platforms to dissect complex host-pathogen interactions at the cellular and molecular levels [138].

Kanabekova et al. developed an innovative approach to overcome limitations of conventional synthetic membranes in LUoC platforms by developing a hybrid nanofiber membrane composed of poly(ε-caprolactone) (PCL) and collagen. The morphology, porosity, and mechanical characteristics of this biomimetic scaffold were made to mimic those of the lung alveolar basement membrane. The membrane demonstrated excellent biocompatibility with lung fibroblast cultures when it was incorporated into a customized cyclic olefin copolymer (COC) and PDMS microfluidic chip. The ideal flow conditions inside the chip were validated by computational fluid dynamics (CFD) modeling. In addition to supporting 3D cell culture, the PCL–collagen membrane offered better structural and functional mimicry of the alveolar-capillary barrier, which could improve the physiological accuracy of LUoC systems used in drug testing and disease modeling [139].

In the study by Dasgupta et al., a human Lung Alveolus Chip was used to model radiation-induced lung injury (RILI). This platform combined primary human lung microvascular EC and primary alveolar epithelial cells cultivated at an air-liquid interface (ALI). The cells were then subjected to cyclic mechanical strain to simulate respiratory motion. The chip showed the typical signs of acute RILI after being exposed to 16 Gy gamma radiation, such as DNA damage (measured by 53BP1 foci), disruption of the barrier, cell hypertrophy, and increased levels of pro-inflammatory cytokines, particularly when PBMC were present. Additionally, the model showed increased expression of genes linked to the endothelial-mesenchymal transition (EMT) and oxidative stress markers, providing a human-relevant system for evaluating treatment interventions like lovastatin and prednisolone [140].

However, despite its advantages, such as precise control over microenvironmental conditions, improved simulation of tissue interfaces, and the ability to incorporate mechanical stimuli and immune components, the LUoC still has limitations to overcome. Reproducing the complexity of lung physiology especially regarding the links between the lungs and other organs such as the immune and circulatory systems, is a challenge [135,141]. Another drawback that limits the ability to mimic chronic circumstances is the difficulty of maintaining primary human cells for an extended period since cell viability is not yet completely guaranteed. Ongoing innovation is necessary to overcome current limitations in complexity, scalability, and cell viability, and future work should aim to integrate LUoC models into multi-OoC systems for comprehensive *in vitro* simulation of human physiology [87,136,142].

### 2.7 Kidney-on-chip

The kidney-on-chip (KoC) can imitate processes including glomerular filtration, tubular reabsorption, and metabolite release by integrating renal cells into a 3D structure [71,75] Research on drug toxicity, namely nephrotoxicity linked to chemotherapeutic therapies and other nephrotoxic drugs, has also been made possible by KoC models [71,74].

Gijzen et al. developed an immunocompetent KoC model of the human proximal tubule to investigate renal inflammation and immune cell migration. The device featured RPTECs in the upper channel and HUVECs in the lower channel, separated by a collagen-based ECM. Primary human monocytes were introduced into the endothelial compartment to simulate immune infiltration. Under bidirectional flow, the model recapitulated epithelial–endothelial interactions and inflammatory responses, enabling the study of monocyte migration and epithelial injury under pathological conditions [70].

Guimarães et al. engineered a spheroid-matrix KoC model incorporating co-cultured RPTEC and HUVEC in a 2:1 ratio, embedded within a gelatin–fibrin hydrogel that mimics the mechanical and biochemical properties of the native renal cortex. After being created using the hanging drop technique and cultivated for a maximum of 14 days, the spheroids showed improved diameter, viability, and spontaneous tubulogenesis, especially when co-cultured. Functional assays showed active albumin uptake and glucose reabsorption, reflecting important proximal tubule activities, immunofluorescence analysis validated the expression of renal markers (AQP1) and amphotericin B was used to challenge the system and measure nephrotoxic responses. With an increase in IC_50_ values, co-cultured spheroids showed noticeably higher resistance to amphotericin B than monocultures. Viability tests and live/dead imaging verified drug-induced toxicity, demonstrating a dose- and time-dependent cytotoxic response that included widespread apoptosis after 24 h and peripheral cell death after 3 h. The model was incorporated into a PDMS microfluidic chip, where spheroids preserved their structural and functional integrity for a full day while being continuously perfused at a rate of 2 μL/min. The system's applicability for dynamic drug testing was confirmed by the high spatial fidelity of nephrotoxicity replicated by subsequent exposure to amphotericin B under flow conditions [170]. Payasi et al. used a 3D KoC model to compare the nephrotoxic effects of a novel polymyxin B formulation (VRP-034) with a commercial counterpart. The chip incorporated RPTECs/hTERT1 and EC cultured on a collagen–fibronectin ECM and separated by a porous PTFE membrane. Continuous perfusion mimicked renal blood flow. Exposure to VRP-034 resulted in significantly lower levels of renal injury biomarkers (e.g., KIM-1, NGAL, cystatin-C), inflammatory cytokines (IL-6, IL-8), and apoptosis markers compared to commercial PMB. The study demonstrated that VRP-034 preserved tubular integrity and reduced cytotoxicity, supporting its potential as a safer therapeutic alternative [71].

**Fig. 5:**
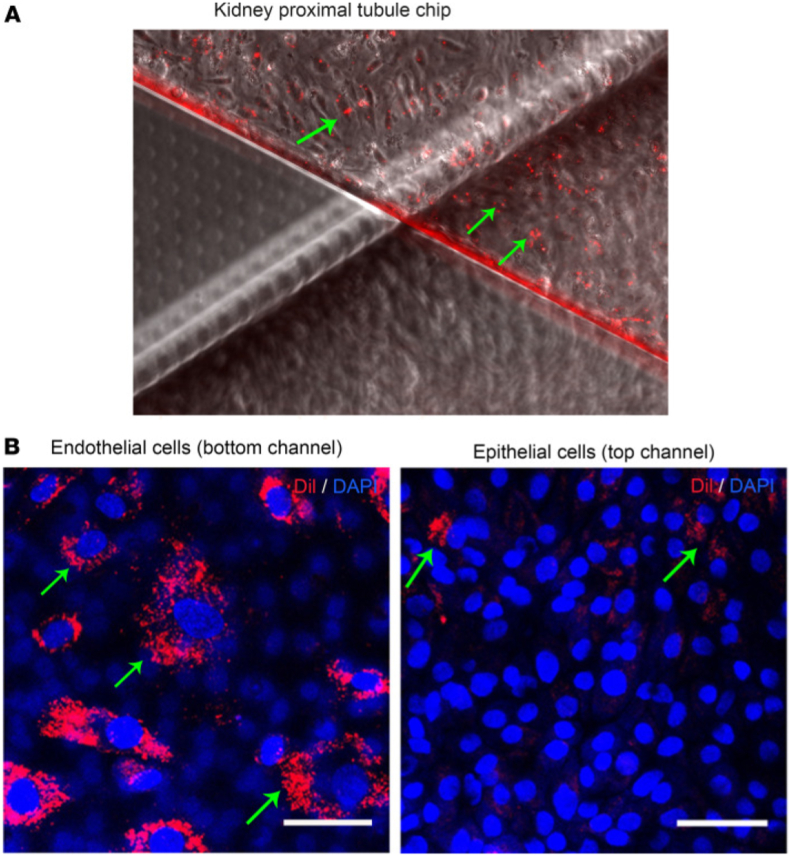
Successful dosing of EVs on kidney-on-chip. Dil-stained EVs from a healthy control were visualized after 3-day perfusion period using fluorescence microscopy. **(A)** Representative images of the fluorescently labeled EVs (red), overlaid with a phase contrast image of the chip, mainly seen in the vascular endothelial (bottom) channel (scale bar = 100 μm). **(B)** Representative fluorescent confocal images of the EVs, cells in the vascular endothelial channel (bottom) and cells in the epithelial (top) channel (scale bar = 100 μm). Reprinted with permission from Ref. [73] © 2023 Clinical Medicine, JCI Insight. (For interpretation of the references to color in this figure legend, the reader is referred to the Web version of this article.)

Chatterjee et al. investigated the effects of circulating extracellular vesicles (EVs) from patients with cardiorenal syndrome (CRS) using a KoC model. The platform featured hRPTECs and human renal microvascular EC (HRMVEC) cultured on a collagen–fibronectin-coated porous membrane. Exposure to patient-derived EVs induced endothelial dysfunction and epithelial injury, mediated by microRNA signaling. This model provided mechanistic insights into EVs-induced renal damage and highlighted the role of intercellular communication in CRS pathology (Fig. 5) [73].

Kim et al. developed a KoC platform to model renal function and evaluate nephrotoxicity induced by gentamicin and cisplatin. The device featured apical and basolateral chambers separated by a 3 μm PET membrane coated with type I collagen, supporting co-culture of hRPTECs and HUVECs. Under physiological shear stress (0.13 dyne/cm^2^), the model maintained epithelial barrier integrity and enabled functional assessments, including glucose reabsorption and TEER. Importantly, it facilitated detection of injury biomarkers such as KIM-1 and NGAL. This KoC system offers a physiologically relevant platform for studying renal toxicity and advancing drug development [74]. This study underscores the utility of KoC platforms in replicating renal physiology and detecting early signs of drug-induced injury. By providing a dynamic and human-relevant environment, KoC systems represent a significant advancement in renal research, offering new opportunities for drug development, toxicity screening, and the design of targeted therapies.

KoC platforms offer a dynamic and physiologically relevant model to study renal function, nephrotoxicity, and immune-mediated injury *in vitro*. These systems allow for real-time evaluation of drug toxicity, injury biomarkers, and transport function by using co-cultured human epithelial and EC to replicate important aspects of the proximal tubule [71,74]. Their ability to incorporate immune components has further expanded their utility in modeling renal inflammation and systemic conditions [70,73]. Despite their potential, the scope of current KoC models is still restricted; they frequently leave out crucial nephron segments and have issues with multi-organ integration, scalability, and standardization. However, ongoing improvements in biological complexity and design make KoC a potent tool for drug development and renal.

### 2.8 Skin-on-chip

The skin is the biggest organ of the human body, has vital functions such as regulating temperature, supporting metabolic processes like vitamin D production, and protecting internal organs from external hazards, including infections, microbes, UV radiation, and physical harms [143]. Additionally, skin holds great potential for transdermal drug delivery, which requires a comprehensive understanding of its structure [144]. Although animal models and 2D/3D *in vitro* systems are commonly used for studying skin diseases and drug testing, they struggle to fully replicate the intricate and dynamic architecture of real skin [143].

Skin-on-chip (SoC) systems are typically classified as either transferred or *in situ* models. Transferred models incorporate pre-formed skin constructs—such as biopsies or engineered equivalents—into microfluidic devices for dynamic culture, making them ideal for drug screening and toxicity testing. In contrast, *in situ* models support the direct formation of skin layers within the chip, offering precise control over tissue development and disease simulation [79,110,145]. A major challenge in skin modeling is the lack of vascularization, which limits nutrient delivery and waste removal. Recent advances have addressed this limitation [5]. Salameh et al. developed a fully vascularized SoC model featuring perfusable vessels and angiogenic capillary networks embedded in a fibroblast-laden dermal matrix. The model supported topical and systemic drug testing, demonstrating enhanced permeability and physiological relevance [78]. Similarly, Sriram et al. introduced a versatile SoC platform composed of thermally bonded PMMA layers with integrated microporous membranes and microfluidic channels. The system supported co-culture of human fibroblasts and N/TERT keratinocytes on a PEG-fibrin hydrogel scaffold and could operate in multiple configurations—as an open system, a sealed bioreactor, or an *in vitro* analysis system (IVAS). Compared to static cultures, the dynamic flow and environmental control significantly enhanced epidermal differentiation, dermo-epidermal junction integrity, and barrier function [79].

**Fig. 6:**
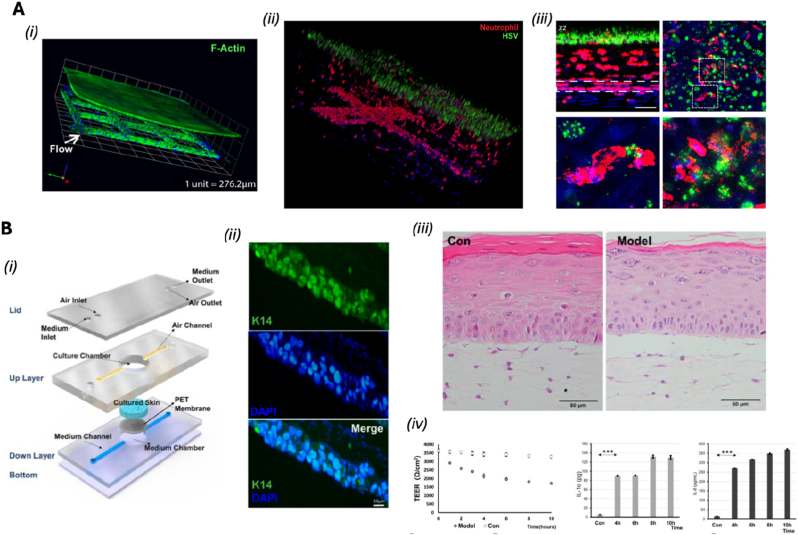
SoC platforms for investigating skin infections. **A(i):** 3D confocal image reconstruction showing the cytoskeletal structure of a bioengineered epidermis and dermis supported by an endothelialized microvascular network (white arrow indicates microfluidic flow direction). Cytoskeletal F-actin is shown in green, and cell nuclei are labeled with DAPI (blue); **A(ii):** 3D confocal image reconstruction illustrating the distribution of infiltrating neutrophils (red, CD15) and HSV-infected epidermis (green) in the SoC. Neutrophils were introduced into the vascular network 1 h after the epidermis was exposed to HSV-1 K26 (106 PFU). Scale bar: 100 μm; **A(iii):** Top left: cross-sectional view showing neutrophils (red) migrating into the dermal layer toward the HSV-infected (green) epidermis. Dashed lines indicate vessel boundaries. Top right: horizontal view of neutrophil infiltration within the infected epidermis. Bottom panels: enlarged images of the highlighted regions, demonstrating close interactions between neutrophils and infected keratinocytes. Reprinted with permission from Ref. [81] © 2022 Nature Communications. **B(i):** Schematic of the IC-SoC device, highlighting its four key layers and membrane structure; **B(ii):** IC-SoC model reveals K14 expression (marker for keratinocyte differentiation) after seven days of ALI culture. Scale bar: 10 μm; **B(iii):** H&E-stained cross-sections comparing control (Con) and the experimental model; **B(iv):** TEER measurements of control (Con) and experimental model exposed to SLS + P. acnes at various time points post-stimulation. Cytokine release profiles for IL-1α and IL-8 are shown in response to the stimuli. Reprinted with permission from © 2022 Frontiers. (For interpretation of the references to color in this figure legend, the reader is referred to the Web version of this article.)

To explore immune responses, Michielon et al. developed an endothelialized SoC system combining a modular multiwell adapter with a commercial 6-well plate. The platform featured perfused dermal equivalents and reconstructed human skin (RhS) cultured on Transwell inserts. EC seeded beneath the dermis mimicked the vasculature, while myeloid dendritic cell precursors introduced under flow migrated in response to nickel sulfate exposure, modeling immune activation in allergic contact dermatitis [80]. Whereas Sun et al. developed a gravity-driven, microfluidic-based, full-thickness human SoC platform with an endothelialized, perfusable microvascular network (Fig. 6Ai). In particular, the microvascular network made by EC was inserted in a fibroblast-supplemented collagen matrix and placed between two plexiglass layers. The top plexiglass piece showed a well with two injection ports for collagen infusion, two ports for the inlet and outlet to perfuse EC and medium, and one open well for the culture of keratinocytes on top, resembling a protective barrier. This platform was used for simulating HSV infection (Fig. 6Aii-iii), studying immune responses, and evaluating antiviral drug effectiveness. HSV infection in the SoC demonstrated key morphological and pathophysiological characteristics like genital herpes in humans, including the production of the proinflammatory cytokine IL-8, which led to rapid neutrophil trans-endothelial migration. Notably, perfusion with the antiviral drug Acyclovir successfully inhibited HSV infection in a dose-dependent and time-sensitive manner [81].

Beyond its barrier and vascular functions, the skin is a highly innervated organ, integrating sensory neurons that mediate touch, temperature, and pain [143,146]. To address the need for better *in vitro* systems to study the interaction between keratinocytes and sensory neurons, Ahn et al. developed a 3D innervated epidermal model on a microfluidic chip using a microfluidic chip that co-cultures sensory neurons and keratinocytes via a slope-based ALI technique. The device, fabricated from PDMS using soft lithography, featured dual hydrogel-filled channels—type I collagen for keratinocytes and collagen-laminin for neurons. This configuration supported epidermal stratification and neuronal integration without external ALI equipment. The model demonstrated enhanced epidermal differentiation, barrier function, and physiological innervation, and was further validated under hyperglycemic conditions to simulate diabetic skin pathology [82]. In parallel, SoC platforms are increasingly used to model inflammatory skin diseases and evaluate therapeutic responses. Quan et al. introduced an interface-controlled SoC (IC-SoC) system composed of cast PDMS layers, a porous PET membrane, and a transparent, clamped lid for real-time observation and reagent access. The system supported dynamic ALI culture of fibroblast-laden dermal equivalents and HaCaT keratinocytes, promoting the formation of structurally mature skin tissue. Upon exposure to Propionibacterium acnes and sodium lauryl sulfate (SLS), the model exhibited barrier disruption and elevated inflammatory cytokines (IL-1α, IL-8, PGE2). Treatment with polyphyllin H significantly restored barrier integrity and suppressed inflammation more effectively than dexamethasone, highlighting the platform's potential for drug screening and disease modeling (Fig. 6Bi-iv) [83].

Even if skin-related conditions are generally not life-threatening, their prevalence and the discomfort they cause underline the necessity for in-depth research. In this context, SoC platforms offer a powerful tool for replicating human skin physiology *in vitro*, enabling more accurate modeling of disease mechanisms, drug testing, and personalized therapeutic approaches without the ethical and translational limitations of animal models. Their capacity to more accurately replicate physiological conditions, lessen dependency on animal models, and facilitate personalized medicine strategies are some of their key benefits. There are still issues, though, like the complexity of the fabrication, the expense, the lack of platform standardization, and the requirement for additional validation to guarantee clinical relevance [144]. SoC systems are predicted to become a vital tool in drug development, disease modelling, and dermatological research as these limitations are gradually overcome, advancing the development of more potent and patient-specific treatments [143].

### 2.9 Joint-on-chip

The development of joint-on-chip (JoC) platforms offers a novel approach to modelling human joints. These systems aim to improve our understanding of the pathogenesis of musculoskeletal diseases and to facilitate treatment testing, in response to the growing demand for models that more closely replicate human pathophysiology. These platforms are based on microfluidics and combine multiple joint-specific cell types within controlled microenvironments. This setup enables complex cellular interactions under dynamic flow conditions, including mechanical and chemical stimulation that closely mimic *in vivo* joint physiology [[147], [148], [149]].

Heidenberger et al. developed a JoC model based on equine cells to study the fluid flow influences in joint inflammation. In this study, chondrocyte and synoviocyte organoids encapsulated in fibrin hydrogels were used to replicate the synovial-cartilage interface under dynamic flow conditions. Gravitational tilting was used at 4° and 1 Hz to simulate flow forces that are similar to those found in the joint and induce cyclic perfusion. This model allowed for the analysis of mechanical stimuli and the study of the affection of the expression of inflammatory and extracellular matrix remodeling genes, especially in response to the inflammatory factor IL-1β. It was observed that cyclic flow modulates the expression of pro-inflammatory cytokines associated to inflammation and tissue remodeling, such as IL-6 and MMP-13, demonstrating that mechanical stimuli contribute to the pathological microenvironment of osteoarthritis (OA) [150].

Mainardi et al. developed an Osteochondral Unit-on-Chip (OCU-on-Chip) platform to model the mechanical stress linked to the pathophysiology of OA, replicating tissue-specific compression across the cartilage–bone interface in a controlled, stress-dependent approach. The microfluidic device incorporates a unique capillary burst valve (CBV) design that separates the two key layers (mineralized subchondral bone and hyaline cartilage) allowing compartment-specific mechanical stimulation under controlled compression. The system successfully recreated *in vivo* stress gradients by applying a hyperphysiological 30 % compression to the cartilage layer while preserving physiological deformation levels in the mineralized layer. This loading led to several OA-like features, such as increased calcium crystal release, hypermineralization, and upregulation of mechanotransduction genes including FOS, C-JUN, and MMP13. In addition, the mineralized layer supported distinct chondrocyte subpopulations linked to OA, along with gene expression changes, including a reduction in ribosomal gene activity, as shown by single-cell RNA sequencing (scRNA-seq). The model exhibited increased levels of IL-8, IL-6, and pro-MMP13, reinforcing its usefulness in exploring how variations in subchondral bone stiffness affect the inflammatory behaviour of chondrocytes under mechanical load. Altogether, these results highlight the value of the OCU-on-Chip as a robust platform for studying bone–cartilage interactions under stress, shedding light on the biological processes that contribute to osteoarthritis and its associated inflammation [151].

Zhao et al. developed a cartilage-on-chip (CoC) system to replicate key aspects of OA and to test potential treatments in a setting that closely mimics the native tissue environment. The platform integrated primary mouse chondrocytes within a GelMA hydrogel, cultured inside a microfluidic device that supported the chondrogenic phenotype and enabled a steady flow of nutrients. When exposed to interleukin-1β (IL-1β) over three days, the system began to show classical signs of OA, including an increase in apoptotic cells, a loss of glycosaminoglycans, and heightened expression of catabolic markers like MMP13 and ADAMTS5. The CoC model maintained the expression of key cartilage-related genes (Col II, ACAN, and SOX9) more effectively than standard 2D cultures or basic microfluidic controls, as confirmed through Western blotting, RT-qPCR, and flow cytometry. The platform was also used to test the effectiveness of both chemical and biological treatments, which reduced OA-like changes in a way that closely were consistent with the results seen in live models. These findings highlight the CoC system as a promising and versatile tool for OA drug screening, offering a valuable combination of 3D tissue structure, dynamic fluid flow, and disease-specific modelling in a single platform [152].

In summary, JoC systems offer a more physiologically relevant model of the human joint, closely replicating the cellular and molecular mechanisms involved in inflammation and tissue degradation. By supporting the co-culture of multiple cell types under dynamic flow, these platforms enable controlled simulation of inflammation, enabling for the testing of personalized therapeutic approaches using patient-derived samples. However, these platforms still have limitations such as the complexity of device fabrication, the need to standardize co-culture protocols, and the absence of real joint mechanical forces. However, recent advances establish the JoC as a promising platform for studying joint pathophysiology and screening treatments, potentially accelerating the transition of therapies from the laboratory to the patient [147,149,153].

### 2.10 Cancer-on-chip

Cancer-on-chip (CoC) technology has emerged as an innovative approach for simulating complex tumor microenvironments (TME) with a level of physiological relevance that surpasses conventional models [4,154]. Notably, these microfluidic models necessitate only a minimal number of cells, making them advantageous compared to patient biopsy samples [155]. This flexibility has made CoC particularly useful for investigating phenomena such as metastasis, organotropism (the tendency of tumors to spread to specific organs), and drug resistance [156]. The TME is a complex 3D environment consisting of a remodeled and vascularized ECM that contains cancer cells and various non-cancerous cell types [157]. The non-tumoral cell types present in the TME include EC, cancer associated fibroblasts (CAFs), immune cells (lymphocytes and macrophages), and other tissue-resident cells. Additionally, TME comprises a non-cellular segment that consists of ECM elements like fibronectin, collagen, hyaluronan and laminin, and a complex array of soluble factors such as cytokines, chemokines, enzymes, growth factors and extracellular vesicles. These cellular and non-cellular elements of the TME create the intricate signaling network that promotes tumor development and sustains its progression [154]. To model pancreatic ductal adenocarcinoma (PDAC), Haque et al. developed a CoC system incorporating patient-derived organoids (PDOs) co-cultured with pancreatic stellate cells and macrophages. The PDMS-based chip featured dual chambers separated by a porous membrane, with continuous perfusion mimicking interstitial flow. The multicellular setup extended PDO viability and revealed that targeting stromal components enhanced chemotherapy efficacy, underscoring the critical role of the stroma in PDAC progression [84]. In addition, Truong et al. designed a spatially organized breast cancer CoC model to study tumor–stroma crosstalk. Their concentric PDMS device featured a central tumor chamber surrounded by a stromal region, separated by trapezoidal micro-posts that permitted molecular diffusion. Co-culture of SUM-159 breast cancer cells with patient-derived CAFs demonstrated that CAFs significantly promoted tumor cell migration and invasion, highlighting their role in metastatic dissemination [85].

**Fig. 7:**
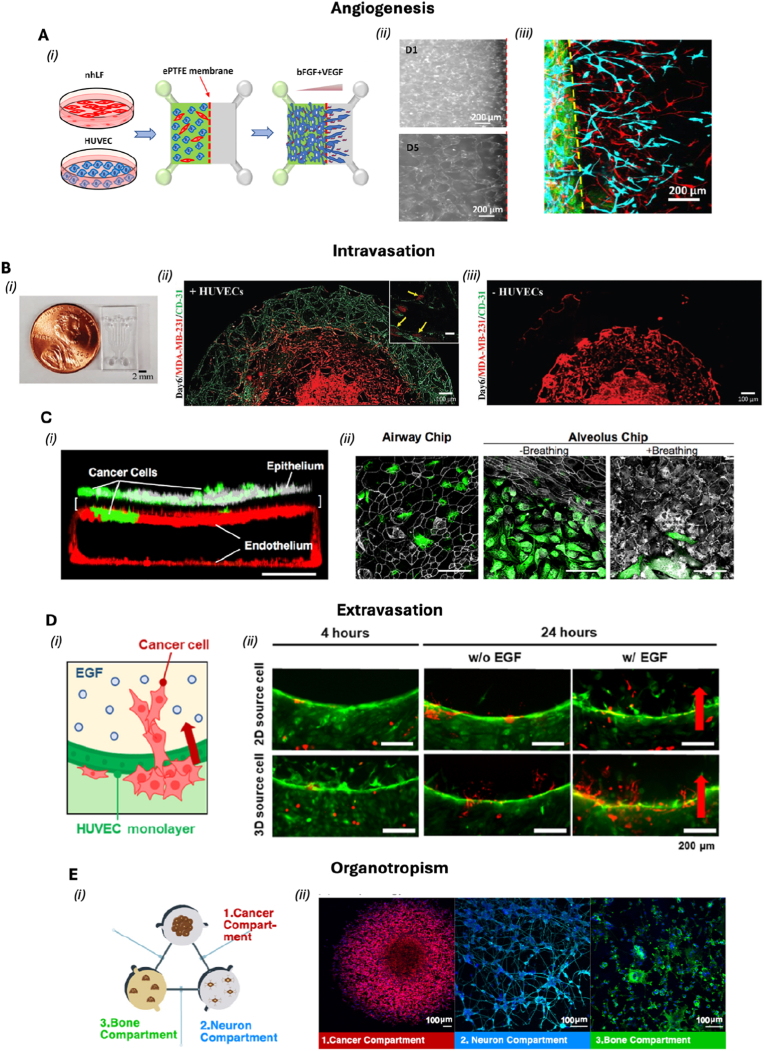
Examples of CoC models designed to study different steps of metastasis. **(Ai)** Workflow showing co-culture of endothelial cells (HUVECs) and fibroblasts (NHLFs) prior to integration into a microfluidic platform. Angiogenesis was induced using bFGF and VEGF gradients. **(Aii)** Time-lapse images showing progressive vascular network formation by day 5. **(Aiii)** Angiogenesis visualized across an ePTFE membrane (yellow dotted line); BFP-HUVECs (cyan), RFP-fibroblasts (red). Reprinted with permission from Ref. [89] © 2020 Elsevier Ltd. **(Bi)** Diagram of a three-level microfluidic platform; **(Bii)** Fluorescence images showing MDA-MB-231 cancer cells (red) invading the vascular network by day 6 (yellow arrows); **(Biii)** In the absence of HUVECs, cancer cells remained confined to the stromal region. Reprinted with permission from Ref. [90] © 2018 WILEY-VCH Verlag GmbH & Co. KGaA, Weinheim. **(Ci)** Confocal cross-section of an alveolus-on-chip model showing GFP-labeled lung cancer cells (green), epithelial tight junctions (ZO-1, white), and EC (VE-cadherin, red).**(Cii)** Comparison of cancer cell growth in airway vs. alveolus chips, with or without simulated breathing motion. (scale bar: 50 μm). Reprinted with permission from Ref. [158] © 2017 Cell Press. **D(i):** Diagram showing the chip setup with HUVECs, cancer cells, and EGF; (**Dii):** Images illustrating the extravasation of cancer cells (red) originating from 2D or 3D cultures through the HUVECs layer (green) over 28 h in the presence or absence of EGF (scale bar: 200 μm) Reprinted with permission from Ref. [91] © 2024 Bioactive Materials. (**Ei):** Schematic of the microfluidic device; **E(ii):** Micrographs representing compartments for cancer cells, neurons, and bone. Scale bar: 100 μmn [92] © 2022 Materials Today Bio. (For interpretation of the references to color in this figure legend, the reader is referred to the Web version of this article.)

To investigate how tumor cells stimulate angiogenesis, various microfluidic devices using hydrogels have been developed. For example, Surendran et al. developed a magnetically aligned microfluidic system to study tumor-induced angiogenesis. Breast cancer spheroids embedded in collagen were co-cultured with EC on a Transwell membrane. The system preserved EC polarity and allowed precise temporal control of co-culture assembly. Tumor cells induced EC proliferation and junctional disruption, effects that were reversed by targeting VEGF and Notch signaling pathways [86]. In another recent study, Bai et al. engineered a microfluidic model to visualize angiogenic sprouting across a porous membrane. HUVECs and fibroblasts co-cultured in a fibrin matrix self-organized into vascular networks. Upon exposure to a VEGF and bFGF gradient, endothelial sprouts extended into adjacent gel regions, forming perfusable neo-vessels. This platform enabled real-time observation of capillary morphogenesis and provided a robust system for evaluating anti-angiogenic therapies. (Fig. 7Ai-iii), demonstrating the platform's effectiveness in recreating angiogenesis *in vitro* [89].

CoC models are becoming also more widely employed to investigate how the TME influences the different stages of cancer metastasis, a process responsible for most cancer-related fatalities. Despite its critical role, the cellular and molecular mechanisms driving metastasis are still not fully understood [154]. Nagaraju et al. developed a 3D microfluidic CoC platform composed of concentric, hydrogel-based layers that mimic the spatial organization of the tumor microenvironment (TME). The PDMS device featured three circular regions representing tumor, stroma, and vasculature, separated by microposts that allowed molecular diffusion and cell migration. Highly metastatic MDA-MB-231 or less invasive MCF7 breast cancer cells were embedded in collagen in the tumor region, while HUVECs in fibrin gel formed the vascular compartment. The model revealed that spontaneous vascular formation facilitated tumor cell invasion and intravasation, accompanied by vessel narrowing and increased permeability—hallmarks of early metastatic progression (Fig. 7Bi-iii). [90]. In another study, Hassell et al. introduced an orthotopic lung CoC p model that mimicked the human lung microenvironment. The dual-channel PDMS device featured an upper airway channel and a lower vascular channel, separated by a semipermeable membrane. Human lung epithelial cells and NSCLC tumor cells were cultured on the apical side, while lung microvascular EC lined the vascular compartment. Cyclic mechanical strain simulated breathing motions. Remarkably, mechanical forces suppressed tumor growth and invasion but also promoted the emergence of drug-resistant cancer cell populations in response to third-generation tyrosine kinase inhibitors (TKIs), reflecting clinical patterns of resistance (Fig. 7Cii) [158].

To investigate metastatic extravasation, Lee et al. developed a vascularized microfluidic model incorporating 3D ovarian cancer spheroids generated on hydrophobic-coated plates. The PDMS chip, fabricated using SLA 3D printing, featured a collagen gel region embedded with normal human lung fibroblasts (NHLFs) and surrounded by endothelial-lined media channels. SKOV3 spheroids introduced into the endothelial lumen exhibited enhanced extravasation in response to EGF and fibroblast-derived cytokines. The model demonstrated that spheroid-derived cancer cells displayed greater metastatic potential than 2D-cultured cells, validating its utility for anti-metastatic drug testing (Fig. 7Di-ii) [91]. To capture the complexity of organ-specific metastasis, Conceição et al. designed a multi-compartment microfluidic platform simulating breast cancer metastasis to bone in the presence of neuronal signaling. The PDMS-based device, fabricated via 3D printing, included compartments for breast cancer spheroids (MDA-MB-231-BoM 1833), bovine bone slices, and human sympathetic neurons (SH-SY5Y), interconnected by diffusion channels. The study revealed that paracrine signaling from neurons and osteoclasts enhanced tumor aggressiveness, as evidenced by elevated pro-inflammatory cytokine levels, highlighting the role of the microenvironment in metastatic progression (Fig. 7Ei-ii) [92].

The TME also comprises various immune cells, including tumor-associated macrophages (TAMs), T cells, natural killer (NK) cells, and DCs. These immune cells are pivotal, either supporting tumor growth by inhibiting anti-tumor responses or contrasting the tumor through immune monitoring. Their actions are influenced by cues from the tumor and nearby stromal cells, resulting in complex and dynamic interactions [154]. Aung et al. introduced a multicellular CoC platform that integrates cancer cells, monocytes, andECs within a perfusable microfluidic device. Using a bilayer GelMA hydrogel and photopatterning, breast cancer cells (MCF7 or MDA-MB-231) and THP-1 monocytes were encapsulated in the inner layer, while HUVECs formed an outer endothelial layer. T cells introduced via perfusion migrated into the construct, with hypoxic tumor spheroids and monocytes enhancing T-cell recruitment. This model enabled the study of immune cell infiltration and chemokine-driven migration in a controlled 3D environment [93].

To evaluate chimeric antigen receptor (CAR)-T cell therapy, Maulana et al. developed a two-layer CoC system featuring tumor aggregates or PDOs embedded in a dextran-based hydrogel. The tumor chambers were separated from a perfused medium channel by a semipermeable, endothelialized PET membrane. The platform supported CAR-T cell migration, tumor infiltration, and cytotoxicity over a one-week period, offering a robust tool for assessing CAR-T efficacy and cytokine release in solid tumor contexts [95]. Beyond T cells, monocytes are gaining attention as therapeutic targets. Boussommier-Calleja et al. used a vascularized CoC model to study monocyte–tumor interactions. A fibrin-based microvascular network was established using HUVECs and fibroblasts. Monocytes introduced via perfusion reduced cancer _(MDA-MB-231 or the human melanoma cell line MDA-MB-435) cell extravasation, although this effect diminished once monocytes differentiated into macrophage-like cells [94].

To explore drug delivery in a vascularized 3D chip-on-chip (CoC) system, Haase et al. developed a microfluidic platform to grow tumor spheroids (derived from SKOV3 ovarian and A549 lung cancer cells) within a functional microvascular network. Skov3 and A549 spheroids were cultured for seven days before being seeded with HUVECs and human fibroblasts in the device, embedded within a fibrin hydrogel. Paclitaxel treatment led to notable remodeling of the TME and tumor-associated vasculature in the device. It also caused significant damage to EC, alongside its cytotoxic effects on cancer cells. Interestingly, the CoC model revealed that Paclitaxel's impact on tumor cells was less pronounced compared to its effects on simpler cancer cell spheroid models. This finding highlights the critical role of vascular and TME representation in creating more accurate platforms for evaluating drug efficacy [96].

These advanced CoC systems underscore the importance of modeling the full metastatic cascade—from intravasation to colonization—within organ-specific contexts. By integrating vascular, stromal, and neural components, they offer a physiologically relevant platform in evaluating drug transport, vascular toxicity, and therapeutic efficacy within a physiologically relevant TME. By capturing the spatial and cellular complexity of solid tumors, these systems offer a more predictive alternative to conventional *in vitro* models for preclinical drug screening.

### 2.11 Other organs

Currently, the organs that show more advances are focused on organs like the heart, liver, lungs, and blood vessels. Although mimic an organ is difficult depending on the complexity of the ones discussed above are relatively easier to model and have immediate applications in drug development and disease modeling. Conversely, there is other organs that are more difficult to mimic because of the cell environment, ensuring the diversity of cell types and optimizing culture conditions to support the growth and function of these cells are critical. For example, the spleen and lymph nodes have a complex immune cell environment that is difficult to replicate on a chip. Developing OoC that accurately replicate the complex structure and function of these organs is still in its early stages. For other organs as placenta and spleen the problem is ensuring proper vascularization (blood supply) and innervation (nerve supply) which are essential for the functionality but replicating these features *in vitro* is challenging but necessary to maintain the physiological relevance of the models. Models of OoC for the lung, liver, kidney, and intestine are essential for understanding how NPs behave in absorption, distribution, metabolism, and excretion (ADME) processes. For example, LoC allows the analysis of NP hepatotoxicity, while KoC can predict nephrotoxicity induced by nanomaterials.

The filtration function of the spleen-on-a-chip technology was mimicking by using instruments to simulate the hydrodynamic and geometric conditions of the spleen, enabling precise investigation of cellular interactions and retention processes [13,159]. In contrast with previous studies that considered the rigid wall assumptions, Li et al., propose two innovative models—passive and active deformable interendothelial slits (IES)—to better understand the filtration process. Using a microfluidic chip designed to mimic the IES and to observe the Red Blood Cells (RBC) dynamics under controlled conditions. The dimensions of the slits range from 0.5 to 1.2 μm, consistent with reported data on the sizes of IES in the spleen. The passive model utilizes a worm-like string representation to depict the deformation of EC as RBC traverse the slits and the active model incorporates pressure regulation, where the deformation of the IES is influenced by the local pressure surrounding the slit, facilitating the passage of RBC. Studying the dynamics of malaria-infected RBC, it was found that these cells exhibit increased stiffness and reduced deformability, complicating their passage through IES. The simulations suggest that the active model can alleviate congestion in the spleen caused by diseased RBC. However, both passive and active mechanisms work together to optimize the clearance of altered RBC [97].

Due to ethical concerns and physiological differences between human and animal placentas, Placenta-on-chip models offer a non-invasive alternative to *in vivo* studies. In a recent study, the development and validation of a second-trimester placenta OoC model (2TPLA-OoC) was used to investigate the effects of EDCs on placental function [102]. Six cell lines were seeding in interconnected microchannels, including placental vascular EC (PVEC), decidual cells, cytotrophoblasts (CTBs), syncytiotrophoblasts (STBs), placental stromal cells, and HUVECs. The 2TPLA-OoC model showed high cell viability (>90 %) and preserved the expected morphology and function of the different cell types. Several EDS including bisphenol A (BPA), bisphenol S (BPS), and polybrominated diphenyl ethers (PBDEs) at a concentration of 150 ng/mL^−1^ for 72 h, and a positive control of cigarette smoke extract. The Results shown that EDCs induced oxidative stress and localized inflammation in placental cells, but the organ demonstrated compensatory mechanisms that maintained placental function, including nutrient transport. However, in the case of BPA exposure a reduction in glutathione levels, indicating oxidative stress, and disrupted hormone production, particularly decreasing progesterone and β-hCG levels was observed [102].

**Fig. 8:**
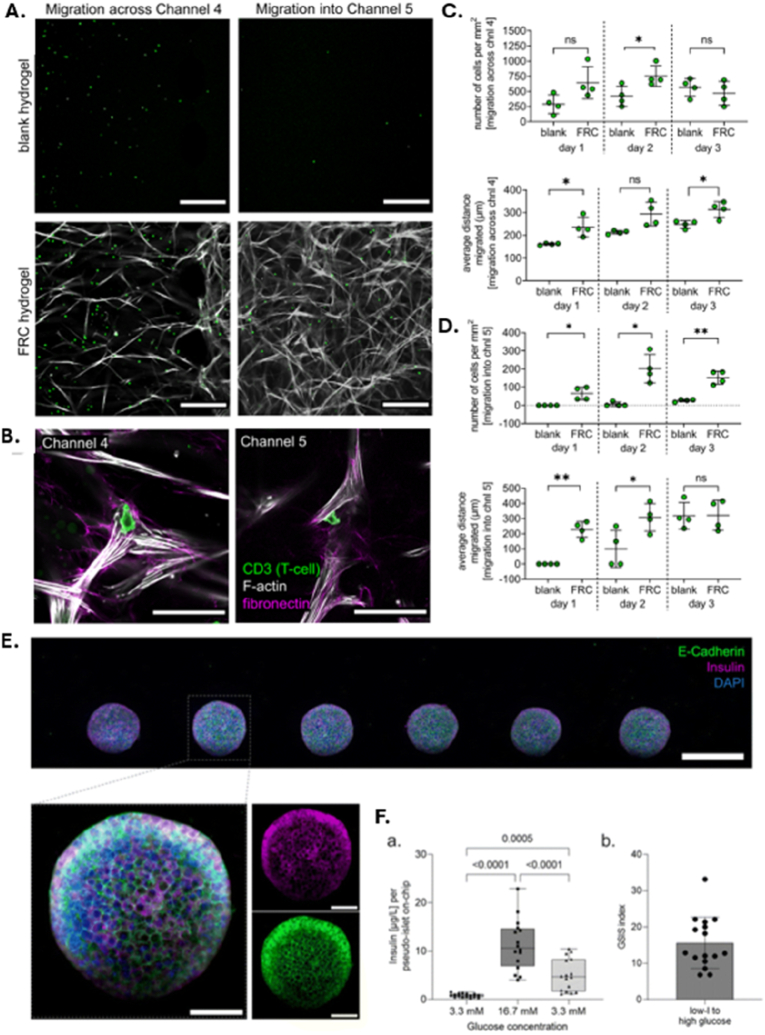
TNF-*α*-matured monocyte-derived dendritic cell migration toward lymph node stromal network. **A:** Representative image showing TNF-moDCs migrating from Channel 4 into Channel 5 toward either a blank or FRC-containing hydrogel on day 3. **B:** High-resolution image of TNF-moDCs (magenta) interacting with FRCs (green fibronectin, white F-actin) in Channel 5.**C–D:** Quantification of TNF-moDC migration across Channel 4 **(C)** and into Channel 5 **(D)** over 3 days (n = 5 devices; ∗p < 0.05, ∗∗p < 0.01). Reprinted with permission from Ref. [106] © 2024 Biofabrication published by IOP Publishing Ltd **E:** LSM-imaging of a pancreas-on-chip system stained for e-cadherin, insulin and DAPI displaying the six pseudo-islets in the tissue channel (tile scan; standard deviation projection of z-stack). Scale bar 200 μm (tile scan) and 50 μm (zoom-in on single pseudo-islet). **F:** (a) Glucose-stimulated insulin secretion of pseudo-islets on-chip (b) and corresponding GSIS index of high glucose normalized to first low glucose phase at 3.3 mM (one-way ANOVA, n = 16 chips). Reprinted with permission from Ref. [109] © 2024 Lab on chip published by the Royal Society of Chemistry. (For interpretation of the references to color in this figure legend, the reader is referred to the Web version of this article.)

In the lymph, immune cells interact making this model more difficult to mimic. There is an effort to mimic the spatial organization and interactions of B and T lymphocytes in a lymph-on-chip. To address this, Kwee et al. developed a microfluidic device focusing on the trafficking of dendritic cells (DCs) and T-cells [106]. The device is engineered to model the human lymph node paracortex and consists of six channels (separated by micropillars) that allow for the sequential injection of hydrogels containing FRCs and immune cells based on surface tension and spatial geometry. Specifically, Channel 5 is designed to model the paracortex, while Channel 4 simulates the lymph node tissue that DCs must migrate across from the subcapsular sinus. It was found that TNF-α matured monocyte-derived DCs and T-cells migrated significantly further toward the FRC network compared to a blank hydrogel due to the role of the FRCs in secreting various ECM proteins and chemokines which promote the migration of immune cell. Another finding was that the migration of these cells was not solely dependent on the CCR7-ligand chemokines, which are typically associated with lymph node function. Instead, a combination of other cytokines secreted by the FRCs, such as CCL2 and IL-6, appeared to enhance the migration process (Fig. 8A–D) [106].

Pancreas-on-chip (PoC) technology can be used in the study of diabetes and pancreatic diseases. As the work of Schlunder et al., discussed the endocrine function and metabolism of pancreatic tissues in diabetes diseases [104]. The system is based in thermoplastic and can self-guided trapping of pseudo-islets, which are formed from INS-1E embedded in an ECM-like hydrogel. To assess the metabolic activity of the tissues, the platform integrates luminescence-based optical sensors for non-invasive, real-time monitoring of oxygen levels. The system enables automated GSIS assays, demonstrating that the pseudo-islets maintain viability and functionality, with a GSIS index of 15.7 ± 7.1. Additionally, the integration of human pancreatic islet microtissues shown stable insulin secretion dynamics over a two-week culture period. The study also explores the effects of antidiabetic drugs, revealing that exendin-4 and tolbutamide significantly alter insulin secretion patterns in line with their mechanisms of action [109].

Accurately replicating the unique microenvironment of specific organs, such as the spleen, placenta, and stomach, is complex. This includes mimicking the organ's microarchitecture, cell-cell interactions, and ECM. Obtaining and maintaining the appropriate cell types that accurately represent the target organ's functionality is a significant challenge. This is particularly difficult for less common organs where primary cells may be scarce or difficult to culture.

### 2.12 Multi-OoC

Multi-OoC devices are engineered to mimic the interactions of various human organs or tissues, creating a more realistic and comprehensive model of human body physiology. Typically, multi-OoC consist of multiple compartments with different cell lines that replicate the tissue structure and function of individual organs and that are connected by channels or structures that facilitate fluid movement and physiological activities between the organs. Single-organ models have limitations in replicating the intricate interactions between different tissues and organs, underscoring the importance of multi-organ systems for simulating biological responses, including organ-organ relationships.

Lee et al. developed a platform designed to model and monitor cardiotoxicity induced by chemotherapy in breast cancer (BC) patients [160]. The platform integrates iPSC-derived cardiac tissues with BC tissues for non-invasive monitoring of biomarkers associated with cardiotoxicity and cancer progression using electrochemical immuno-aptasensors. Detecting chemotherapy-induced cardiotoxicity (CIC) are often invasive and only identify late-stage issues, the study aims to provide early detection by assessing the interaction between healthy and fibrotic cardiac tissues and BC tissues, which may exhibit different responses to chemotherapy. In conclusion, monitoring biomarkers like cardiac troponin T (Troponin T) and human epidermal growth factor receptor 2 (HER-2), which can indicate cardiac functionality and BC progression, respectively is very important. The findings suggest that personalized treatment strategies for breast cancer patients, particularly those with preexisting cardiac conditions can be assessed with the device [161].

Yu et al. constructed an intestinal-LoC system to study drug absorption and metabolism using Caco-2 and HT29-MTX-E12 for the intestinal model, and HepG2, HUVEC-T1, and THP-1 cells for the liver model [162]. After administering 4 mM acetaminophen (APAP) for 48 h, the two models were compared. The 2OC model demonstrated a higher survival rate of liver spheroids (83.94 ± 11.79 %) compared to those cultured in U-bottom plates (70.69 ± 26.97 %). Therefore, the 2OC model may provide a more favorable environment for liver cell survival under toxic conditions. In terms of liver function markers, the 2OC model showed a decrease in ALB levels after APAP treatment, indicating impaired liver function. Conversely, the U-bottom plate model demonstrated stronger signals for transporters like P-gp and multidrug resistance-associated protein 2 (MRP2) at the edges of liver spheroids, suggesting a more pronounced expression in that environment. In conclusion, the 2OC model appears to better simulate the physiological responses of liver cells to drug toxicity, while the U-bottom plate model may provide advantages in certain biomarker expressions and cell proliferation dynamics [163].

**Fig. 9:**
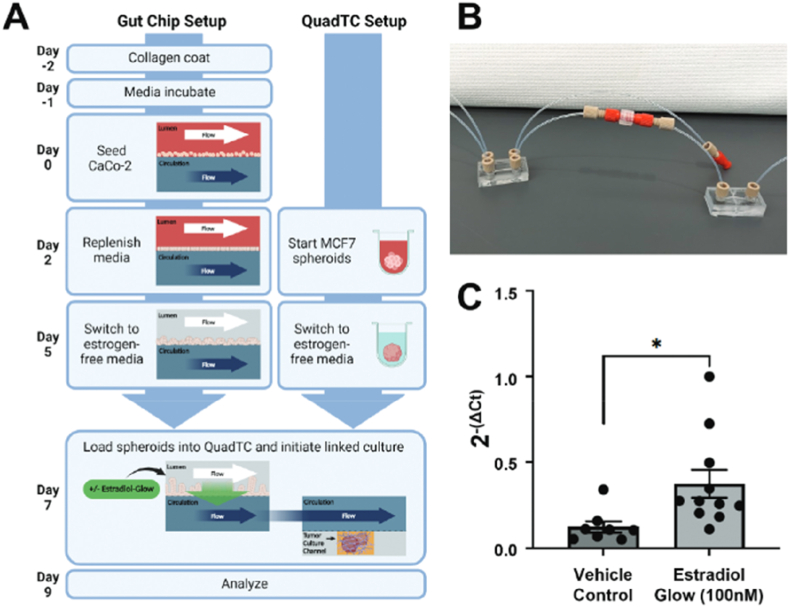
Multi-OoC models **A.** Demonstration of gut chip and QuadTC linked culture using estrogen as a model. A) Schematic overview of linked culture experiments. **B.** Example of simple tubing interconnects to enable linked culture using a gut chip (left) and SCTC (right). **C.** Following two days of linked culture, significantly increased expression of GREB1, an estrogen-responsive gene transcript, was detected in spheroids downstream of gut chips fed a fluorescently labeled estradiol, Estradiol Glow, ∗, p = 0.013, Welch's *t*-test, n = 8–11 over two experiments. Reprinted with permission from Ref. [163] © 2024 Advanced Science published by Wiley-VCH GmbH.

Brasino et al. created a novel OoC platform made entirely of polycarbonate (PC), designed to study the interactions between GoC microbiomes and distal tumors by connecting two different devices with tubings. The PC chips enable the culture of anaerobic bacteria alongside human GoC epithelial cells, allowing for the formation of polarized villus-like structures [163]. In this setup (Fig. 9), the GoC chip use Caco-2 cells, which are derived from human colon epithelial cells, to form polarized villus-like structures. The tumor chip, on the other hand, can support various cell types, including MCF7 breast cancer spheroids, which are used to model breast cancer. By connecting the circulatory compartments of the GoC chip to the tumor chip is possible investigate the absorption and circulation of metabolites, such as estrogen, and their regulatory effects on the tumor cells [163].

Fanniza et al., 2025 introduced the PEGASO platform, a dynamic, multi-organ-on-a-chip system that integrates both iPSC-derived cells and primary human cells. It models the gut–brain axis and includes modules for the gut, immune system, liver, blood–brain barrier, and brain. The platform was used to simulate the pharmacokinetics and efficacy of donepezil, a drug for Alzheimer's disease, demonstrating successful drug transport and biological response across the connected organ system [164].

### 2.13 Limitations and challenges of OoC

OoC technologies have emerged as powerful platforms for modeling human physiology and disease. However, their translational potential is often constrained by limitations in material properties, fabrication techniques, and biological integration [114]. PDMS remains the most widely used material in OoC fabrication due to its favorable properties, including biocompatibility, gas permeability, optical transparency, flexibility, and ease of prototyping. Despite these advantages, PDMS exhibits significant drawbacks, such as its hydrophobicity, poor mechanical mimicry of the ECM and its tendency to absorb small hydrophobic molecules, which can compromise drug testing accuracy and physiological relevance [165]. To address these limitations, alternative materials have been explored. Synthetic polymers such as polyurethane methacrylate (PUMA), thermosetting polyester (TPE)PC, polymethyl methacrylate (PMMA), and polystyrene (PS) offer improved mechanical properties and reduced molecular absorption. Recent innovations include the development of non-absorptive elastomers and transparent polyesters, often integrated with advanced microfabrication techniques like 3D printing and two-photon photolithography to enable complex, organ-specific geometries [166].

Natural biomaterials, including collagen and hyaluronic acid, are frequently employed to replicate the ECM due to their biocompatibility, biodegradability, and structural fidelity. Hybrid systems combining natural ECM components with synthetic scaffolds—such as collagen-polyethylene glycol (PEG) composites—enhance mechanical strength, control degradation rates, and support cellular adhesion and differentiation [167]. Additionally, decellularized tissue-derived matrices provide native ECM architecture while eliminating immunogenic cellular components, making them ideal for constructing physiologically relevant microenvironments [168]. Ultimately, material selection is guided by the specific aims of the study. By carefully combining different materials, CoC models that closely mimic the complexities and dynamics of the natural TME can be designed.

Traditional cell seeding methods in OoC platforms—typically involving manual pipetting or passive flow—often result in uneven cell distribution, low seeding efficiency, and incomplete confluency. These inconsistencies compromise the reproducibility and physiological accuracy of the models, particularly in applications requiring uniform barrier formation or precise tissue architecture. In microfluidic environments, where spatial constraints and fluid dynamics play critical roles, such limitations can significantly affect experimental outcomes. To address these challenges, recent advances have focused on the development of bioactive and stimuli-responsive materials that enhance cellular integration and functionality. These materials often include cell-adhesive ligands (e.g., RGD peptides) that promote integrin-mediated attachment, immobilized growth factors to direct lineage-specific differentiation or topographical features that influence cell orientation, polarity, and migration [169]. Such modifications improve initial cell attachment, promote uniform spreading, and support long-term viability and function, thereby enhancing the structural fidelity of the OoC model [170]. Other approach such as 3D bioprinting offers high-resolution deposition of cells and biomaterials, enabling the fabrication of tissue-like constructs with spatial fidelity [171].

Despite substantial progress, OoC technologies face several technical and scientific challenges. These include the need for standardized protocols, improved reproducibility, scalable manufacturing processes, and seamless integration with real-time analytical sensors. Moreover, the inherent complexity of human physiology, particularly in multi-organ systems, remains difficult to replicate within a single device. Ongoing research is focused on overcoming these barriers through the development of multifunctional materials, advanced fabrication techniques, and biologically relevant cell sources. These innovations are critical for transitioning OoC platforms from experimental tools to robust systems suitable for regulatory and industrial applications [10,172].

While iPSCs offer the advantage of patient-specific modeling and the potential for personalized medicine, their use introduces significant biological variability. Differences in genetic background, reprogramming efficiency, and differentiation protocols can lead to inconsistencies in cell behavior, tissue function, and experimental outcomes [173]. This inner variability complicates reproducibility and standardization, which are critical for translational applications [164]. Moreover, the microfluidic environment itself adds layers of complexity. Maintaining stable and physiologically relevant conditions, such as flow rates, nutrient gradients, and mechanical forces—can be technically demanding, especially when working with fragile or heterogeneous iPSC-derived tissues. These factors underscore the need for improved protocols, robust quality control, and scalable designs to ensure consistency across platforms and experiments [174].

## 3 OoC for NPs and advanced therapies screening

**Fig. 10:**
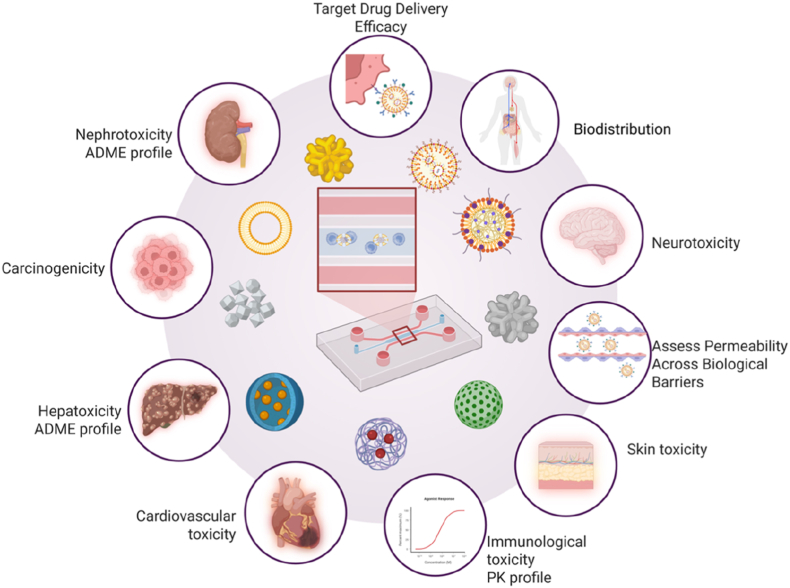
The diagram illustrates key parameters and toxicological endpoints assessed in drug development using OoC and related platforms. Created by Biorender.

The convergence of OoC technology and nanomedicine represents a transformative shift in preclinical research. NPs, engineered at the nanoscale, are increasingly employed in targeted drug delivery, molecular imaging, and diagnostic applications. However, despite their potential, the clinical translation of NPs remains limited. Traditional preclinical models, particularly animal studies, often fail to replicate the complexity of human physiology, leading to inaccurate predictions of NP behavior, efficacy, and toxicity [175]. OoC platforms offer a compelling alternative by mimicking the structural, mechanical, and biochemical features of human tissues within microfluidic environments as described in the above section. These systems enable dynamic modeling of human organ functions and provide a more predictive framework for evaluating nano-bio interactions. Importantly, OoCs facilitate the study of absorption, ADME processes, which are critical for understanding NP pharmacokinetics and pharmacodynamics, (Fig. 10).

As only a small fraction of NP-based formulations advances through clinical trials often due to unforeseen toxicities or off-target effects. The adoption of OoC platforms represents a critical step toward improving translational success and regulatory confidence on OoC technologies, by recapitulating key aspects of human tissue microenvironments and dynamic physiological conditions, offer a powerful complementary strategy to traditional models for assessing NP safety, efficacy, and mechanism of action as summarize in Table 2. Table 2 summarized the studies using NPs in OoC, described the type of system, type of NPs and main outcomes.

**Table 2:** Studies that evaluated NPs in OoC devices and main outcomes of the studies.

System	Organs and Cell types	NPs	Fabrication of NPs	OoC Study Function	Readouts	Ref.
Polymeric NPs						
Vessel-on-chip	HUVEC	200 nm polystyrene beads (PB) and discoidal polymeric nanoconstructs (DPN)	DPN were created by mixing a polymeric mixture and PB were commercially available by	Vascular permeability and NP transport under different hemodynamic conditions with intact or compromised endothelial barrier	Permeability coefficients under various conditions, such as the presence or absence of HUVEC and after treatment with permeabilizing agents like Mannitol and Lexiscan	[176]
Vessel-on-chip	HUVEC and pericytes	Titanium dioxide (TiO_2_) NPs and cationic polymer-coated silica NPs (PDMAEMA)	TiO_2_ NPs commercially available from SigmaAldrich and PDMAEMA layer by layer	Toxicity and vascular response to NP (vascular stability and interactions with pericytes)	Pericytes significantly stabilized the microvascular networks, preventing hyperplasia and maintaining vessel integrity under stress conditions such as nutrient starvation and exposure to toxic NPs	[177]
Vessel-on-chip	HUVEC	PS NPs with various size	Polystyrene NPs were purchased, which were functionalized with amine groups and labeled with rhodamine B. These NPs were available in four different sizes: 30 nm, 60 nm, 120 nm, and 250 nm, referred to as NP1, NP2, NP3, and NP4, respectively	NP permeability and adhesion	Permeability and binding assessments of the NPs across the En-on-a-chip and Dys-En-on-a-chip models	[178]
Gut-on-chip	Caco-2 (intestinal epithelium with mucus coating)	Gelatin methacrylate with acrylic acid (GelMA + AA), mucoadhesive and pH-sensitive), polyethylene glycol diacrylate (PEGDA)	NPs synthesized by polymerizing a PDMS Sylgard 184 A and B silicone prepolymer with a photoinitiator under UV light, incorporated by loading with Rhodamine B or by directly integrating FITC-dextran during polymerization	Evaluation of mucoadhesion, controlled release, and intestinal permeability (release profile and oral absorption)	Mucus adhesion of particles (microscopy, ImageJ), drug permeability (fluorescent molecules), pH sensitivity (controlled drug release)	[179]
Cancer-on-chip	Breast cancer cell line (MCF7)	Single-chain polymeric NPs (SCPNs)	Nine amphiphilic heterograft polymers synthesized using a post-functionalization procedure. The different polymers were dissolved in PBS, placed at 80 °C for 30 min and allowed to cool down for 4 h	Study of tumor penetration, cellular uptake, and NP stability in spheroids (tumor efficacy and transport within the tumor microenvironment)	All SCPNs exhibit strong ECM penetration, but their cellular uptake and stability were determined by the polymer's microstructure. Among them, glucose-based NPs achieve the highest uptake in spheroids, with charged NPs ranking second	[180]
Lymphatics-on-Chip	Human dermal lymphatic EC (LECs)	Poly(lactic-co-glycolic)-b-poly (ethylene glycol) (PLGA-b-PEG) NPs	Three-inlet flash nanoprecipitation device.	Investigation of endocytosis mechanisms, interstitial transport, and accumulation in lymphatic cells	Smaller NPs (30 and 50 nm) transported faster through the interstitial space but were captured and accumulated within the cytosol of LECs. Distinct endocytosis mechanisms for different sizes of NPs, with smaller ones relying on dynamin-mediated endocytosis, while larger ones utilized caveolin-dependent pathways	[181]
Mucus-on-chip	–	Chitosan SiO_2_ NPs, PLGA NPs, Mesoporous SiO_2_ NPs	CSNP were formulated via the ionotropic gelation method. Mesoporous and PLGA NP were synthesized using a modified oil-in-water emulsion method	NP permeation in mucus and impact of mucus structure on NP transport	Complex structure of the mucus significantly hinders the Permeation of NPs, permeation behavior of these NPs	[182]
Multi OoC (intestine-liver)	Caco-2 cells and HT29-MTX mucous-producing cells, while the liver was represented by HepG2/C3A cells	50 nm carboxylated PS NPs	commercially available from Polysciences Inc	Intestinal absorption and subsequent hepatotoxicity (ADME model, systemic toxicity)	Tthe presence of the GI tract tissue enhanced the sensitivity of the liver tissue to NP exposure, ingested NPs caused significant liver injury	[183]
Multi OoC (liver cancer-bone)	HepG2 cells (bone chamber is hydroxyapatite)	Chitosan (CS) NPs	CSNP were formulated via the ionotropic gelation method.	Metastatic model, cell migration, therapeutic efficacy with NPs	HepG2 cells proliferated and migrated significantly to the bone compartment, both free TQ and TQ-encapsulated NPs significantly reduced the number of metastatic HepG2 cells in the bone-like chamber, with the encapsulated form showing a more sustained release and effectiveness	[184]
Joint-on-chip	Human osteochondral tissue, synovium, and adipose tissue (chondrocytes, synoviocytes, adipocytes)	Drug-loaded polymeric NPs (naproxen, FGF18, IL-1Ra, SM04690, SOST)	Drug encapsulation into biodegradable polymeric carriers (PLGA)	Therapeutic efficacy and safety of nanodrugs for OA and RA in a multi-tissue joint model	Inflammation markers, ECM degradation, synovial hyperplasia, adipose inflammation (gene/protein expression, histology)	[185]
Cartilage-on-chip	Human chondrocytes cultured in articular cartilage-like microfluidic model	PAMAM dendrimers conjugated with anti-TNF-α antibodies	PAMAM dendrimer synthesis followed by covalent antibody conjugation (via EDC/NHS chemistry)	Anti-inflammatory activity and cytocompatibility of dendrimer-based nanocarriers under RA-like inflammatory conditions	Cytokine secretion (IL-6, TNF-α), chondrocyte viability, ECM integrity, gene expression	[186]
Neurovascular-on-chip	Human EC and neuronal cells	PLGA nanoparticles loaded with ibuprofen	Nanoprecipitation or emulsion–solvent evaporation using PLGA polymer and ibuprofen	Transport and anti-inflammatory response of ibuprofen NPs at neurovascular interface	NP transport, ibuprofen release kinetics, cytokine levels, barrier integrity (e.g., TEER, permeability assays)	[187]
**Metallic NPs**						
Vessel-on-chip	HUVEC	Resveratrol-gold NPs (RGNps)	The CGNps were synthesized by heating a gold solution and adding sodium citrate, which also caused a color change from yellow to red.	Evaluation of cytotoxicity, oxidative stress and cell morphology under flow (vascular biocompatibility and safety)	RGNps reduce the cytotoxicity associated with resveratrol, RGNps reduced intracellular oxidative stress by 57 %–82 %, outperforming both CGNps and free resveratrol, RGNps promoting a more elongated and spindle-like shape under dynamic flow conditions	[188]
Heart-on-chip	IPSC-CM, HUVEC, Human Cardiac Microvascular EC (HCMVEC)	Copper Oxide NPs (CuONPs), Silica NPs (SiO_2_ NPs), Gold NPs (AuNPs)	NPs commercially available from SigmaAldrich and nanoComposix	Analysis of cardiotoxicity, contractile damage, endothelial barrier function, ROS production, and intracellular calcium modulation	Cu NPs were found to be highly toxic, capable of translocating into cardiac tissue and causing electrical and contractile dysfunction through the generation of ROS(ROS), SiO_2_ NPs did not generate ROS but were associated with increased secretion of pro-inflammatory cytokines, which modulated intracellular calcium handling and the study observed that CuO NPs disrupted endothelial barrier function, while SiO_2_ and Au NPs maintained it, indicating a distinct mechanism of toxicity for CuO	[189]
Brain-on-chip	Human hAs, pericytes and EC	Gold nanorods (GNR) functionalized with PEG, angiopep-2 peptide (Ang2) and the D1 peptide	GNR-CTAB was synthetized by a seed-mediated growth method	Study of transport across the BBB, establishment of tight junctions, and penetration mechanisms	Development of a neurovascular system and the establishment of tight junctions within EC. GNR-PEG-Ang2/D1 NPs penetrated the BBB, a process aided by the Ang2 peptide	[190]
Brain-on-chip	Brain microvascular EC line (hCMEC/D3)	SWNTs-AuNPs-PPy/Tyr (Single-Walled Carbon Nanotubes-Au NPs Polypyrrole/Tyrosinase)	AuNPs commercially available from Zhongke Thunder	BBB permeability to drugs and NPs under dynamic flow	L-Dopa's permeability across the BBB enhanced as flow rate and duration increased, with its transport occurring via passive diffusion without compromising the BBB integrity	[191]
Liver-on-chip	Primary rat hepatocytes	Superparamagnetic iron oxide NPs (SPIONs)	SPIONs of iron oxide (Fe3O4) with a diameter of 10 nm commercially available from Sigma-Aldrich	Viability, liver function (urea, albumin) and biocompatibility	ALB secretion, urea production, cell viability (Live/Dead assay)	[192]
Liver-on-chip	Hepatocyte spheroids (3D culture derived from rat hepatocytes)	Copper sulfide NPs (CuSNPs)	Copper sulphide NPs (CuSNPs) synthesized using a direct method, dissolving BSA in water and adding copper nitrate under magnetic stirring, adjusting the pH with sodium hydroxide drop by drop until the solution turned purple, introducing sodium sulfide to induce a brick-red color change and heating the mixture at 90 °C for 30 min to form dark green	Hepatotoxicity, mitochondrial dysfunction, oxidative stress, transporter activity and viability	ALB secretion, urea production, mitochondrial membrane potential (MMP), ROS(ROS) levels, transporter activity (BSEP and MRP2), glycogen storage, and cell viability (ATP assay)	[193]
Gut-on-chip	Caco-2 (intestinal epithelial barrier)	Platinum dendrimer-encapsulated NPs (Pt DENs, G6-NH2 and G6-OH dendrimers)	Pt DENs synthesized by encapsulating platinum ions (Pt^2+^) in sixth generation PAMAM dendrimers (G6-NH2 and G6-OH)	Monitoring of hypoxia, intestinal epithelial barrier integrity, metabolism and transport studies in the intestine	Oxygen mapping (Amplex Red oxidation), oxygen gradient visualization (confocal microscopy), epithelial barrier integrity (immunofluorescence)	[194]
Gut-on-chip	Collembolans (F. candida)	Silver NPs (AgNPs, 50 nm, uncoated) and ionic Ag (AgNO_3_)	Silver NPs (AgNPs) with 50 nm commercially available from Aladdin Industrial Corporation (Hangzhou, China)	Assessment of environmental toxicity, bioaccumulation, reproductive effects and changes in the gut microbiota	Survival (adult mortality), reproduction (juveniles produced), GoC microbiota composition, GoC ARG profiles, and silver bioaccumulation	[195]
Gut-on-chip	Human ileal explants (male and female subjects)	AgNPs, sizes: 10, 20, 75, 110 nm	AgNPs commercially available from NanoComposix in BioPure formulations with spherical sizes of 10 nm, 20 nm, 75 nm and 110 nm	Study of penetration, structural dysfunction, inflammatory and gene expression responses	SEM and TEM imaging (morphology, structural integrity, NP penetration), cytokine levels (multiplex immunoassay), mRNA gene expression (cell junctions and permeability-related genes)	[196]
Lung-on-chip	Lung cancer cells (A549) and human fibroblasts	Human serum albumin (HSA) loaded with ZnO quantum dots	ZnO NPs encapsulated in HSA (60 nm) synthesized by mixing HSA with a ZnO solution under controlled pH and temperature, followed by gradual addition of the ZnO solution to HSA	Viability, pH monitoring, drug release and NP internalization	TEER monitoring, continuous pH monitoring, cell viability assays (trypan blue, live/dead), confocal imaging of NP internalization, pH-dependent quantum dot release	[197]
Lung-on-chip	A549 (adenocarcinomic human alveolar epithelial cells) and L929 (mouse fibroblasts)	L-cysteine-coated zinc oxide NPs (Cys-ZnO)	Zinc oxide NPs (ZnO NPs) synthesized by dispersing 30 mg of ZnO NPs in 30 mL of distilled water adjusted to pH 8.5, containing 15 mg of L-cysteine	Assessment of cytotoxicity, oxidative stress and mitochondrial alterations	Cellular morphology (coomassie brilliant blue), cell viability (MTT and neutral red uptake assays), cytotoxicity (LDH release), ROS and NO production, cytoskeletal integrity, mitochondrial health	[198]
Lung-on-chip	HUVEC and HPAEpiCs	Titaniumdioxide (TiO_2_) and zinc oxide (ZnO) NPs	ZnO-NPs produced from aqueous solutions of zinc oxide (200 mg/mL); TiO_2_-NPs floating in ultrapure water at the same concentration	Alveolar barrier dysfunction, junctional protein expression and cytotoxicity	Junction protein expression (E-cadherin and VE-cadherin), alveolar-capillary barrier permeability, ROS(ROS) production, cellular apoptosis (HPAEpiCs and HUVEC)	[199]
Kidney-on-chip	Renal cells	AuNPs coated with keratin labeled with 99m Tc	Keratin-coated gold NPs (Ker-AuNPs) synthesized using the Turkevich method, functionalized with keratin, and radiolabeled with 99mTc via DTPA, achieving a radiochemical purity of 90.7 %	Imaging (SPECT), viability and therapeutic efficacy (photothermal)	Cell viability, photothermal therapy (PTT) efficacy, NP characterization, SPECT imaging analysis	[200]
Kidney-on-chip	Renal tubular epithelial cells (NRK-52E), kidney tissue (UUO and DKD models)	SPIONs-decorated MSC-derived EVs overexpressing CHIP (SPION-EVs-CHIP)	MSCs genetically modified via lentiviral transfection to overexpress CHIP; EVs isolated and characterized (TEM, DLS, zeta potential, Western blot); SPIONs conjugated to EVs for magnetic targeting	Evaluation of targeted delivery, antifibrotic, and anti-inflammatory effects of CHIP-loaded EVs under fibrotic stimuli (TGF-β1, stiffness) and *in vivo* CKD models	Smad2/3 ubiquitination and degradation (Western blot), fibrosis markers (α-SMA, fibronectin, collagen I), cytokine levels, collagen deposition (histology), renal function, EV uptake (microscopy), biocompatibility (organ histology, liver enzymes)	[201]
Skin-on-chip	HaCaT cells	Au NPs	Functionalization with MUHEG, BSPP or ssDNAS	Study of dermal permeability and correlation with physicochemical properties	Correlation between the Stokes radius of the NPs and their permeability coefficients, demonstrating that the permeability coefficient can be estimated from the Stokes radius and vice versa	[202]
Placenta-on-chip	hTSCs and HUVEC	CuO NPs	Commercially available from Macklin	Assessment of placental cytotoxicity, inflammation, apoptosis and inhibition of trophoblast differentiation	CuO NPs hindered the differentiation of hTSCs into syncytiotrophoblasts, leading to reduced hormone secretion and impaired glucose transport. Exposure to CuO NPs disrupted autophagic flux and induced apoptosis in trophoblasts, while also triggering inflammatory responses within the placental barrier	[203]
Placenta-on-chip	BeWo b30 cells	TiO_2_-NPs	TiO_2_ NPs commercially available from SigmaAldrich	Oxidative stress, cell death and maternal immune response (macrophage adhesion)	Exposure to TiO2-NPs led to significant oxidative stress and cell apoptosis, maternal immune cells, specifically macrophages, showed increased adhesion to the trophoblastic layer following NP exposure, suggesting an inflammatory response	[204]
**Lipid NPs**						
Heart-on-chip	Fibroblasts into cardiomyocyte-like cells (iCMs)	DE-DOPE lipoplexes	The lipoplexes were prepared using the thin lipid film-hydration method	Therapeutic efficacy, miRNA transfection and reduction of cardiac fibrosis	miRcombo, which includes miR-1, miR-133, miR-208, and miR-499. This combination was delivered to human atrial CFB(AHCFs) using DE-DOPE lipoplexes. miRcomb reduced fibrotic traits, such as the number of α-SMA positive cells and collagen deposition	[205]
Liver-on-chip	HepG2 hepatocytes, human fibroblasts (HFF-1), and HUVEC	EVs from NK-92MI cells	NPs fabricated using a microfluidic electrospray approach with 2 % sodium alginate solution mixed with calcium chloride	Liver function and evaluation of EVs-based NPs	Cell viability (Live/Dead staining), ALB secretion, urea secretion	[206]
Cancer-on-chip	Human pancreatic cell lines, human alveolar type I epithelial cell line, HUVEC, bone marrow CD34^+^ hematopoietic stem and progenitor cells and human MSCs	Lipid NPs were loaded CRISPR-Cas9gRNA to delete the human IL30 (hIL30) gene and functionalized with anti-PSCA-Abs (Cas9hIL30-PSCA NPs)	Cationic lipid nanocomplexes coated with PEG were synthesized using a Dolomite Microfluidics device	Targeted gene editing, metastasis suppression and IL30 as a therapeutic target	Cas9gRNA-hIL30-releasing immunoliposomes blocked PC cell proliferation and regulated a wide range of metastasis driver genes, inhibiting their metastasization to the lungs and bone marrow	[207]
Placenta-on-chip	BeWo b30 cells	Chondroitin sulfate A (CSA)-conjugated liposomes	Microfluidic Dolomite five-input chip	Study of uptake under dynamic flow and syncytialization	Uptake of NPs by BeWo cells is significantly influenced by the conditions of syncytialization and shear stress. The uptake of CSA-conjugated liposomes was enhanced under dynamic conditions	[208]
**Other NPs**						
Kidney-on-chip	HK-2 cells (human proximal tubular epithelial cells)	Calcium oxalate monohydrate (COM) crystals, synthesized to simulate nephrolithiasis	COM NPs synthesized via electron microscopy under controlled physiological shear stress (0.2–0.8 dyn/cm^2^)	Modeling of nephrolithiasis, fibrosis and cellular stress	α-SMA (fibrosis marker), NGAL (acute kidney injury biomarker), fibronectin (fibrosis indicator), alkaline phosphatase (AP) activity (cell stress indicator)	[209]
Lung-on-chip	Simulated cell environment in a lung-on-a-chip device (no biological cells)	Simulated air-borne NPs	–	Simulation of transport and deposition of inhaled NPs	NP transport and adsorption kinetics, deposition patterns during exercise and breath-holding, simulated interactions based on Langmuir and Frumkin adsorption models, computational fluid dynamics	[210]
Liver-on-chip	Hepatocytes, liver microsomes, immortalized cell lines	Magnetic NPs, core-Shell NPs, biocompatible NPs	MNPs-NH2 prepared by immobilizing 5,10,15,20-tetrakis(2,3,4,5,6-pentafluorophenyl)porphyrin iron(II) (FeTPFP) covalently through incubation in diglyme with sonication and FeTSPP ionically in a methanol-acetate buffer, followed by separation, washing, and vacuum drying	Metabolite detection, enzymatic activity and real-time monitoring	Enzymatic activity measurement, drug metabolite detection, real-time monitoring, enzymatic activity measurement, enhanced catalytic efficiency, drug metabolite detection, real-time monitoring of physiological and metabolic processes	[211]
Vessel-on-chip	HUVEC, human lung FB	Atmospheric NPs (ANPs)	Collecting atmospheric particles from Wuhan city using a high-volume PM2.5 sampler	Study of the impact of atmospheric pollutants on vascular function	Plausible explanation for the link between air particle pollution and vascular diseases NP-induced vascular dysfunction	[212]

### 3.1 Biodistribution and permeability across biological membranes of NPs

Vessel-on-chip (VoC) models help predict the delivery and efficacy of NPs-based drugs. By simulating blood flow and vessel dynamics, researchers can assess how well NPs can reach their target tissues and avoid off-target effect as shown by Barbato et al., in a study on a double-channel microfluidic device designed to assess the transport of macromolecules and polymeric nanoconstructs across a vascular barrier, mimicking physiological and pathological conditions [132]. The selective permeation of therapeutic agents through diseased vasculature is crucial for effective treatment. Therefore, in the VoC under different flow conditions, it was utilized fluorescent microscopy to monitor the behavior of 250 kDa Dextran and 200 nm PS NPs. It was observed that the presence of EC reduced the permeability for larger particles, indicating a size-dependent selectivity of the vascular barrier. Furthermore, the study highlighted the importance of particle deformability, showing that soft DPN adhered more effectively to the vascular walls compared to rigid counterparts under pathological conditions [176].

**Fig. 11:**
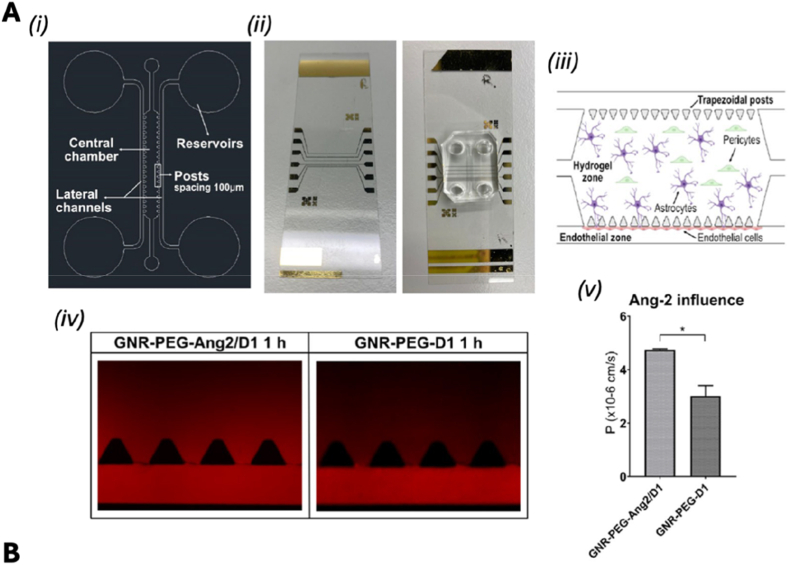
BoC Platforms for evaluating NPs behavior and therapeutic applications. **A(i):** an AutoCAD representation of the microfluidic layout, including electrode design and integration with the BBBoC system; **A(ii):** the fully assembled TEER-BBBoC device is shown, prepared for cell seeding and electrical resistance measurements; **A(iii):** a schematic representation of the arrangement of neurovascular cells within the BBBoC platform; **A(iv):** permeability studies of GNR-PEG-Ang2/D1 in the BBBoC are demonstrated through fluorescent imaging after a 1-h of incubation of GNR-PEG-Ang2/D1 and GNR-PEG-D1 in the ECs channel; **A(v):** statistical analysis revealed the permeability differences between GNR-PEG-Ang2/D1 and GNR-PEG-D1 over 1 h in the BBBoC (N = 3) (∗p < 0.05) Reprinted with permission from Ref. [190] © 2023 Journal of Nanobiotechnology on behalf of BMC.

Another of the global challenges for pharmaceutical companies, especially due to the low efficacy of most of the therapeutic compounds to cross the BBB, resides in neurological disorders like NDs [213]. Particularly, in Alzheimer's disease (AD), there are no effective treatments available and more than 200 therapeutic agents in clinical studies have failed [190]. NP penetration across the BBB is governed by multiple physicochemical parameters, including size, shape, surface charge, and functionalization. BBBoC systems, by recapitulating key features of the neurovascular unit, enable systematic investigation of these variables and their influence on NP behavior [159,160]. For example, Palma-Florez et al. proposed a BBBoC model incorporating human hAs, pericytes, and EC, equipped with a TEER measurement system (Fig. 11Ai-iii). developed a microfluidic BBBoC model incorporating human astrocytes, pericytes, and endothelial cells, integrated with TEER sensors for real-time barrier assessment. The platform featured a central hydrogel-filled chamber flanked by lateral channels, allowing for controlled cell seeding and media exchange. Gold electrodes fabricated via photolithography enabled spatially resolved TEER measurements. This innovative platform was used to evaluate the permeability of targeted GNRs designed for AD theranostics. The nanorods, synthetized by seed-mediated growth method, were functionalized with PEG, Angiotensin 2 (Ang2) for BBB penetration, and the D1 peptide to inhibit beta-amyloid fibrillation (GNR-PEG-Ang2/D1). The study demonstrated that these functionalized nanorods could cross the BBB, aided by the Ang2 peptide (Fig. 13Aiv-v), highlighting the potential of this platform for *in vitro* testing of NPs permeability [190].

**Fig. 12:**
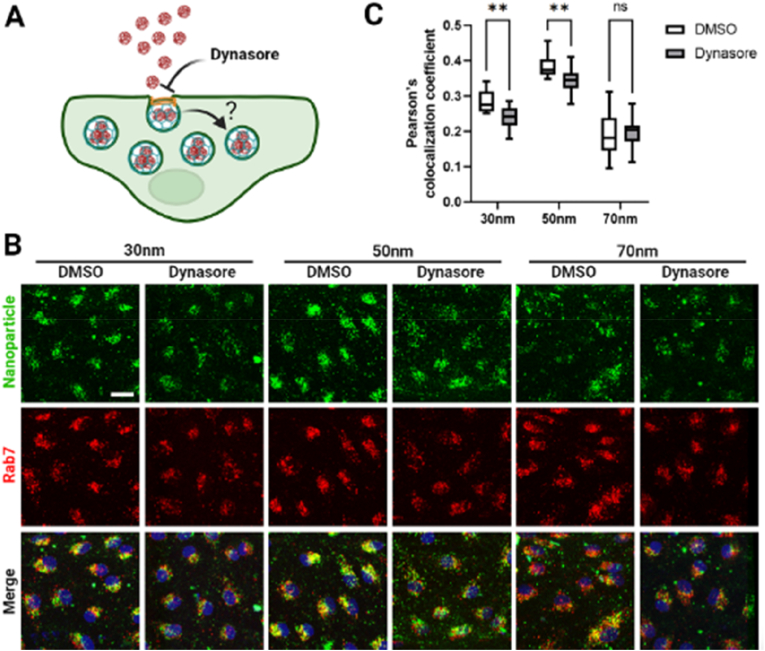
Smaller NPs accumulate in the Rab7-positive late-stage lymphatic endosomes, which is reversed by dynamin blockade. **(A)** Schematic of smaller NPs transports through lymphatics, which can be captured by dynamin-dependent cytosolic accumulation. **(B)** Fluorescence images of Rab7 (red)-stained LECs with NPs (green) show NPs colocalization with the late endosome marker Rab7. **(C)** Quantification of NPs and Rab7 colocalization with Pearson's colocalization coefficients (N = 8). Scale bar: 25 μm ∗∗p < 0.01 indicates statistical significance. “ns” indicates no significance. Reprinted with permission from Ref. [181] © 2024 American Chemical Society. (For interpretation of the references to color in this figure legend, the reader is referred to the Web version of this article.)

Lymph-on-chip systems are gaining attention for their potential in drug screening and understanding lymphatic transport mechanisms. As shown by Lu et al. that used a lymphatics-on-a-chip model to investigate the transport mechanisms of different-sized NPs [181]. The device aims to mimic a lymphatic vessel that can drain interstitial fluids to simulate the physiological environment near a subcutaneous injection site of NPs. NPs were loaded into the acellular channel by creating an interstitial fluid pressure gradient that drives NPs transport through the ECM toward the engineered lymph-on-chip. The PLGA-b-PEG NPs of sizes 30, 50, and 70 nm were injected in the device. The smaller NPs (30 and 50 nm) were transported through the interstitial space but accumulated within the cytosol of LECs, delaying their transport into the lymphatic lumen. Conversely, the larger 70 nm NPs which did not accumulate. To explore the mechanisms, various inhibitors were used to target different endocytosis pathways. It was found that inhibiting dynamin-mediated endocytosis with Dynasore enhanced the transport of smaller NPs into the lymphatic lumen, while caveolin inhibition with Nystatin decreased the transport of larger NPs. Additionally, smaller NPs were shown to accumulate in Rab7-positive late-stage endosomes, a process that could be reversed by dynamin blockade, suggesting Rab7 as a potential target for improving lymphatic delivery of smaller NPs (Fig. 12) [181].

**Fig. 13:**
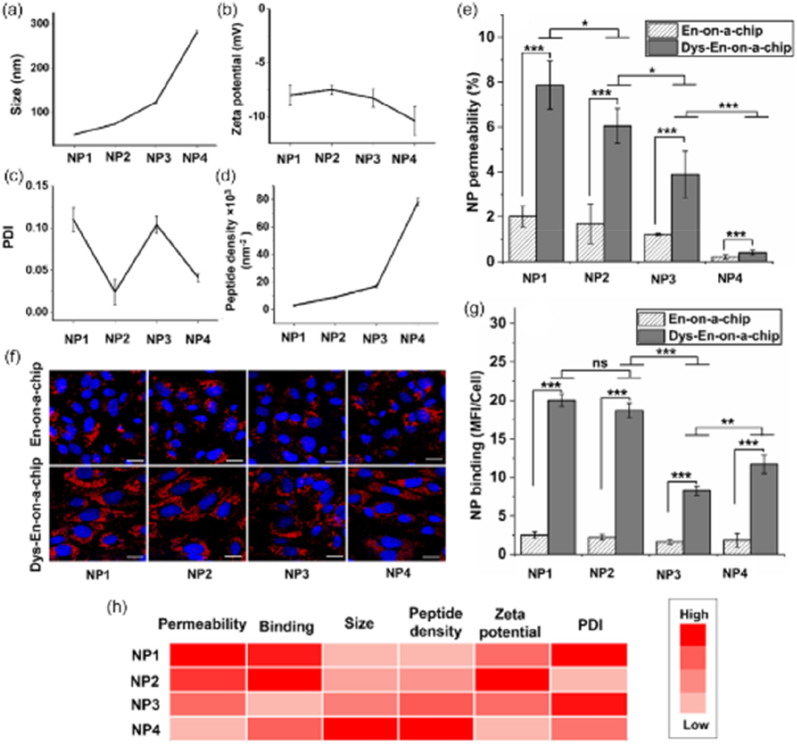
Characterization and screening of the NPs across En-on-a-chip and Dys-En-on-a-chip in this study. Characterization of the NPs for their **A.** size **B.** ζ-potential **C.** PDI, and **D.** peptide density. **E.** Permeability of the NPs across En-on-a-chip and Dys-En-on-a-chip. **F.** Representative images showing the binding of the NPs to En-on-a-chip and Dys-En-on-a-chip. Red and blue colors show NPs and cell nuclei, respectively (scale bar: 20 μm). **G.** Quantification of the binding results. **H.** Heat map summarizing the effect of NPs properties on their permeability and binding across Dys-En-on-a-chip. The results suggest that NPs size is the most dominant factor, and smaller NPs show higher permeability and binding. In this figure, data show mean SD of at least three independent experiments. ∗, ∗∗, and ∗∗∗ represent statistically significant differences with p-values of <0.05, <0.01, and <0.001, respectively. ns represents nonsignificant differences. Reprinted with permission from Ref. [178] © 2021 Advanced NanoBiomed Research published by Wiley-VCH Gmb. (For interpretation of the references to color in this figure legend, the reader is referred to the Web version of this article.)

SoC models can be highly effective for assessing critical parameters of NPs behavior, such as penetration, distribution, toxicity, and therapeutic efficacy, in a controlled and physiologically relevant environment [214], allowing to track NPs penetration through the stratum corneum into deeper layers, such as the epidermis and dermis, and to quantify (by means of fluorescence labeling) the spatial distribution of NPs within the skin-like system. In this regard, for instance, Fernandez-Carro et al. developed an innovative stratified epithelium-on-a-chip model using HaCaT cell. Stratified epithelia were cultured using the BE-Transflow devices from BEOnChip. The microdevice was composed by two culture wells, each connected to a 1 mm porous PC membrane and its respective microfluidic channel, allowing independent flow rates via any perfusion system. Reservoirs adjacent to the medium reservoirs, filled with PBS, prevented medium evaporation. HaCaT cells were seeded in the upper well of the microdevice, and four days post-seeding, the medium covering the cell monolayer was removed to establish an ALI. This platform enabled quantification of the permeation of different pharmaceutical formulations containing fluorescently labeled active compounds. Additionally, the system facilitated the correlation between the Stokes radius and the diffusion behavior of different molecules or NPs across the *in vitro* barrier, providing a robust tool for studying diffusion through stratified epithelial layers [202].

### 3.2 Targetability of NPs

CoC platforms also offer a flexible and robust system to test different therapeutic approaches, including NPs, designed to limit metastatic progression. Fieni et al. engineered a sophisticated CoC model replicating the dynamic environment of primary tumors and key metastatic sites such as the bone marrow and lungs. The microfluidic bioreactor employed, the commercial HUMIMIC Chip2, presented a 24-well compartment connected in a closed circuit to a 96-well compartment via microfluidic channels. The 24-well compartment contained a spheroid co-culture of human pancreatic cancer (PC) cells and HUVEC, linked through the channels to the 96-well compartment. This second compartment contained either a 3D spheroid co-culture of human alveolar type I epithelial cells (CI-hAELVi) and HUVEC, mimicking lung tissue, or a ceramic scaffold seeded with human BMSCs and CD34^+^ MSCs, representing the BM niche. Dynamic circulation of the culture medium between the two compartments was maintained by micropumps generating a pulsatile flow, regulated by a control unit to simulate shear forces characteristic of the bloodstream. This work highlighted the molecular pathways influenced by IL-30 in PC metastasis and assessed the potential of targeting IL-30 therapeutically at metastatic sites. To achieve this, they synthesized, using a commercial microfluidic device (Dolomite Microfluidics), cationic lipid NPs carrying CRISPR-Cas9gRNA to knock out the human IL-30 (hIL30) gene. These NPs were functionalized with anti-PSCA antibodies (Cas9hIL30-PSCA NPs), which recognize and bind to PSCA, a prostate-specific cell surface marker linked to glycosylphosphatidylinositol, and overexpressed in more than 80 % of prostate tumors. The efficacy of the NPs was evaluated both *in vivo* using xenograft models of lung metastasis and *in vitro* through the CoC system. The Cas9hIL30-PSCA NPs demonstrated excellent stability in circulation, fficient gene-editing capabilities, and a lack of off-target effects or organ toxicity. These NPs effectively inhibited PC cell proliferation, reduced IL-30 expression, and suppressed metastasis-associated genes. Additionally, they decreased the release of CXCL2/GROβ, which is linked to myeloid cell infiltration in metastatic sites, and DKK1, OPG, and IL-6, factors that promote endothelial network formation and tumor cell migration [207]. This study highlights the power of CoC systems in unraveling the complexities of tumor progression and evaluating cutting-edge therapeutic strategies to prevent metastasis.

In another study, the targabelity of VCAM-1 NPs were study. Bazban-Shotorbani et al. create the pathological conditions of blood vessels in microfluidics to study NPs behavior under flow conditions. Initially, a three-layered microchip was designed, two PDMS layers separated by a semipermeable membrane layer made of polyethylene terephthalate (PET). The cells were cultured on the membrane establishing a model of the endothelium-on-a-chip (En-on-a-chip). Following this, the En-on-a-chip was subjected to a proinflammatory cytokine, tumor necrosis factor-alpha (TNF-α), to induce a dysfunctional endothelium (Dys-En). The synthesis and characterization of VCAM-1-targeted PS NPs of varying sizes (30, 60, 120, and 250 nm referred to as NP1, NP2, NP3, and NP4, respectively) were made. These NPs were functionalized with a targeting peptide and PEGylated to enhance their stability and biocompatibility and they were introduced in both models. The NPs in the size range of 30–60 nm exhibited enhanced targeting to Dysfunctional Endothelium (Dys-En) suggesting that size is a critical factor in NP design for effective drug delivery as demonstrate in Fig. 13 [178].

Cheng et al. developed a targeted therapy for chronic kidney disease, focusing on renal interstitial fibrosis (RIF), a condition driven by Smad2/3-mediated inflammation and fibrosis [201]. They engineered mesenchymal stem cell-derived extracellular vesicles (MSC-EVs) to overexpress carboxyl terminus of the E3 ubiquitin ligase Hsp70 interacting protein (CHIP), a protein that promotes degradation of Smad2/3. To enhance kidney targeting, these EVs were decorated with superparamagnetic iron oxide nanoparticles (SPIONs) and guided using an external magnetic field. The modified EVs were successfully internalized by renal cells, leading to Smad2/3 degradation and reduced fibrosis markers. *In vivo* tests in rat models of diabetic kidney disease and ureteral obstruction showed improved kidney function, reduced fibrosis, and no significant toxicity [201].

### 3.3 Toxicity

VoC systems can provide a platform to evaluate the safety and potential toxicity of NPs. Fayazbakhsh et al. evaluated the antioxidant potential of RGNps in preventing oxidative stress in EC, particularly under hyperglycemic conditions [188]. The study utilized a microfluidic chip to simulate blood vessels and assess the antioxidant properties of RGNps compared to citrate AuNPs (CGNps) and free resveratrol on HUVEC. The study demonstrated that RGNps exhibited significantly higher cell viability (over 90 %) compared to free resveratrol, which had a cell viability of only 24–35 % 1. The DPPH assay results indicated that RGNps (20 nm) had a DPPH scavenging activity ranging from 38 to 86 %, outperforming CGNps and free resveratrol. Additionally, RGNps effectively reduced cellular oxidative stress by 57–82 %, highlighting their potential in alleviating oxidative stress and preventing EC disorders [188].

HoC devices were the foremost developed which allow for high-throughput testing and precise control over the dynamic interactions between NPs and cardiac cell. In addition, the cardiotoxicity of NPs has been extensively evaluated using HoC technology. These models enable the investigation of the effects of various NPs on cardiac contractility, electrical activity, and cellular survival by enabling real-time monitoring of cardiac responses to NPs. Lu et al. explored the detrimental effects of air pollution on cardiovascular tissues, particularly focusing on the mechanisms by which NPs contribute to cardiovascular diseases [189]. A novel microfluidic platform known as the Integrated Vasculature for Assessing Dynamic Events (InVADE) was developed. The InVADE platform is a gravity-assisted flow system that enable the continuous perfusion of cell culture media and NPs suspension. The device includes microholes in the vessel wall to allow for the exchange of NPs and macromolecules, enhancing intercellular communication and tissue functionality. In this study, human hiPSC-derived cardiac tissue with a vascular interface were used and the toxicity of CuO and SiO_2_ NPs, with AuNPs serving as a control, under conditions that mimic physiological blood flow was tested. By studying the penetration into cardiac tissue and the electrical and contractile dysfunction it was found that CuO NPs are particularly harmful. Due to the generation of ROS, which disrupt cardiac troponin T and lead to the secretion of biomarkers associated with cardiac injury, such as B-type natriuretic peptide (BNP) and Troponin I. In contrast, SiO_2_ NPs primarily trigger the release of pro-inflammatory cytokines and alter intracellular calcium influx without directly affecting cardiac contractility and Au NPs acted as a control, showing no adverse effects on the cardiovascular system [189].

In a study by Wu et al., the pathogenesis of SARS-CoV-2 was investigated using a 3D-printed HoC platform designed to mimic COVID-19-associated cardiac dysfunction [46]. The device incorporated 60 μm thermoplastic elastomer/quantum dot nanocomposite wires arranged in microwells, seeded with human iPSC-CMs and fibroblasts. This model successfully recapitulated key features of SARS-CoV-2 infection, including impaired contractile function, disrupted calcium handling, and increased apoptosis, with severity correlating with the multiplicity of infection. At higher viral loads, the tissues showed significant reductions in contractile force and calcium transients, accompanied by sarcomere disorganization and increased apoptosis. Notably, treatment with EVs derived from iPSCs mitigated these effects, reducing apoptosis and partially restoring contractile function. These findings highlight the cardioprotective potential of iPSC-derived EVs in the context of viral myocarditis.

**Fig. 14:**
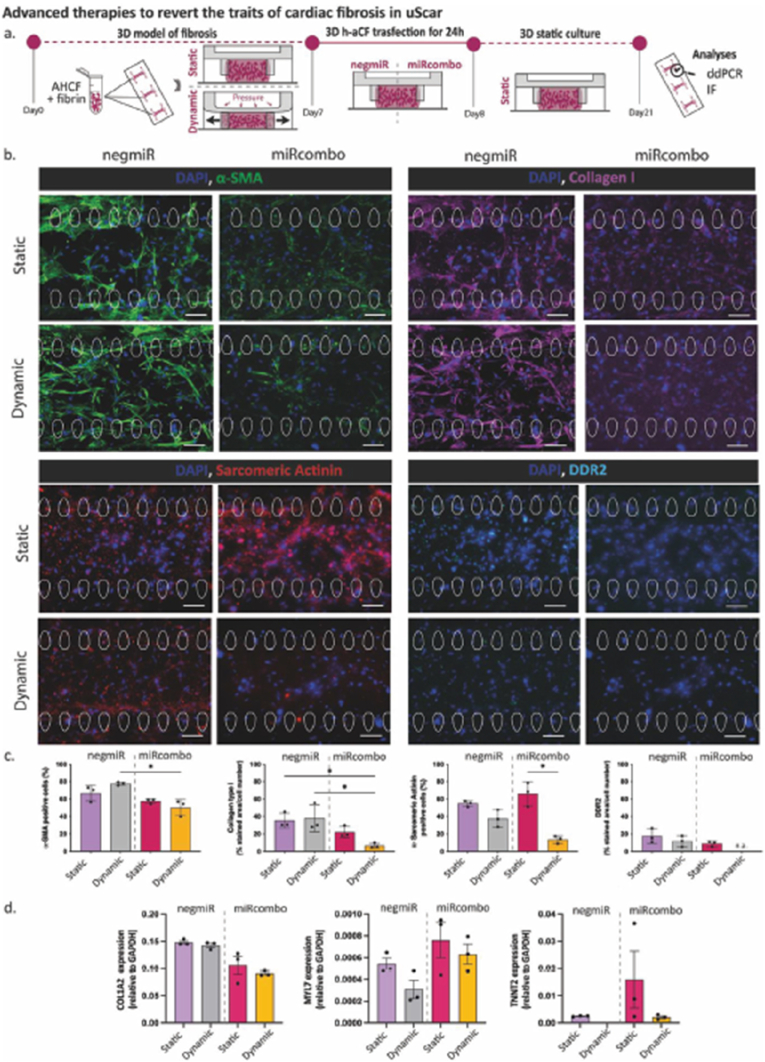
Effect of the advanced gene therapy in reverting the fibrotic traits elicited by the mechanical stimulation in the uScar human model: **A.** Schematic representation of the experimental plan; **B.** Representative immunofluorescence images of *α*-SMA (green), collagen I (purple), *α*-Sarcomeric Actinin (red), DDR2 (light blue) and DAPI counterstaining (blue) in the four experimental groups and **C-D.** relative quantifications. Scalebar 100 μm. Data are represented as mean ± SD. Kruskall-Wallis analyses of variance with Dunn's post-test were used (∗ = p < 0.05, ∗∗ = p < 0.01, ∗∗∗ = p < 0.001). Reprinted with permission from Ref. [205] © 2020 Adv. Mater. Techno published by Wiley-VCH GmbH. (For interpretation of the references to color in this figure legend, the reader is referred to the Web version of this article.)

In another study, Visone et al., uses a HoC device to evaluate the efficacy of a nanotherapeutic approach using a combination of four microRNAs (referred to as miRcombo, which includes miR-1, miR-133, miR-208, and miR-499) to mitigate cardiac diseases [205]. The miRNA was delivered using DE-DOPE lipoplexes, a novel method that enhances miRNA delivery and cytocompatibility compared to traditional transfection agents [215]. Initially, miRcombo was transfected into AHCFs cultured in 2D conditions for 24 h and then it was embedded in a 3D fibrin hydrogel within the mBeat Stretch Platform. After a week of static culture, the microtissues were subjected to dynamic conditions (cyclic mechanical strain) or maintained in static conditions. By analyzing the expression of CMs markers in both conditions, miRcombo increased the expression of cardiomyocyte markers (such as TNNT2 and MYL7) in static conditions. However, its efficacy decreased under dynamic conditions, where mechanical stimulation led to a downregulation of these markers and an upregulation of COL1A2, a fibrosis marker. Furthermore, when miRcombo was applied to preformed uScar models that had already undergone mechanical stimulation, it successfully reduced fibrotic traits, such as the number of α-SMA positive cells and collagen deposition, although it did not effectively induce a transition to a CMs-like phenotype. This study highlights the urgent need to considered the mechanical environment since the nanotherapeutic approach has potential in mitigating fibrosis, but the effectiveness was affected by it (Fig. 14) [205].

Li et al. work aimed to develop a 3D microfluidic device for evaluating the hepatotoxicity of NPs and concentrated on the effects of SPIONs (Fe3O4) with a diameter of 10 nm. To manufacture the microfluidic device, three layers had to be joined together: a PDMS cover, a silicon interlayer containing the cell culture chamber and a glass base. Primary Lewis rat hepatocytes were separated and cultured in the microfluidic device. Fibronectin (50 μg/mL) was applied to the glass of the device to encourage cell attachment. After being placed in the device, the cells were subjected to a regulated flow of culture media at 25, 50 and 100 μL/h. To assess the toxicity of SPIONs, short-term (3 days) and long-term (7 days) tests were carried out on the chip using concentrations of 50, 100 and 200 μg/mL. Liver functions, such as albumin (ALB) synthesis and urea production, were analyzed through ELISA and colorimetric assays, respectively, and cell viability was monitored by cell viability assays (Live/Dead) using fluorescent microscopy. Significant reductions in ALB and urea production, as well as in cell viability, were observed, indicating that SPIONs had more severe hepatotoxic effects under flow conditions. The cell damages were attributed to cumulative exposure under flow and the microfluidic model's characteristic of reducing NPs aggregation. In terms of maintaining hepatic function and sensitivity in toxicity detection, the microfluidic chip outperformed traditional static models. The study concluded that cumulative exposure to NPs under dynamic conditions intensified hepatic cell damage, highlighting the importance of flow in the design of toxicity assay systems. The authors demonstrated that treatment with APAP (40 mM) led to the complete loss of liver-specific functions, such as urea and ALB secretion, in the 3D hepatocyte chip within 48 h, corroborating previous studies [192].

In the study by Wu et al., a digital OoC was developed to evaluate hepatotoxicity, focusing on the research of EVs-based immunotherapies for the treatment of liver cancer. In the study, the fabrication of NPs was carried out through microfluidics and electrospray, aiming to create uniform microscopic spheres. Using this method, an electric voltage is applied to the alginate solution, creating a liquid jet that breaks up into droplets due to flow instability. When the droplets dry out as they get closer to a collector, NPs are produced. The effectiveness of NPs in therapeutic applications depends on their capacity to have their size modified by applying different voltages and flow rates. HepG2 hepatocytes (liver cancer), HFF-1, and HUVEC were among the cells that were employed and encased in hydrogel microspheres. To construct autonomous, uniform units with a diameter of 200 μm, the microspheres were made using a microfluidic electrospray process with 2 % sodium alginate combined with a calcium chloride solution, which serves as a crosslinking agent. The NPs studied were EVs generated by NK-92MI cells, described as potential immunotherapeutic agents. Drugs such as Sorafenib were encapsulated within the NPs during the production process to ensure that the drug was integrated into the alginate matrix and allow for regulated drug release. The authors demonstrated that EVs derived from NK-92MI cells (NK EVs) maintained high cell viability in microspheres treated with concentrations of 10 and 30 μg/mL, while a concentration of 50 μg/mL significantly reduced viability to 87.6 % highlighting the immunotherapeutic potential of these vesicles against liver cancer. Thus, the digital OoC more closely resembles the *in vivo* environment, enabling quick and precise reactions when evaluating the toxicity and effectiveness of drugs [206].

LUoC models offer a sophisticated platform for assessing NPs toxicity and their potential to damage lung epithelial cells and lung barrier integrity, with their effectiveness being influenced by factors such as the composition, surface charge, and size of NPs, directly affect their interaction with cells and tissues. This is particularly important given the growing concern over the health and environmental effects of NPs, especially regarding their inhalation into the lungs [199,210]. Zhang et al. used a 3D LUoC microfluidic technology to create an *in vitro* model representative of the human alveolar-capillary barrier. The main objective was to evaluate the pulmonary toxicity of NPs, particularly zinc oxide (ZnO) and titanium dioxide (TiO_2_), which are commonly present in industrial products and the environment. ZnO NPs, which were prepared from aqueous solutions of zinc oxide with a final concentration of 200 mg/mL, were used with TiO_2_ NPs suspended in ultrapure water at the same concentration. To define the microstructural pattern, a silicon wafer was coated with an 80 μm layer of SU8-3500 photoresist, heated, and exposed to UV radiation. The 3D structure was subsequently created by applying a second 220 μm layer. To replicate the alveolar microenvironment and promote cell attachment, a layer of Matrigel composed of type I collagen and chitosan was applied to the chip channels to mimic the ECM. HPAEpiCs and HUVEC were cultured in the channels of the device, forming a functional alveolar-capillary barrier. The NPs were introduced into the chip after the formation of the alveolar-capillary barrier, injected at varying concentrations (50–200 μg/mL) to simulate acute pulmonary exposure. Barrier integrity was evaluated by measuring the expression of junction proteins (E-cadherin and VE-cadherin), ROS production, macromolecular permeability, and apoptosis induction. The results showed that TiO_2_ NPs caused minimal damage to the alveolar-capillary barrier, maintaining the integrity of cell junction expression. The apoptotic effects of TiO_2_ and ZnO NPs on HPAEpiCs and HUVECs were demonstrated, showing that TiO_2_ NPs induced minimal apoptosis in both cell types, while ZnO NPs caused a dose-dependent increase in apoptosis, particularly at concentrations of 100 and 200 μg/mL. A marked increase in permeability was observed, with higher doses leading to faster diffusion of dextran across the barrier [199].

**Fig. 15:**
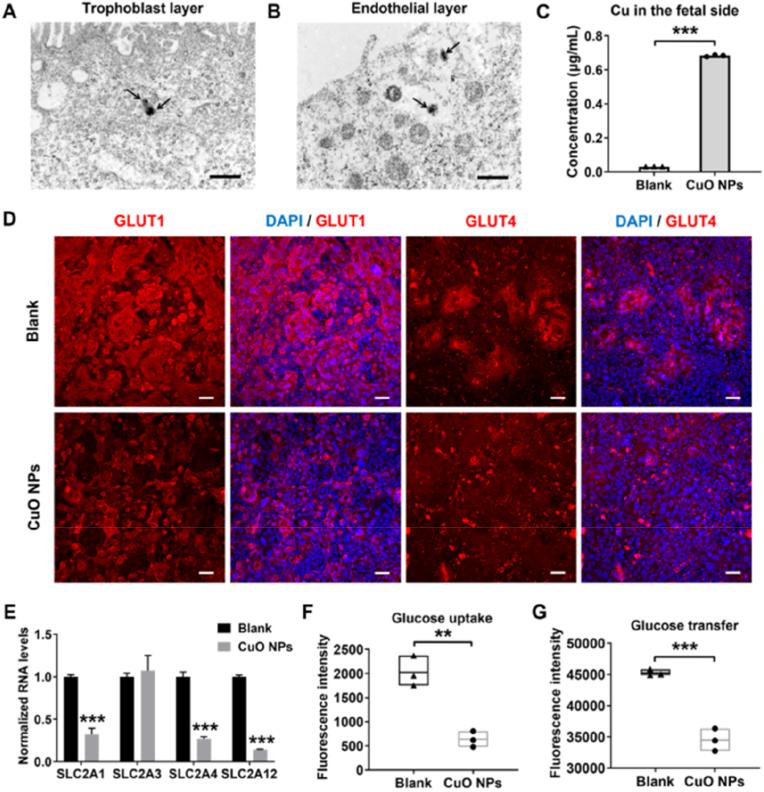
Effects of CuO NPs exposure on the placental barrier function. **(A**–**B)** Transmission electron microscopic images illustrate the distribution of CuO NPs in the trophoblastic epithelium and endothelial layer. Scale bars represent 0.5 μm. **(C)** Concentrations of Cu in the fetal side of placental models exposed to CuO NPs or without exposure were detected by ICP-MS. **(D)** Representative fluorescent images of trophoblast cells stained with DAPI (blue), anti-GLUT1 and anti-GLUT4 (red) after exposure to CuO NPs or without exposure. Scale bars represent 50 μm. **(E)** Normalized mRNA levels of glucose transporter genes SLC2A1, SLC2A3, SLC2A4, and SLC2A12 in trophoblast cells exposed to CuO NPs or without exposure. **(F)** Transport of glucose from maternal to fetal side across the trophoblastic barrier exposed to CuO NPs or without exposure was assessed using the 2-NBDG assay. **(G)** Glucose uptake by trophoblast cells exposed to CuO NPs or without exposure was measured using the 2-NBDG assay. The data are presented as the mean value; n = 3. Statistical significance was assessed with the unpaired two-tailed Student's t-test; ∗∗P < 0.002. Reprinted with permission from Ref. [203] © 2024 Published by Elsevier Inc. (For interpretation of the references to color in this figure legend, the reader is referred to the Web version of this article.)

Placenta-on-chip models are important tools in evaluating NPs toxicity. The models can help in understanding how NPs affect placental barrier integrity, induce inflammatory responses, and potentially contribute to fetal development issues. Confirming it, Cao et al. using a placenta-on-a-chip mode investigated the effects of CuO NPs on human trophoblast stem cells **(**hTSCs) [203]. The model was developed in a membrane in the middle layer of the device, the membrane was coated with collagen IV for 12 h hTSCs were then seeded on the insert membrane and maintained in culture medium for 48 h before HUVEC were added to the sublayer of the culture insert. The microdevice was placed on a rocker to create continuous fluid flow, which facilitated the differentiation of hTSCs into trophoblastic layers. As result of the exposure to CuO NPs, the hTSCs reduce hormone secretion and impaired glucose transport 1 (and glucose uptake), inhibiting the differentiation of hTSCs into syncytiotrophoblasts (Fig. 15). Additionally, it was observed an increased in ROS production and inflammatory responses provoke by CuO NPs disrupted autophagic flux and induced apoptosis in hTSCs. There is a potential risk of toxicity in maternal exposure to NPs during pregnancy, emphasizing the need for advanced models to evaluate nanotoxicity and its implications for fetal development [203].

### 3.4 ADME

**Fig. 16:**
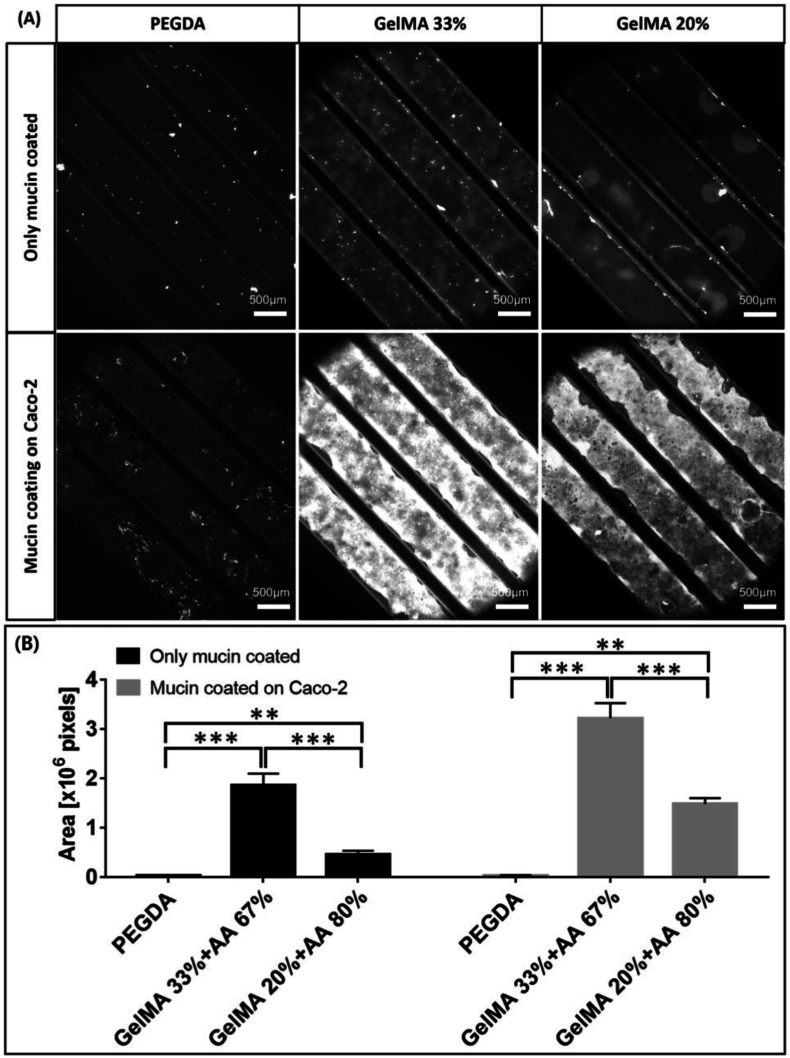
Intestinal mucosal adhesion of PEGDA and GelMA + AA particles. After injecting fluorescence-loaded particles into the GoC- mucus chip and allowing flow for 90 min, the remaining particles were observed. A photomicrograph of the particles remaining in the chip **(A)**, and a graph of quantifying the particles remaining in the chip **(B)** (∗*p* < 0.05, ∗∗*p* < 0.01, ∗∗∗*p* < 0.005). Reprinted with permission from Ref. [179] © 2023 The Korean BioChip Society 2023, BioChip Journal.

Lee et al. created a GoC-mucus chip model to study intestinal drug absorption and the adhesion of mucoadhesive polymer particles. The microfluidic device contains two-layer channels that mimic intestinal movement, divided by a porous polyester membrane. The PDMS Sylgard 184 A and B pre-polymer solution and silicone oil were homogenized using a homogenizer (T10 basic Ultra-Turrax, IKA®) to ensure that the components were evenly dispersed. Then, the mixture was exposed to 1000 mW/cm^2^ of UV light for 5 min to activate the photoinitiator and generate free radicals to promote the polymerization of the pre-polymer and the formation of NPs. The mixture was transferred to a conical tube, and the NPs and silicone oil were separated by centrifugation. To incorporate the fluorescent substance (Rhodamine B), the loading procedure was performed, in which the NPs were submerged for 24 h in a 10 mg/mL Rhodamine B solution. During polymerization, FITC-dextran was introduced so that the fluorescent ingredient could be directly integrated into the polymer matrix. Size control is essential for the NPs to stick to the intestinal mucosa and distribute drugs efficiently, and for this reason, the NPs were characterized to confirm that their diameters ranged from 10 to 20 μm. Caco-2 cells were grown in PDMS microchannels, and purified mucin was added to simulate the intestinal mucus layer. The results showed that, compared to PEGDA particles, GelMA + AA particles demonstrated better adhesion to mucus and greater pH sensitivity, indicating their higher applicability as oral drug delivery systems. The fact that the absorption efficiency was proportional to the adhesion of the particles validated the viability of the developed system as a model to evaluate drug absorption and particle adhesion in the colon. Furthermore, it was concluded that the intestinal mucosal adhesion of PEGDA and GelMA + AA particles can improve the therapeutic effect by increasing the contact time between the intestinal mucosa and the drug carrier (Fig. 16). Based on these results, the GelMA 33 % + AA 67 % particles were chosen as agents for oral drug delivery in subsequent experiments [179].

Meghani et al. developed a biomimetic model called the alveolus-epithelium-on-a-chip (AEOC) to evaluate human albumin (HSA) NPs encapsulating ZnO quantum dots. The ZnO NPs had a diameter of approximately 60 nm, and their synthesis was carried out by mixing HSA with a ZnO solution under controlled pH and temperature conditions. The ZnO core was encapsulated in a matrix formed by the HSA molecules surrounding the ZnO particles. High encapsulation efficiency, as measured by the weight of ZnO in the NPs divided by the entire amount of ZnO initially introduced, was guaranteed by adjusting the ratio of HSA to ZnO. To replicate the malignant alveolar epithelium, fibroblasts and A549 lung cancer cells were mixed into a type I collagen ECM. The model also featured integrated sensors for TEER and pH, enabling real-time monitoring of cellular conditions and the impact of the NPs. The study results demonstrated that HSA-ZnO NPs had an encapsulation efficiency of 69.51 %, and controlled release tests showed that ZnO quantum dots exhibited a 90 % release in acidic pH, reflecting the tumor environment. The AEOC model validated cell viability before treatment and showed a significant dose-dependent reduction in cell viability after exposure to NPs at doses of 10 and 50 μg/mL. Fluorescence and confocal assays indicated that cancer cells effectively absorbed the NPs [197].

Multi-OoC systems provide a robust platform for evaluating the efficacy and toxicity of NPs in a systemic way. For example, Esch et al. *created* a microfluidic body-on-chip system to explore the effects of 50 nm carboxylated PS NPs on human tissues [183]. This system integrates *in vitro* models of the GI tract, represented by a co-culture of Caco-2 and HT29-MTX cells, and liver tissue, represented by HepG2/C3A cells, within a microfluidic device. The main findings indicate that while the GI tract effectively limits NPs absorption, allowing only about 9.5 % of the NPs to cross the epithelial barrier, the presence of GI tissue enhances liver injury at lower NPs concentrations than expected. The system was exposed to NPs at different concentrations. By using NPs at concentrations of 240 and 480 × 10^11^ NPs/mL there was an increase in AST levels in the medium collected from the liver chamber which is a biomarker for liver injury. The study emphasizes that the body-on-a-chip system is a valuable tool for assessing the interactions and potential systemic toxic effects of NPs on human tissues [183].

**Fig. 17:**
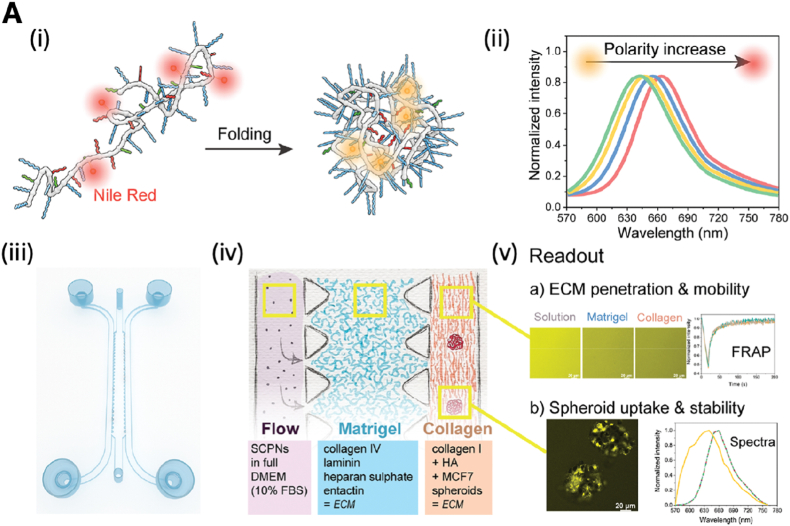
A CoC platform for analyzing NPs behavior and therapeutic potential. **A(i):** SCPNs containing Nile Red exhibited fluorescence in aqueous solutions, with the emission color varying based on the polarity of the surrounding environment. A more hydrophobic internal structure, resulting from SCPNs collapse, causes a blue shift in fluorescence, as shown in panel **A(ii)**. **A(iii):** to study SCPNs within a 3D tumor-like environment, the DAX1 microfluidic chip from AIM Biotech was used. This device comprised three parallel channels separated by triangular pillar arrays; **A(iv):** the central channel was filled with a Matrigel-based ECM (ECM). A collagen type I and hyaluronic acid mixture containing MCF7 tumor spheroids was introduced into the right channel to mimic a TME. SCPNs suspended in DMEM medium were added to the left channel, allowing diffusion across channels over 24 h; **A(v):** key assessments include SCPNs penetration into the ECM, uptake by tumor spheroids, and particle stability in various chip locations. Fluorescence recovery after photobleaching (FRAP) was used to evaluate SCPNs diffusion. Nile Red's emission intensity and spectral shift provided insights into SCPNs uptake and stability within MCF7 spheroids. Reprinted with permission from Ref. [180] © 2024 Small Methods published by Wiley-VCH GmbH. (For interpretation of the references to color in this figure legend, the reader is referred to the Web version of this article.)

Nanoscale drug delivery systems are extensively studied to enhance cancer treatment outcomes [[216], [217], [218], [219], [220], [221]]. The most significant challenge is predicting how NPs will behave *in vivo* due to the complex nature of tumors and their surrounding microenvironment. Indeed, the TME plays a critical role in influencing NPs delivery to cancer cells by regulating fluid and solute transport, NPs permeability, and sequestration by off-target cells [222,223]. This aspect was recently explored by *Deng* et al. They used a microfluidic CoC platform that recreated the TME to study the diffusion, cellular uptake, and stability of single-chain polymeric NPs (SCPNs). The commercial DAX-1 chip from AIM Biotech was chosen for the study. This chip presented three channels separated by triangular pillars. The central channel was filled with an ECM made of Matrigel. The right channel, representing the TME, was loaded with a mixture of collagen type I and hyaluronic acid supplemented with breast cancer cell (MCF7) spheroids. In the left channel, a solution of SCPNs was introduced and allowed to diffuse over 24 h (Fig. 17Ai-v). Using this 3D system, the authors examined how variations in the polymer's microstructure influenced SCPNs movement through the ECM and their internalization by cancer cells. They created a library of SCPNs with diverse microstructures, all of which demonstrated effective ECM penetration. However, their cellular uptake and stability varied with structural differences. For instance, glucose-based NPs achieved the highest uptake in spheroids, followed by charged NPs. Charged NPs showed an open conformation, while those stabilized by internal hydrogen bonds retained a compact structure within tumor spheroids. Overall, the study demonstrated that the 3D microfluidic CoC platform is a powerful tool for analyzing how polymer microstructure impacts SCPNs stability, offering valuable insights for the rational design of NPs for targeted therapeutic applications [180].

### 3.5 Theranostics

GoC model facilitates the understanding of oral drug absorption, metabolism, and the interactions of NPs with the intestinal barrier. The complex environment of the intestine, which includes a mucus layer, many cell types, and GoC bacteria, has a significant impact on the permeability and efficacy of oral drugs [179,194]. NPs are increasingly being used in drug delivery systems due to their ability to increase drug stability and bioavailability. Nonetheless, significant concerns have been expressed about their potential toxicity, particularly because of the effects on intestinal health, including disruption of the intestinal barrier and microbiota [196]. GoC platforms offer a more reliable and effective system for studying these effects, providing an *in vitro* environment that mimics the physiological conditions of the intestine accurately than traditional models [195]. Lee et al. investigated the use of Pt DENs to measure oxygen gradients in a GoC model. Caco-2 epithelial cells were used in the study to form a polarized monolayer to simulate the intestinal barrier under flow and mechanical deformation conditions. To create dendrimers, an aqueous solution of G6-NH2 (10 μM, pH 5) was added to potassium tetrachloroplatinate (II) (K_2_PtCl_4_). During the 76-h stirring period, Pt^2+^ ions were able to coordinate with the tertiary amine groups of the dendrimer. After 48 h of dialysis against ultrapure water, impurities were removed using excess sodium borohydride (NaBH_4_) for subsequent chemical reduction. Based on the Pt^2+^/dendrimer ratio, the resulting NPs were named G6-NH2(Ptm), where "m" represents the number of platinum atoms encapsulated. When creating the Pt DENs, platinum ions (Pt^2+^) were encapsulated in sixth-generation PAMAM dendrimers with amine (G6-NH2) and hydroxyl (G6-OH) terminals, which acted as matrices. Several molar ratios of Pt^2+^/dendrimer (55, 200, 220, 550, 880, and 1320) were used to change the catalytic properties of the NPs. When compared to lower ratios, the catalytic efficiency of the 1320 ratio showed an improvement, which indicated low polydispersity and average diameters close to the calculated theoretical values, ranging from 1.9 ± 0.2 nm to 3.1 ± 0.3 nm. These findings confirm the robust size control and uniformity of the NPs in different formulations, and that they are viable tools for oxygen mapping in the field due to their versatile enzyme-mimetic activity and efficiency in detecting oxygen gradients in physiological microenvironments [194].

## 4 Clinical translation

**Fig. 18:**
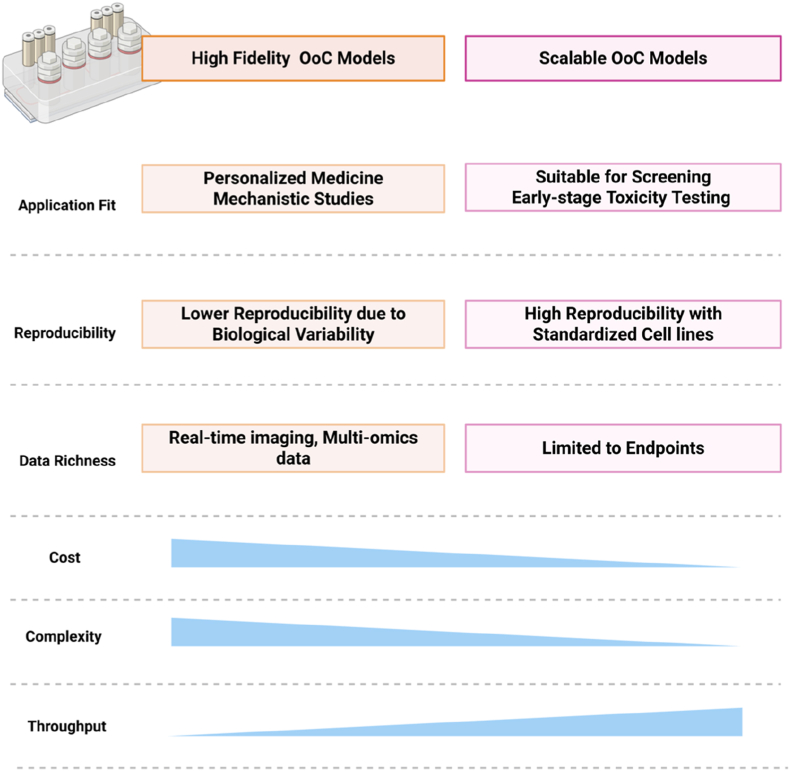
Comparison between High-Fidelity and Scalable OoC Models across Key Evaluation Criteria. This diagram contrasts the two approaches based on five critical factors: model complexity, cost and throughput, data richness, reproducibility, and application fit. Created by Biorender.

Developing NPs with the right size, shape, and surface properties is complex and requires precise control and screening to achieve the final formulation. The failure rate of NPs in animal tests is quite high [224]. While machine learning and AI have significantly improved the pre-screening process for NPs, the use of animals in testing remains high, and many NPs still fail at this stage [225]. Integrating OoC technology as an intermediate step between *in vitro* and *in vivo* testing can indeed help address these issues. These systems allow for detailed mechanistic studies of NPs interactions with human tissues, which are often challenging to perform in animal models. When selecting an OoC model for NPs studies, researchers must consider trade-offs between physiological fidelity and scalability. High physiological fidelity models offer complex, multi-cellular, and dynamic flow systems that closely mimic human organs, but they come with higher costs and lower throughput. On the other hand, scalable models are simpler, more cost-effective, and offer higher throughput, but they may lack the detailed physiological context needed for certain applications as illustrated in Fig. 18.

Nowadays OoC are an emerging technology for evaluating the safety and efficacy of NPs. To support clinical translation, efforts are underway to improve the accessibility, scalability, and commercialization of OoC platforms. These include standardizing fabrication, integrating user-friendly interfaces, and developing disease-specific models such as CoC and multi-organ systems. Recent studies have demonstrated how OoC can replicate complex human physiological interactions more accurately than traditional models such as animal models. For example, Li et al. constructed a perfusable 3D human microvessel network within a microfluidic device to mimic human blood vessels (HUVEC co-cultured with fibrin hydrogel) with the aim of evaluate vascular toxicity after exposure to inhaled ANPs [212]. Exposing the cells with ANPs led to increased vascular permeability and disruption of tight junctions, as shown by a non-uniform expression of ZO-1. The ANPs induce oxidative stress, which correlates with inflammation and endothelial dysfunction. However, the addition of antioxidants like vitamin C helped restore ZO-1 levels and reduce permeability. Additionally, the study highlights the potential to replace animal testing, as comparisons between the OoC system and animal experiments revealed similar findings to those observed in the 3D microvessel model. Male BALB/c mice were used to evaluate the effects of ANPs, with doses administered via intratracheal instillation. The results from these animal studies indicated that ANP exposure led to oxidative stress and disrupted the expression of various biomarkers. Specifically, both models demonstrated increased levels of ROS, interleukin-1 (IL-1), and nuclear factor kappa B (NF-κB) following ANPs exposure, suggesting a consistent pathological response across both experimental setups [212]. In another study, Kammala et al. developed a system to address the drug transport and metabolism during gestation by create a multi-organ feto-maternal interface on-chip (FMi-PLA-OoC) that simulate the complex interactions within the placenta and fetal membranes [226]. The differences between the PKs of pravastatin in the FMi-PLA-OoC model and traditional animal models were analyzed. In the FMi-PLA-OoC, pravastatin transfer rates were approximately 18 % within 8 h, which aligns closely with human clinical data and offers a more accurate representation of drug behavior during pregnancy. In contrast, animal models demonstrated a supraphysiologic drug accumulation in the amniotic fluid, reaching around 33 %. This discrepancy suggests that animal models may not effectively mimic human placental transfer dynamics, as they often exhibit higher drug concentrations than what is observed in human studies [226].

These studies showed the potential for OoC models to replace animal testing in early-phase safety and efficacy evaluations. With a particular focus on the recent FDA initiative to phase out mandatory animal testing for monoclonal antibodies and other drugs. As announced in April 2025, the FDA now encourages the use of New Approach Methodologies (NAMs)—including.

OoC systems, organoids, and AI-based models—for investigational new drug (IND) applications. This marks a significant regulatory shift that directly supports the clinical relevance and adoption of OoC technologies.

Each OoC model such as vascular, liver, kidney, or multi-organ offers distinct advantages and limitations depending on the specific application, such as biodistribution, toxicity, or pharmacokinetics. Key parameters such as shear stress ranges, cellular composition, barrier integrity, and detection modalities (e.g., fluorescence, electrochemical sensing) significantly influence the interpretation of NP behavior. Moreover, trade-offs between physiological fidelity and scalability must be considered. To guide researchers in selecting the most appropriate platform, we present a comparative matrix summarizing the strengths, weaknesses, and technical specifications of commonly used OoC systems for NP studies (see Table 3).

**Table 3:** OoC overview for NPs applications.

OoC Model	Strengths	Weaknesses	Key Parameters	Functions	Refs
**Vascular-on-Chip**	Mimics endothelial barrier and shear stress Real-time flow control	Limited metabolic activity Lacks organ-specific context	Shear stress: 0.5–20 dyn/cm^2^ Endothelial cells (HUVEC, iPSC-derived) Fluorescence, TEM, ICP-MS	Nanoparticle circulation Endothelial permeability	[227]
**Liver-on-Chip**	Metabolic competence CYP450 activity Bile canaliculi formation	Complex to maintain Limited flow dynamics	Shear stress: 0.1–1 dyn/cm^2^ Primary hepatocytes or HepaRG LC-MS/MS, ELISA, confocal microscopy	Metabolism Hepatotoxicity Clearance studies	[228]
**Lung-on-Chip**	Air-liquid interface Cyclic stretch Alveolar-capillary interface	Difficult to scale Sensitive to flow and pressure	Shear stress: 0.2–2 dyn/cm^2^ AECs + endothelial cells Fluorescence, SEM, cytokine assays	Inhaled nanoparticles Pulmonary toxicity	[229]
**Kidney-on-Chip**	Filtration and reabsorption modeling Proximal tubule simulation	Fragile structures Limited long-term viability	Shear stress: 0.2–1 dyn/cm^2^ RPTECs or podocytes TEER, albumin uptake, LC-MS	Nephrotoxicity Renal clearance	[74]
**Gut-on-Chip**	Microbiome integration Mucus layer Peristalsis simulation	High variability Complex co-culture systems	Shear stress: 0.02–0.08 dyn/cm^2^ Caco-2, HT29-MTX Permeability assays, qPCR, microscopy	Oral delivery Absorption and barrier studies	[230]
**Cancer-on-Chip**	Mimics tumor microenvironment Allows for drug penetration studies	Complex co-culture systems High variability	Shear stress: 0.1–1 dyn/cm^2^ Tumor cells + stromal cells Fluorescence, confocal microscopy, qPCR	Cancer drug testing Tumor growth and metastasis studies	[231]
**Heart-on-Chip**	Simulates cardiac tissue contraction Real-time monitoring of beating	Sensitive to mechanical stress Limited long-term viability	Shear stress: 0.5–2 dyn/cm^2^ Cardiomyocytes (iPSC-derived) Impedance, calcium imaging, MEA	Cardiotoxicity Drug-induced arrhythmia studies	[232]
**Brain-on-Chip**	Models blood-brain barrier Neuronal network activity	High complexity Difficult to maintain	Shear stress: 0.1–0.5 dyn/cm^2^ Neurons + astrocytes Electrophysiology, calcium imaging, TEER	Neurotoxicity Blood-brain barrier penetration	[233]
**Skin-on-Chip**	Mimics skin barrier and wound healing; Integrates multiple skin layers	Limited to 2D cultures; Lacks full immune system integration	Shear stress: N/A; Keratinocytes, fibroblasts; TEER, imaging	Dermatological testing; Wound healing studies	[234]
**Joint-on-Chip**	Replicates joint microenvironment; Models cartilage and synovium	Complex to maintain; Limited long-term viability	Shear stress: N/A; Chondrocytes, synoviocytes; Mechanical testing, imaging	OA research; Drug testing for joint diseases	[235]
**Multi-Organ-on-Chip**	Simulates inter-organ interactions; Models systemic metabolism	Highly complex; Expensive and low throughput	Shear stress: Variable; Multiple cell types; Multi-omics, imaging	Systemic drug metabolism; Multi-organ toxicity studies	[236]
**Spleen-on-Chip**	Models immune cell filtration and activation; Mimics splenic architecture	Limited to specific immune functions; Complex to replicate	Shear stress: N/A; Splenocytes, endothelial cells; Flow cytometry, imaging	Immunological studies; Hematological research	[237]

Recent innovations in immune-competent OoC platforms have expanded their capabilities to capture key immune interactions, including cytokine signaling, immune cell trafficking, and tissue-specific responses. Researchers can assess the effectiveness of immunotherapies in a controlled, human-relevant environment by using integrated tumor-on-chip models, which have successfully replicated cytotoxic NK cell infiltration into 3D tumor microenvironments [238]. Similar to this, lymphoid OoC systems have made it possible to investigate B cell memory and antibody production in response to mRNA vaccines, providing new information on adaptive immunity *in vitro* [239]. Multi-organ OoC models, like a combined intestine-liver-immune system, have been used to study systemic inflammation in ulcerative colitis in addition to single-organ platforms. This shows how useful these systems are for simulating intricate disease pathophysiology and immune-organ crosstalk [240].

For applications in inflammatory disorders, cancer, and infectious diseases, the incorporation of innate and adaptive immune cells from primary tissues or iPSCs, brings OoC models closer to clinical relevance. Compared to traditional *in vitro* techniques, immune-competent systems have several advantages, such as the capacity to mimic tissue-specific inflammation and dynamic immune responses without the need of animal models, which frequently do not fully reflect human outcomes because of interspecies variability. But there are still challenges for reproducibility across labs, guaranteeing cellular maturity, and attaining long-term immune cell viability [159]. Future directions include the development of autologous models using patient-derived immune and parenchymal cells, as well as the integration of immune sensors and biosignal feedback to study immunotoxicity in real time [239].

## 5 Conclusions and future perspectives

OoC technologies are rapidly emerging as tools in preclinical research, offering superior physiological relevance compared to traditional *in vitro* systems and animal models. In the context of NPs toxicity assessment, OoC provide a more accurate representation of human-specific responses, particularly in modeling ADME processes, as well as immune interactions. Unlike conventional cell cultures, OoC recreate dynamic microenvironments and tissue-tissue interfaces, while avoiding the interspecies variability inherent to animal models. Recent advances in OoC technology have enabled the incorporation of both innate and adaptive immune components into microphysiological systems. These immune-competent models recapitulate key aspects of human immunophysiology, including cytokine signaling, immune cell trafficking, and tissue-specific inflammation. For instance, LUoC systems have been engineered to model bacterial pneumonia, while LoC platforms have been used to study radiation-induced hepatic injury—both integrating immune cell dynamics to enhance translational relevance [159].

Advances in OoC technology are leading to the development of multiorgan systems, which can model the interactions between different organs and provide a more comprehensive understanding of NPs behavior in the human body and systemic effects compared to single-organ studies. The development of multi-OoC systems that can replicate interactions between many tissues should be the goal of future research. While OoC models mimic specific organ environments, they may not fully capture secondary and systemic toxicities that can be observed in whole-animal studies. These models allow for detailed studies on the toxicity of NPs, helping to identify potential adverse effects early in the development process. This could lead to more reliable safety assessments and reduce the risk of adverse effects in humans.

A major frontier in OoC development is the integration of patient-derived cells to create personalized microphysiological systems. These platforms can capture individual variability in immune responses, metabolism, and genetic background, enabling personalized toxicity testing and therapeutic optimization. Personalized OoC could be particularly impactful in oncology, immunotherapy, and rare disease research, where patient-specific responses to nanomedicines are highly variable. As biobanking and stem cell technologies advance, the feasibility of generating patient-specific OoC models is rapidly increasing, paving the way for precision nanomedicine.

Despite these advantages, several translational challenges must be addressed to fully realize the clinical potential of OoC platforms. First, the lack of standardized materials, hydrogel formulations, and flow control systems limits reproducibility across laboratories and platforms. Variability in biomaterials can significantly affect NPs adsorption, bioavailability, and immune responses. Establishing regulatory-grade standards for device fabrication and biological inputs is essential for adoption. Second, scalability remains a challenge, as current fabrication methods—such as soft lithography and molding—are cost-intensive and design-restrictive. To transition OoC systems from lab-scale prototypes to industrial tools, scalable and modular manufacturing approaches must be developed. Enhancing the durability and operational lifespan of these systems is also critical for long-term studies and regulatory testing. Third, clinical validation through benchmarking against human data and regulatory alignment is critical for widespread adoption. The future of OoC technology lies in its integration with advanced analytical tools such as artificial intelligence (AI) and machine learning. The combination of OoC with AI can significantly enhance data analysis, enable high-throughput screening and optimize the prediction of NPs toxicity and efficacy. Machine learning models have already been employed to predict nano-bio interactions, showing strong correlation with experimental results from OoC. These technologies can enhance data analysis, identify patterns, and predict outcomes with greater precision.

At least, regulation and legislation are important to ensure all of those. Legislation such as FDA Modernization Act 2.0 has encouraged the paradigmatic shift from animal testing towards OoC technology paving the way for smoother integration of OoC technology into the current regulatory framework. This implementation is being made possible through a unified effort by the academic and industrial research alongside regulation authorities. Ultimately, the FDA led through a pioneering shift toward more human-relevant drug testing by replacing animal models in the development of monoclonal antibody therapies and other drugs. This new approach aims to enhance drug safety, speed up evaluations, reduce animal use, cut R&D costs, and potentially lower drug prices [241].

As highlighted in the review, combining OoC technology with NPs holds great promise for more accurate and reliable preclinical testing of engineered and accelerate NPs for therapeutic uses. Although the work is still in its early stages, it is not difficult to foresee that OoC technology will play an instrumental role in the development of nanotherapeutics in the future. As the field moves forward, the synergy between bioengineering, computational modeling, and regulatory science will be pivotal. By addressing current limitations and aligning efforts across sectors, OoC technology is well-positioned to become the next-generation preclinical testing—offering safer, faster, and more cost-effective pathways to clinical translation in nanomedicine.

## CRediT authorship contribution statement

**Ana Regina Sampaio:** Writing – original draft, Methodology, Investigation, Data curation. **Renata Faria Maia:** Writing – original draft, Investigation, Formal analysis, Data curation. **Maria Camilla Ciardulli:** Writing – review & editing, Investigation. **Hélder A. Santos:** Writing – review & editing, Validation, Supervision, Funding acquisition, Conceptualization. **Bruno Sarmento:** Writing – review & editing, Supervision, Project administration, Conceptualization.

## Declaration of competing interest

The authors declare that they have no known competing financial interests or personal relationships that could have appeared to influence the work reported in this paper.

## Data Availability

Data will be made available on request.

## References

[1] Pimenta J., Ribeiro R., Almeida R., Costa P.F., Da Silva M.A., Pereira B. (2022). Organ-on-Chip approaches for intestinal 3D *in vitro* modeling. Cell. Mol. Gastroenterol. Hepatol..

[2] Bein A., Shin W., Jalili-Firoozinezhad S., Park M.H., Sontheimer-Phelps A., Tovaglieri A., Chalkiadaki A., Kim H.J., Ingber D.E. (2018). Microfluidic Organ-on-a-Chip models of human intestine. Cell. Mol. Gastroenterol. Hepatol..

[3] Ahmed T. (2022). rgan-on-a-chip microengineering for bio-mimicking disease models and revolutionizing drug discovery. Biosens. Bioelectron. X.

[4] Gil J.F., Moura C.S., Silverio V., Gonçalves G., Santos H.A. (2023). Cancer models on chip: paving the way to large‐scale trial applications. Adv. Mater..

[5] Rimal R., Muduli S., Desai P., Marquez A.B., Möller M., Platzman I., Spatz J., Singh S. (2024). Vascularized 3D human skin models in the forefront of dermatological research. Adv. Healthcare Mater..

[6] Wang L., Chen Z., Xu Z., Yang Y., Wang Y., Zhu J., Guo X., Tang D., Gu Z. (2023). A new approach of using Organ-on-a-Chip and fluid–structure interaction modeling to investigate biomechanical characteristics in tissue-engineered blood vessels. Front. Physiol..

[7] Liu D., Zhang H., Fontana F., Hirvonen J.T., Santos H.A. (2018). Current developments and applications of microfluidic technology toward clinical translation of nanomedicines. Adv. Drug Deliv. Rev..

[8] Gimondi S., Ferreira H., Reis R.L., Neves N.M. (2023). Microfluidic devices: a tool for nanoparticle synthesis and performance evaluation. ACS Nano.

[9] Ingber D.E. (2022). Human organs-on-chips for disease modelling, drug development and personalized medicine. Nat. Rev. Genet..

[10] Singh G., Mishra A., Mathur A., Shastri S., Nizam A., Rizwan A., Dadial A.S., Firdous A., Hassan H. (2024). Advancement of organ-on-chip towards next generation medical technology. Biosens. Bioelectron. X.

[11] Stavrou M., Phung N., Grimm J., Andreou C. (2023). Organ-on-Chip systems as a model for nanomedicine. Nanoscale.

[12] Rodrigues R.O., Sousa P.C., Gaspar J., Bañobre‐López M., Lima R., Minas G. (2020). Organ‐on‐a‐Chip: a preclinical microfluidic platform for the progress of nanomedicine. Small.

[13] Chen X., Zhang Y.S., Zhang X., Liu C. (2021). Organ-on-a-Chip platforms for accelerating the evaluation of nanomedicine. Bioact. Mater..

[14] Sarmento B., Ribeiro A., Veiga F., Ferreira D., Neufeld R. (2007). Oral bioavailability of insulin contained in polysaccharide nanoparticles. Biomacromolecules.

[15] Fonte P., Reis S., Sarmento B. (2016). Facts and evidences on the lyophilization of polymeric nanoparticles for drug delivery. J. Contr. Release.

[16] Gunasekaran B.M., Srinivasan S., Ezhilan M., Rajagopal V., Nesakumar N. (2024). Advancements in organ-on-a-chip systems: materials, characterization, and applications. ChemistrySelect.

[17] Natarajan S., Harini K., Gajula G.P., Sarmento B., Neves-Petersen M.T., Thiagarajan V. (2019). Multifunctional magnetic iron oxide nanoparticles: diverse synthetic approaches, surface modifications, cytotoxicity towards biomedical and industrial applications. BMC Mater..

[18] Lin L., Yang C., Ding X., Zu Y., Wang W. (2024). Protective value of sevoflurane to glycocalyx under low shear stress based on a microfluidic vessel chip. ACS Mater. Lett..

[19] Huang Q., Yang T., Song Y., Sun W., Xu J., Cheng Y., Yin R., Zhu L., Zhang M., Ma L., Li H., Zhang H. (2024). A three-dimensional (3D) liver–kidney on a chip with a biomimicking circulating system for drug safety evaluation. Lab Chip.

[20] Kopp B., Khawam A., Di Perna K., Lenart D., Vinette M., Silva R., Zanoni T.B., Rore C., Guenigault G., Richardson E., Kostrzewski T., Boswell A., Van P., Valentine Iii C., Salk J., Hamel A. (2024). Liver-on-Chip model and application in predictive genotoxicity and mutagenicity of drugs. Mutat. Res. Genet. Toxicol. Environ. Mutagen.

[21] Zhang C.J., Meyer S.R., O'Meara M.J., Huang S., Capeling M.M., Ferrer-Torres D., Childs C.J., Spence J.R., Fontana R.J., Sexton J.Z. (2023). A human liver organoid screening platform for DILI risk prediction. J. Hepatol..

[22] Vivas A., Ijspeert C., Pan J.Y., Vermeul K., Van Den Berg A., Passier R., Keller S.S., Van Der Meer A.D. (2022). Generation and culture of cardiac microtissues in a microfluidic chip with a reversible open top enables electrical pacing, dynamic drug dosing and endothelial cell co‐culture. Adv. Mater. Technol..

[23] Cofiño-Fabres C., Boonen T., Rivera-Arbeláez J.M., Rijpkema M., Blauw L., Rensen P.C.N., Schwach V., Ribeiro M.C., Passier R. (2024). Micro-engineered heart tissues on-Chip with heterotypic cell composition display self-organization and improved cardiac function. Adv. Healthcare Mater..

[24] Wei W., Cardes F., Hierlemann A., Modena M.M. (2023). 3D in vitro blood‐brain‐barrier model for investigating barrier insults. Adv. Sci..

[25] De Rus Jacquet A., Alpaugh M., Denis H.L., Tancredi J.L., Boutin M., Decaestecker J., Beauparlant C., Herrmann L., Saint-Pierre M., Parent M., Droit A., Breton S., Cicchetti F. (2023). The contribution of inflammatory astrocytes to BBB impairments in a brain-chip model of parkinson's disease. Nat. Commun..

[26] Seo S., Jang M., Kim H., Sung J.H., Choi N., Lee K., Kim H.N. (2023). Neuro‐Glia‐Vascular‐on‐a‐Chip system to assess aggravated neurodegeneration via brain endothelial cells upon exposure to diesel exhaust particles. Adv. Funct. Mater..

[27] Orge I.D., Nogueira Pinto H., Silva M.A., Bidarra S.J., Ferreira S.A., Calejo I., Masereeuw R., Mihăilă S.M., Barrias C.C. (2024). V vascular units as advanced living materials for Bottom-Up engineering of perfusable 3D microvascular networks. Bioact. Mater..

[28] Nie J., Lou S., Pollet A.M.A.O., van Vegchel M., Bouten C.V.C., den Toonder J.M.J. (2024). A cell pre-wrapping seeding technique for hydrogel-based tubular Organ-On-A-Chip. Adv. Sci..

[29] Marder M., Remmert C., Perschel J.A., Otgonbayar M., Von Toerne C., Hauck S., Bushe J., Feuchtinger A., Sheikh B., Moussus M., Meier M. (2024). Stem cell-derived Eessels-on-Chip for cardiovascular disease modeling. Cell Rep..

[30] Baranowska P., Kopińska M., Kołodziejek D., Jastrzębska E., Brzózka Z. (2024). Microfluidic system for generating a three-dimensional (3D) vascularized Islet-on-a-Chip model. Sensor. Actuator. B Chem..

[31] Nahon D.M., Vila Cuenca M., Van Den Hil F.E., Hu M., De Korte T., Frimat J.-P., Van Den Maagdenberg A.M.J.M., Mummery C.L., Orlova V.V. (2024). Self-assembling 3D vessel-on-chip model with hiPSC-Derived astrocytes. Stem Cell Rep..

[32] Yrjänäinen A., Mesiä E., Lampela E., Kreutzer J., Vihinen J., Tornberg K., Vuorenpää H., Miettinen S., Kallio P., Mäki A.-J. (2024). Barrier-free, open-top microfluidic chip for generating two distinct, interconnected 3D microvascular networks. Sci. Rep..

[33] Li Y., Li Y., Chen H. (2024). The effect of ultrasound-assisted thrombolysis studied in Blood-on-a-Chip. Artif. Organs.

[34] Goh T., Gao L., Singh J., Totaro R., Carey R., Yang K., Cartwright B., Dennis M., Ju L.A., Waterhouse A. (2024). Platelet adhesion and activation in an ECMO Thrombosis-on-a-Chip model. Adv. Sci..

[35] Takahashi N., Yoshino D., Sugahara R., Hirose S., Sone K., Rieu J.-P., Funamoto K. (2023). Microfluidic platform for the reproduction of hypoxic vascular microenvironments. Sci. Rep..

[36] Yu Z., Chen Y., Li J., Chen C., Lu H., Chen S., Zhang T., Guo T., Zhu Y., Jin J., Yan S., Chen H. (2024). A tempo-spatial controllable microfluidic shear-stress generator for In-Vitro mimicking of the thrombus. J. Nanobiotechnol..

[37] Singh J., Gambhir T., Goh T., Van Vuuren I., Gao L., Wise S.G., Waterhouse A. (2024). Spatiotemporally mapped endothelial dysfunction at bifurcations in a coronary artery-on-a-chip. Adv. Mater. Technol..

[38] Paloschi V., Pauli J., Winski G., Wu Z., Li Z., Botti L., Meucci S., Conti P., Rogowitz F., Glukha N., Hummel N., Busch A., Chernogubova E., Jin H., Sachs N., Eckstein H.-H., Dueck A., Boon R.A., Bausch A.R., Maegdefessel L. (2024). Utilization of an Artery-on-a-Chip to unravel novel regulators and therapeutic targets in vascular diseases. Adv. Healthcare Mater..

[39] Bittenbinder M.A., Bonanini F., Kurek D., Vulto P., Kool J., Vonk F.J. (2024). Using Organ-on-a-Chip technology to study haemorrhagic activities of snake venoms on endothelial tubules. Sci. Rep..

[40] Harada K., Wenlong W., Shinozawa T. (2024). Physiological platelet aggregation assay to mitigate drug-induced thrombocytopenia using a microphysiological system. Sci. Rep..

[41] Wu Q., Xue R., Zhao Y., Ramsay K., Wang E.Y., Savoji H., Veres T., Cartmell S.H., Radisic M. (2024). Automated fabrication of a scalable Heart-on-a-Chip device by 3D printing of thermoplastic elastomer nanocomposite and hot embossing. Bioact. Mater..

[42] Bannerman D., Pascual-Gil S., Wu Q., Fernandes I., Zhao Y., Wagner K.T., Okhovatian S., Landau S., Rafatian N., Bodenstein D.F., Wang Y., Nash T.R., Vunjak-Novakovic G., Keller G., Epelman S., Radisic M. (2024). Heart-on-a-Chip model of epicardial–myocardial interaction in ischemia reperfusion injury. Adv. Healthcare Mater..

[43] Contato A., Gagliano O., Magnussen M., Giomo M., Elvassore N. (2022). Timely delivery of cardiac mmRNAs in microfluidics enhances cardiogenic programming of human pluripotent stem cells. Front. Bioeng. Biotechnol..

[44] Arslan U., Brescia M., Meraviglia V., Nahon D.M., Van Helden R.W.J., Stein J.M., Van Den Hil F.E., Van Meer B.J., Vila Cuenca M., Mummery C.L., Orlova V.V. (2023). V vascularized hiPSC-Derived 3D cardiac microtissue on chip. Stem Cell Rep..

[45] Sun L., Wang Y., Bian F., Xu D., Zhao Y. (2023). Bioinspired optical and electrical dual-responsive Heart-on-a-Chip for hormone testing. Sci. Bull..

[46] Wu Q., Rafatian N., Wagner K.T., Blamer J., Smith J., Okhovatian S., Aggarwal P., Wang E.Y., Banerjee A., Zhao Y., Nash T.R., Lu R.X.Z., Portillo-Esquivel L.E., Li C.Y., Kuzmanov U., Mandla S., Virlee E., Landau S., Lai B.F., Gramolini A.O., Liu C., Fleischer S., Veres T., Vunjak-Novakovic G., Zhang B., Mossman K., Broeckel U., Radisic M. (2024). SARS-CoV-2 pathogenesis in an angiotensin II–Induced Heart-on-a-Chip disease model and extracellular vesicle screening. Proc. Natl. Acad. Sci..

[47] Mair D.B., Tsui J.H., Higashi T., Koenig P., Dong Z., Chen J.F., Meir J.U., Smith A.S.T., Lee P.H.U., Ahn E.H., Countryman S., Sniadecki N.J., Kim D.-H. (2024). Spaceflight-induced contractile and mitochondrial dysfunction in an automated Heart-on-a-chip platform. Proc. Natl. Acad. Sci..

[48] Ates B., Eroglu T., Sahsuvar S., Kirimli C.E., Kocaturk O., Senay S., Gok O. (2024). Hydrogel-integrated Heart-on-a-Chip platform for assessment of myocardial ischemia markers. ACS Omega.

[49] Shang Y., Sun L., Gan J., Xu D., Zhao Y., Sun L. (2024). A biomimetic cardiac Fibrosis-on-a-Chip as a visible disease model for evaluating mesenchymal stem cell-derived exosome therapy. ACS Nano.

[50] Wang P., Jin L., Zhang M., Wu Y., Duan Z., Guo Y., Wang C., Guo Y., Chen W., Liao Z., Wang Y., Lai R., Lee L.P., Qin J. (2023). Blood–brain barrier injury and neuroinflammation induced by SARS-CoV-2 in a lung–brain microphysiological system. Nat. Biomed. Eng..

[51] Yang T., Velagapudi R., Kong C., Ko U., Kumar V., Brown P., Franklin N.O., Zhang X., Caceres A.I., Min H., Filiano A.J., Rodriguiz R.M., Wetsel W.C., Varghese S., Terrando N. (2023). Protective effects of Omega-3 fatty acids in a blood–brain barrier-on-chip model and on postoperative delirium-like behaviour in mice. Br. J. Anaesth..

[52] Choi J., Choi H.K., Lee K. (2023). *In situ* detection of neuroinflammation using multicellular 3D neurovascular-unit-on-a-chip. Adv. Funct. Mater..

[53] Su S.-H., Song Y., Stephens A., Situ M., McCloskey M.C., McGrath J.L., Andjelkovic A.V., Singer B.H., Kurabayashi K. (2023). A tissue chip with integrated digital immunosensors: *in situ* brain endothelial barrier cytokine secretion monitoring. Biosens. Bioelectron..

[54] Ferrari E., Monti E., Cerutti C., Visone R., Occhetta P., Griffith L.G., Rasponi M. (2023). A method to generate perfusable physiologic-like vascular channels within a liver-on-chip model. Biomicrofluidics.

[55] Freag M.S., Namgung B., Reyna Fernandez M.E., Gherardi E., Sengupta S., Jang H.L. (2021). Human nonalcoholic steatohepatitis on a chip. Hepatol. Commun..

[56] Li S., Li C., Khan M.I., Liu J., Shi Z., Gao D., Qiu B., Ding W. (2023). Microneedle array facilitates hepatic sinusoid construction in a large-scale liver-acinus-chip microsystem. Microsyst. Nanoeng..

[57] Rajan S.A.P., Sherfey J., Ohri S., Nichols L., Smith J.T., Parekh P., Kadar E.P., Clark F., George B.T., Gregory L., Tess D., Gosset J.R., Liras J., Geishecker E., Obach R.S., Cirit M. (2023). A novel milli-fluidic liver tissue chip with continuous recirculation for predictive pharmacokinetics applications. AAPS J..

[58] Liu T., Ge Y., Chen Z., Wu L., Tian T., Yao W., Zhao J. (2023). Synergistic modulation of a tunable microenvironment to fabricate a liver fibrosis chip for drug testing. ACS Biomater. Sci. Eng..

[59] Morelli M., Cabezuelo Rodríguez M., Queiroz K. (2024). A high-throughput gut-on-chip platform to study the epithelial responses to enterotoxins. Sci. Rep..

[60] Carius P., Weinelt F.A., Cantow C., Holstein M., Teitelbaum A.M., Cui Y. (2024). Addressing the ADME challenges of compound loss in a PDMS-based gut-on-chip microphysiological system. Pharmaceutics.

[61] Van Nieuwenhuyse B., Merabishvili M., Goeders N., Vanneste K., Bogaerts B., De Jode M., Ravau J., Wagemans J., Belkhir L., Van Der Linden D. (2024). Phage-mediated digestive decolonization in a Gut-on-a-Chip model: a tale of gut-specific bacterial prosperity. Viruses.

[62] Gleeson J.P., Zhang S.Y., Subelzu N., Ling J., Nissley B., Ong W., Nofsinger R., Kesisoglou F. (2024). Head-to-Head comparison of Caco-2 transwell and Gut-on-a-Chip models for assessing oral peptide formulations. Mol. Pharm..

[63] Pöschl F., Höher T., Pirklbauer S., Wolinski H., Lienhart L., Ressler M., Riederer M. (2023). Dose and route dependent effects of the mycotoxin deoxynivalenol in a 3D Gut-on-a-Chip model with flow, toxicol. In Vitro.

[64] You M., Xu M. (2024). Ginseng oligopeptides improve the intestinal physiology and promote the antioxidant capacity of the Gut-on-a-Chip model. Nutrients.

[65] Pan X., Chen J., Han J., Zhang W., Su W., Xu Z., Li X., Song M., Song W., Xie X., Wang L. (2024). Critical suitability evaluation of Caco-2 cells for Gut-on-a-Chip. ACS Appl. Mater. Interfaces.

[66] Kim W., Lee Y., Kang D., Kwak T., Lee H.-R., Jung S. (2023). 3D inkjet-bioprinted Lung-on-a-Chip. ACS Biomater. Sci. Eng..

[67] Yang S., Zhang T., Ge Y., Cheng Y., Yin L., Pu Y., Chen Z., Liang G. (2023). Sentinel supervised Lung-on-a-Chip: a new environmental toxicology platform for nanoplastic-induced lung injury. J. Hazard. Mater..

[68] Richter C., Latta L., Harig D., Carius P., Stucki J.D., Hobi N., Hugi A., Schumacher P., Krebs T., Gamrekeli A., Stöckle F., Urbschat K., Montalvo G., Lautenschläger F., Loretz B., Hidalgo A., Schneider‐Daum N., Lehr C. (2024). A stretchable human lung-on-chip model of alveolar inflammation for evaluating anti‐inflammatory drug response. Bioeng. Transl. Med..

[69] Ektnitphong V., Dias B.R.S., Campos P.C., Shiloh M.U. (2024).

[70] Gijzen L., Bokkers M., Hanamsagar R., Olivier T., Burton T.P., Tool L.M., Rahman M.F., Lowman J., Savova V., Means T.K., Lanz H.L. (2025). An immunocompetent human Kidney-on-a-Chip model to study renal inflammation and immune-mediated injury. Biofabrication.

[71] Payasi A., Yadav M.K., Chaudhary S., Aggarwal A. (2024). Evaluating nephrotoxicity reduction in a novel polymyxin B formulation: insights from a 3D Kidney-on-a-Chip model. Antimicrob. Agents Chemother..

[72] You Y., Zhang C., Guo Z., Xu F., Sun D., Xia J., Chen S. (2024). Lung-on-a-Chip composed of styrene-butadiene-styrene nano-Fiber/Porous PDMS composite membranes with cyclic triaxial stimulation. Microfluid. Nanofluidics.

[73] Chatterjee E., Rodosthenous R.S., Kujala V., Gokulnath P., Spanos M., Lehmann H.I., De Oliveira G.P., Shi M., Miller-Fleming T.W., Li G., Ghiran I.C., Karalis K., Lindenfeld J., Mosley J.D., Lau E.S., Ho J.E., Sheng Q., Shah R., Das S. (2023). Circulating extracellular vesicles in human cardiorenal syndrome promote renal injury in a kidney-on-chip system. JCI Insight.

[74] Kim S., Lee J.-B., Kim D., Kim K., Sung G.Y. (2024). Fabrication of nephrotoxic model by Kidney-on-a-Chip implementing renal proximal tubular function *in vitro*. BioChip J.

[75] Wilken G.J., Marschner J.A., Romagnani P., Anders H.-J. (2020). Kidney-on-the-Chip approach using primary human tubular cells in a 3D Co-Culture system. Nephrol. Dial. Transplant..

[76] Lapin B., Gropplero G., Vandensteen J., Mazloum M., Bienaimé F., Descroix S., Coscoy S. (2025). Decoupling shear stress and pressure effects in the biomechanics of autosomal dominant polycystic kidney disease using a perfused kidney-on-chip. Acta Biomater..

[77] Salameh S., Tissot N., Cache K., Lima J., Suzuki I., Marinho P.A., Rielland M., Soeur J., Takeuchi S., Germain S., Breton L. (2021). A perfusable vascularized full-thickness skin model for potential topical and systemic applications. Biofabrication.

[78] Jones C.F.E., Di Cio S., Connelly J.T., Gautrot J.E. (2022). Design of an integrated microvascularized human Skin-on-a-Chip tissue equivalent model. Front. Bioeng. Biotechnol..

[79] Sriram G., Alberti M., Dancik Y., Wu B., Wu R., Feng Z., Ramasamy S., Bigliardi P.L., Bigliardi-Qi M., Wang Z. (2018). Full-thickness human skin-on-chip with enhanced epidermal morphogenesis and barrier function. Mater. Today.

[80] Michielon E., Boninsegna M., Waaijman T., Fassini D., Spiekstra S.W., Cramer J., Gaudriault P., Kodolányi J., De Gruijl T.D., Homs‐Corbera A., Gibbs S. (2024). Environmentally controlled microfluidic system enabling immune cell flow and activation in an endothelialised skin‐on‐chip. Adv. Healthcare Mater..

[81] Sun S., Jin L., Zheng Y., Zhu J. (2022). Modeling human HSV infection via a vascularized immune-competent skin-on-chip platform. Nat. Commun..

[82] Ahn J., Ohk K., Won J., Choi D.-H., Jung Y.H., Yang J.H., Jun Y., Kim J.-A., Chung S., Lee S.-H. (2023). Modeling of three-dimensional innervated epidermal like-layer in a microfluidic chip-based coculture system. Nat. Commun..

[83] Quan Q., Weng D., Li X., An Q., Yang Y., Yu B., Ma Y., Wang J. (2022). Analysis of drug efficacy for inflammatory skin on an organ-chip system. Front. Bioeng. Biotechnol..

[84] Haque M.R., Wessel C.R., Leary D.D., Wang C., Bhushan A., Bishehsari F. (2022). Patient-derived pancreatic Cancer-on-a-Chip recapitulates the tumor microenvironment, microsyst. Nanoengineering.

[85] Truong D.D., Kratz A., Park J.G., Barrientos E.S., Saini H., Nguyen T., Pockaj B., Mouneimne G., LaBaer J., Nikkhah M. (2019). A human organotypic microfluidic tumor model permits investigation of the interplay between patient-derived fibroblasts and breast cancer cells. Cancer Res..

[86] Surendran V., Safarulla S., Griffith C., Ali R., Madan A., Polacheck W., Chandrasekaran A. (2024). Magnetically integrated tumor–vascular interface system to mimic pro-angiogenic endothelial dysregulations for On-Chip drug testing. ACS Appl. Mater. Interfaces.

[87] Veith I., Nurmik M., Mencattini A., Damei I., Lansche C., Brosseau S., Gropplero G., Corgnac S., Filippi J., Poté N., Guenzi E., Chassac A., Mordant P., Tosello J., Sedlik C., Piaggio E., Girard N., Camonis J., Shirvani H., Mami-Chouaib F., Mechta-Grigoriou F., Descroix S., Martinelli E., Zalcman G., Parrini M.C. (2024). Assessing personalized responses to Anti-PD-1 treatment using patient-derived lung Tumor-on-Chip. Cell Rep. Med..

[88] Tan J., Zhu L., Shi J., Zhang J., Kuang J., Guo Q., Zhu X., Chen Y., Zhou C., Gao X. (2024). Evaluation of drug resistance for EGFR-TKIs in lung cancer via multicellular Lung-on-a-Chip. Eur. J. Pharmaceut. Sci..

[89] Bai J., Haase K., Roberts J.J., Hoffmann J., Nguyen H.T., Wan Z., Zhang S., Sarker B., Friedman N., Ristić-Lehmann Č., Kamm R.D. (2021). A novel 3D vascular assay for evaluating angiogenesis across porous membranes. Biomaterials.

[90] Nagaraju S., Truong D., Mouneimne G., Nikkhah M. (2018). Microfluidic tumor–vascular model to study breast cancer cell invasion and intravasation. Adv. Healthcare Mater..

[91] Lee M., Kim S., Lee S.Y., Son J.G., Park J., Park S., Yeun J., Lee T.G., Im S.G., Jeon J.S. (2024). Hydrophobic surface induced pro-metastatic cancer cells for *In Vitro* extravasation models. Bioact. Mater..

[92] Conceição F., Sousa D.M., Loessberg-Zahl J., Vollertsen A.R., Neto E., Søe K., Paredes J., Leferink A., Lamghari M. (2022). A Metastasis-on-a-Chip approach to explore the sympathetic modulation of breast cancer bone metastasis. Mater. Today Bio.

[93] Aung A., Kumar V., Theprungsirikul J., Davey S.K., Varghese S. (2020). An engineered Tumor-on-a-Chip device with breast cancer–immune cell interactions for assessing T-cell recruitment. Cancer Res..

[94] Boussommier-Calleja A., Atiyas Y., Haase K., Headley M., Lewis C., Kamm R.D. (2019). The effects of monocytes on tumor cell extravasation in a 3D vascularized microfluidic model. Biomaterials.

[95] Maulana T.I., Teufel C., Cipriano M., Roosz J., Lazarevski L., Van Den Hil F.E., Scheller L., Orlova V., Koch A., Hudecek M., Alb M., Loskill P. (2024). Breast cancer-on-chip for patient-specific efficacy and safety testing of CAR-T cells. Cell Stem Cell.

[96] Haase K., Offeddu G.S., Gillrie M.R., Kamm R.D. (2020). Endothelial regulation of drug transport in a 3D vascularized tumor model. Adv. Funct. Mater..

[97] Li G., Li H., Ndour P.A., Franco M., Li X., MacDonald I., Dao M., Buffet P.A., Karniadakis G.E. (2024). Red blood cell passage through deformable interendothelial slits in the spleen: insights into splenic filtration and hemodynamics. Comput. Biol. Med..

[98] Zhang Y., Qiang Y., Li H., Li G., Lu L., Dao M., Karniadakis G.E., Popel A.S., Zhao C. (2024). Signaling-biophysical modeling unravels mechanistic control of red blood cell phagocytosis by macrophages in sickle cell disease. PNAS Nexus.

[99] Qiang Y., Sissoko A., Liu Z.L., Dong T., Zheng F., Kong F., Higgins J.M., Karniadakis G.E., Buffet P.A., Suresh S., Dao M. (2023). Microfluidic study of retention and elimination of abnormal red blood cells by human spleen with implications for sickle cell disease. Proc. Natl. Acad. Sci..

[100] Cao R., Wang Y., Liu J., Rong L., Qin J. (2023). Self-assembled human placental model from trophoblast stem cells in a dynamic Organ-on-a-Chip system. Cell Prolif..

[101] Lermant A., Rabussier G., Lanz H.L., Davidson L., Porter I.M., Murdoch C.E. (2023). Development of a human iPSC-Derived placental barrier-on-chip model. iScience.

[102] Vidal M.S., Richardson L.S., Kumar Kammala A., Kim S., Lam P.Y., Cherukuri R., Thomas T.J., Bettayeb M., Han A., Rusyn I., Menon R. (2024). Endocrine-disrupting compounds and their impact on human placental function: evidence from placenta organ-on-chip studies. Lab Chip.

[103] Jeong S., Fuwad A., Yoon S., Jeon T.-J., Kim S.M. (2024). A microphysiological model to mimic the placental remodeling during early stage of pregnancy under hypoxia-induced trophoblast invasion. Biomimetics.

[104] Lee E., Chan S.-L., Lee Y., Polacheck W.J., Kwak S., Wen A., Nguyen D.-H.T., Kutys M.L., Alimperti S., Kolarzyk A.M., Kwak T.J., Eyckmans J., Bielenberg D.R., Chen H., Chen C.S. (2023). A 3D biomimetic model of lymphatics reveals cell–cell junction tightening and lymphedema via a cytokine-induced ROCK2/JAM-A complex. Proc. Natl. Acad. Sci..

[105] Serrano J.C., Gillrie M.R., Li R., Ishamuddin S.H., Moeendarbary E., Kamm R.D. (2024). Microfluidic-based reconstitution of functional lymphatic microvasculature: elucidating the role of lymphatics in health and disease. Adv. Sci..

[106] Kwee B.J., Mansouri M., Akue A., Sung K.E. (2025). On-chip human lymph node stromal network for evaluating dendritic cell and T-Cell trafficking. Biofabrication.

[107] Zha D., Rayamajhi S., Sipes J., Russo A., Pathak H.B., Li K., Sardiu M.E., Bantis L.E., Mitra A., Puri R.V., Trinidad C.V., Cain B.P., Isenberg B.C., Coppeta J., MacLaughlan S., Godwin A.K., Burdette J.E. (2023). Proteomic profiling of fallopian tube-derived extracellular vesicles using a microfluidic tissue-on-chip system. Bioengineering.

[108] Ferreira D.A., Conde J.P., Rothbauer M., Ertl P., Granja P.L., Oliveira C. (2023). Bioinspired human Stomach-on-a-Chip with *in vivo* like function and architecture. Lab Chip.

[109] Schlünder K., Cipriano M., Zbinden A., Fuchs S., Mayr T., Schenke-Layland K., Loskill P. (2024). Microphysiological pancreas-on-chip platform with integrated sensors to model endocrine function and metabolism. Lab Chip.

[110] Zhang B., Korolj A., Lai B.F.L., Radisic M. (2018). Advances in Organ-on-a-Chip engineering. Nat. Rev. Mater..

[111] Middelkamp H.H.T., Weener H.J., Gensheimer T., Vermeul K., De Heus L.E., Albers H.J., Van Den Berg A., Van Der Meer A.D. (2024). Embedded macrophages induce intravascular coagulation in 3D blood vessel-on-chip. Biomed. Microdevices.

[112] Di Cio S., Marhuenda E., Haddrick M., Gautrot J.E. (2024). Vascularised cardiac Spheroids-on-a-Chip for testing the toxicity of therapeutics. Sci. Rep..

[113] Grisold W. (2024). The expanding burden of neurological disorders. Lancet Neurol..

[114] Wang Z., Zhang Y., Li Z., Wang H., Li N., Deng Y. (2023). Microfluidic Brain‐on‐a‐Chip: from key technology to system integration and application. Small.

[115] Li Z., Zhao Y., Lv X., Deng Y. (2023). Integrated brain on a chip and automated organ‐On‐chips systems. Interdiscip. Med..

[116] Wu D., Chen Q., Chen X., Han F., Chen Z., Wang Y. (2023). The blood–brain barrier: structure, regulation and drug delivery. Signal Transduct. Targeted Ther..

[117] Guarino V., Zizzari A., Bianco M., Gigli G., Moroni L., Arima V. (2023). Advancements in modelling human blood brain-barrier on a chip. Biofabrication.

[118] Bi W., Cai S., Lei T., Wang L. (2023). Implementation of blood-brain barrier on microfluidic chip: recent advance and future prospects. Ageing Res. Rev..

[119] Cecen B., Saygili E., Zare I., Nejati O., Khorsandi D., Zarepour A., Alarcin E., Zarrabi A., Topkaya S.N., Yesil-Celiktas O., Mostafavi E., Bal-Öztürk A. (2023). Biosensor integrated Brain-on-a-Chip platforms: progress and prospects in clinical translation. Biosens. Bioelectron..

[120] Marino A., Battaglini M., Lefevre M.C., Ceccarelli M.C., Ziaja K., Ciofani G. (2023). Sensorization of microfluidic Brain-on-a-Chip devices: towards a new generation of integrated drug screening systems. TrAC, Trends Anal. Chem..

[121] Tian T., Ho Y., Chen C., Sun H., Hui J., Yang P., Ge Y., Liu T., Yang J., Mao H. (2022). A 3D bio-printed spheroids based perfusion *in vitro* liver on chip for dug toxicity assays. Chin. Chem. Lett..

[122] Messelmani T., Le Goff A., Souguir Z., Maes V., Roudaut M., Vandenhaute E., Maubon N., Legallais C., Leclerc E., Jellali R. (2022). Development of liver-on-chip integrating a hydroscaffold mimicking the liver's extracellular matrix. Bioengineering.

[123] Ehrlich A., Duche D., Ouedraogo G., Nahmias Y. (2019). Challenges and opportunities in the design of liver-on-chip microdevices. Annu. Rev. Biomed. Eng..

[124] Liu H., Yin G., Kohlhepp M.S., Schumacher F., Hundertmark J., Hassan M.I.A., Heymann F., Puengel T., Kleuser B., Mosig A.S., Tacke F., Guillot A. (2024). Dissecting acute drug‐induced hepatotoxicity and therapeutic responses of steatotic liver disease using primary mouse liver and blood cells in a liver-on-a-chip model. Adv. Sci..

[125] Nedelcu A., Mosteanu O., Pop T., Mocan T., Mocan L. (2021). Recent advances in nanoparticle-mediated treatment of inflammatory bowel diseases. Appl. Sci..

[126] Mainali B.B., Yoo J.J., Ladd M.R. (2024). Tissue engineering and regenerative medicine approaches in colorectal surgery. Ann. Coloproctol..

[127] Antunes J.C., Seabra C.L., Domingues J.M., Teixeira M.O., Nunes C., Costa-Lima S.A., Homem N.C., Reis S., Amorim M.T.P., Felgueiras H.P. (2021). Drug targeting of inflammatory bowel diseases by biomolecules. Nanomaterials.

[128] Guo Y., Chen X., Gong P., Li G., Yao W., Yang W. (2023). The Gut–organ-axis concept: advances the application of gut-on-chip technology. Int. J. Mol. Sci..

[129] Rahman S., Ghiboub M., Donkers J.M., Van De Steeg E., Van Tol E.A.F., Hakvoort T.B.M., De Jonge W.J. (2021). The progress of intestinal epithelial models from cell lines to gut-on-chip. Int. J. Mol. Sci..

[130] Xian C., Zhang J., Zhao S., Li X.-G. (2023). Gut-on-a-Chip for disease models. J. Tissue Eng..

[131] Macedo M.H., Torras N., García-Díaz M., Barrias C., Sarmento B., Martínez E. (2023). The shape of our gut: dissecting its impact on drug absorption in a 3D bioprinted intestinal model. Biomater. Adv..

[132] Ashammakhi N., Nasiri R., Barros N.R.D., Tebon P., Thakor J., Goudie M., Shamloo A., Martin M.G., Khademhosseini A. (2020). Gut-on-a-chip: current progress and future opportunities. Biomaterials.

[133] Verhulsel M., Simon A., Bernheim-Dennery M., Gannavarapu V.R., Gérémie L., Ferraro D., Krndija D., Talini L., Viovy J.-L., Vignjevic D.M., Descroix S. (2021). Developing an advanced gut on chip model enabling the study of epithelial cell/fibroblast interactions. Lab Chip.

[134] Okamoto R., Mizutani T., Shimizu H. (2023). Development and application of regenerative medicine in inflammatory bowel disease. Digestion.

[135] Tan J., Sun X., Zhang J., Li H., Kuang J., Xu L., Gao X., Zhou C. (2022). Exploratory evaluation of EGFR-targeted anti-tumor drugs for lung cancer based on Lung-on-a-Chip. Biosensors.

[136] Tan J., Guo Q., Tian L., Pei Z., Li D., Wu M., Zhang J., Gao X. (2023). Biomimetic Lung-on-a-Chip to model virus infection and drug evaluation. Eur. J. Pharmaceut. Sci..

[137] Zhu L., Zhang J., Guo Q., Kuang J., Li D., Wu M., Mo Y., Zhang T., Gao X., Tan J. (2023). Advanced lung organoids and Lung-on-a-Chip for cancer research and drug evaluation: a review. Front. Bioeng. Biotechnol..

[138] Gu S., Xiao W., Yu Z., Xiao J., Sun M., Zhang L., Pan P., Xie L. (2025). Single-cell RNA-seq reveals the immune response of Co-Infection with Streptococcus pneumoniae after influenza A virus by a Lung-on-Chip: the molecular structure and mechanism of tight junction protein ZO-1. Int. J. Biol. Macromol..

[139] Kanabekova P., Dauletkanov B., Bekezhankyzy Z., Toktarkan S., Martin A., Pham T.T., Kostas K., Kulsharova G. (2024). A hybrid fluorescent nanofiber membrane integrated with microfluidic chips towards Lung-on-a-Chip applications. Lab Chip.

[140] Dasgupta Q., Jiang A., Wen A.M., Mannix R.J., Man Y., Hall S., Javorsky E., Ingber D.E. (2023). A human lung Alveolus-on-a-Chip model of acute radiation-induced lung injury. Nat. Commun..

[141] Francis I., Shrestha J., Paudel K.R., Hansbro P.M., Warkiani M.E., Saha S.C. (2022). Recent advances in Lung-on-a-Chip models. Drug Discov. Today.

[142] Lin K.-C., Yen C.-Z., Yang J.-W., Chung J.H.Y., Chen G.-Y. (2022). Airborne toxicological assessment: the potential of Lung-on-a-Chip as an alternative to animal testing. Mater. Today Adv..

[143] Ismayilzada N., Tarar C., Dabbagh S.R., Tokyay B.K., Dilmani S.A., Sokullu E., Abaci H.E., Tasoglu S. (2024). Skin-on-a-Chip technologies towards clinical translation and commercialization. Biofabrication.

[144] Monteduro A.G., Rizzato S., Caragnano G., Trapani A., Giannelli G., Maruccio G. (2023). Organs-on-Chips technologies – a guide from disease models to opportunities for drug development. Biosens. Bioelectron..

[145] Mori N., Morimoto Y., Takeuchi S. (2018). Perfusable and stretchable 3D culture system for skin-equivalent. Biofabrication.

[146] Ahn M., Cho W.-W., Park W., Lee J.-S., Choi M.-J., Gao Q., Gao G., Cho D.-W., Kim B.S. (2023). 3D biofabrication of diseased human skin models *in vitro*. Biomater. Res..

[147] Zhang X., Su R., Wang H., Wu R., Fan Y., Bin Z., Gao C., Wang C. (2024). The promise of synovial Joint-on-a-Chip in rheumatoid arthritis. Front. Immunol..

[148] Mirazi H., Wood S.T. (2025). Microfluidic chip-based Co-Culture system for modeling human joint inflammation in osteoarthritis research. Front. Pharmacol..

[149] Du C., Liu J., Liu S., Xiao P., Chen Z., Chen H., Huang W., Lei Y. (2024). Bone and joint‐on‐chip platforms: construction strategies and applications. Small Methods.

[150] Heidenberger J., Reihs E.I., Strauss J., Frauenlob M., Gültekin S., Gerner I., Toegel S., Ertl P., Windhager R., Jenner F., Rothbauer M. (2025). The effect of cyclic fluid perfusion on the proinflammatory tissue environment in osteoarthritis using equine Joint-on-a-Chip models. Lab Chip.

[151] Mainardi A., Börsch A., Occhetta P., Ivanek R., Ehrbar M., Krattiger L., Oertle P., Loparic M., Martin I., Rasponi M., Barbero A. (2025). An organ-on-chip platform for strain‐controlled, tissue‐specific compression of cartilage and mineralized osteochondral interface to study mechanical overloading in osteoarthritis. Adv. Healthcare Mater..

[152] Zhao J., Shen F., Sheng S., Wang J., Wang M., Wang F., Jiang Y., Jing Y., Xu K., Su J. (2025). Cartilage-on-Chip for osteoarthritis drug screening. Organoid Res..

[153] Petta D., D'Arrigo D., Salehi S., Talò G., Bonetti L., Vanoni M., Deabate L., De Nardo L., Dubini G., Candrian C., Moretti M., Lopa S., Arrigoni C. (2024). A personalized osteoarthritic Joint-on-a-Chip as a screening platform for biological treatments. Mater. Today Bio.

[154] Giannitelli S.M., Peluzzi V., Raniolo S., Roscilli G., Trombetta M., Mozetic P., Rainer A. (2024). On-Chip recapitulation of the tumor microenvironment: a decade of progress. Biomaterials.

[155] Li W., Zhou Z., Zhou X., Khoo B.L., Gunawan R., Chin Y.R., Zhang L., Yi C., Guan X., Yang M. (2023). 3D biomimetic models to reconstitute tumor microenvironment *in vitro*: spheroids, organoids, and tumor-on-a-chip. Adv. Healthcare Mater..

[156] Li C., Holman J.B., Shi Z., Qiu B., Ding W. (2023). On-Chip modeling of tumor evolution: advances, challenges and opportunities. Mater. Today Bio.

[157] De Visser K.E., Joyce J.A. (2023). The evolving tumor microenvironment: from cancer initiation to metastatic outgrowth. Cancer Cell.

[158] Hassell B.A., Goyal G., Lee E., Sontheimer-Phelps A., Levy O., Chen C.S., Ingber D.E. (2017). Human organ chip models recapitulate orthotopic lung cancer growth, therapeutic responses, and tumor dormancy *in vitro*. Cell Rep..

[159] Morrison A.I., Sjoerds M.J., Vonk L.A., Gibbs S., Koning J.J. (2024). *In vitro* immunity: an overview of immunocompetent organ-on-chip models. Front. Immunol..

[160] Li Z., Li D., Guo Y., Wang Y., Su W. (2021). Evaluation of hepatic drug-metabolism for glioblastoma using liver-brain chip. Biotechnol. Lett..

[161] Lee J., Mehrotra S., Zare-Eelanjegh E., Rodrigues R.O., Akbarinejad A., Ge D., Amato L., Kiaee K., Fang Y., Rosenkranz A., Keung W., Mandal B.B., Li R.A., Zhang T., Lee H., Dokmeci M.R., Zhang Y.S., Khademhosseini A., Shin S.R. (2021). A heart-breast Cancer-on-a-Chip platform for disease modeling and monitoring of cardiotoxicity induced by cancer chemotherapy. Small.

[162] Yu Y., Sun B., Ye X., Wang Y., Zhao M., Song J., Geng X., Marx U., Li B., Zhou X. (2024). Hepatotoxic assessment in a microphysiological system: simulation of the drug absorption and toxic process after an overdosed acetaminophen on intestinal-liver-on-chip. Food Chem. Toxicol..

[163] Brasino D.S.K., Speese S.D., Schilling K., Schutt C.E., Barton M.C., Linkable A. (2024). Polycarbonate gut microbiome-distal tumor chip platform for interrogating cancer promoting mechanisms. Adv. Sci..

[164] Fanizza F., Perottoni S., Boeri L., Donnaloja F., Negro F., Pugli F., Forloni G., Giordano C., Albani D. (2025). A gut–brain axis on-a-Chip platform for drug testing challenged with donepezil. Lab Chip.

[165] Xu H., Wen J., Yang J., Zhou S., Li Y., Xu K., Li W., Li S. (2024). Tumor-Microenvironment-on-a-Chip: the construction and application. Cell Commun. Signal..

[166] Hwangbo H., Chae S., Kim W., Jo S., Kim G.H. (2024). Tumor-on-a-Chip models combined with mini-tissues or organoids for engineering tumor tissues. Theranostics.

[167] Liu X., Fang J., Huang S., Wu X., Xie X., Wang J., Liu F., Zhang M., Peng Z., Hu N. (2021). Tumor-on-a-Chip: from bioinspired design to biomedical application. Microsyst. Nanoeng..

[168] McCrary M.W., Bousalis D., Mobini S., Song Y.H., Schmidt C.E. (2020). Decellularized tissues as platforms for *in vitro* modeling of healthy and diseased tissues. Acta Biomater..

[169] Fowler M., Moreno Lozano A., Krause J., Bednarz P., Pandey S., Ghayour M., Zhang Q., Veiseh O. (2025). Guiding vascular infiltration through architected GelMA/PEGDA hydrogels: an *in vivo* study of channel diameter, length, and complexity. Biomater. Sci..

[170] Wu Z., Liu R., Shao N., Zhao Y. (2025). Developing 3D bioprinting for organs-on-chips. Lab Chip.

[171] Dornhof J., Kieninger J., Rupitsch S.J., Weltin A. (2025). Microsensor systems for cell metabolism – from 2D culture to organ-on-chip (2019–2024). Lab Chip.

[172] Wang Y., Yung P., Lu G., Liu Y., Ding C., Mao C., Li Z.A., Tuan R.S. (2025). Musculoskeletal Organs‐on‐Chips: an emerging platform for studying the nanotechnology–biology interface. Adv. Mater..

[173] Novelli G., Spitalieri P., Murdocca M., Centanini E., Sangiuolo F. (2023). Organoid factory: the recent role of the human induced pluripotent stem cells (hiPSCs) in precision medicine. Front. Cell Dev. Biol..

[174] Huang Y., Liu T., Huang Q., Wang Y. (2024). From Organ-on-a-Chip to Human-on-a-Chip: a review of research progress and latest applications. ACS Sens..

[175] Viegas J., Sarmento B. (2024). Bridging the gap between testing and clinics exploring alternative pre-clinical models in melanoma research. Adv. Drug Deliv. Rev..

[176] Barbato M.G., Pereira R.C., Mollica H., Palange A., Ferreira M., Decuzzi P. (2021). A permeable on-Chip microvasculature for assessing the transport of macromolecules and polymeric nanoconstructs. J. Colloid Interface Sci..

[177] Dibble M., Di Cio’ S., Luo P., Balkwill F., Gautrot J.E. (2023). The impact of pericytes on the stability of microvascular networks in response to nanoparticles. Sci. Rep..

[178] Bazban-Shotorbani S., Gavins F., Kant K., Dufva M., Kamaly N. (2022). A biomicrofluidic screening platform for dysfunctional endothelium‐targeted nanoparticles and therapeutics. Adv. NanoBiomed Res..

[179] Lee S.Y., Lee Y., Choi N., Kim H.N., Kim B., Sung J.H. (2023). Development of gut-mucus chip for intestinal absorption study. BioChip J.

[180] Deng L., Olea A.R., Ortiz‐Perez A., Sun B., Wang J., Pujals S., Palmans A.R.A., Albertazzi L. (2024). Imaging diffusion and stability of single‐chain polymeric nanoparticles in a multi‐gel tumor-on-a-chip microfluidic device. Small Methods.

[181] Lu R., Lee B.J., Lee E. (2024). Three-dimensional Lymphatics-on-a-Chip reveals distinct, size-dependent nanoparticle transport mechanisms in lymphatic drug delivery. ACS Biomater. Sci. Eng..

[182] Wright L., Wignall A., Jõemetsa S., Joyce P., Prestidge C.A. (2023). A membrane-free microfluidic approach to mucus permeation for efficient differentiation of mucoadhesive and mucopermeating nanoparticulate systems. Drug Deliv. Transl. Res..

[183] Esch M.B., Mahler G.J., Stokol T., Shuler M.L. (2014). Body-on-a-Chip simulation with gastrointestinal tract and liver tissues suggests that ingested nanoparticles have the potential to cause liver injury. Lab Chip.

[184] Sharifi F., Yesil-Celiktas O., Kazan A., Maharjan S., Saghazadeh S., Firoozbakhsh K., Firoozabadi B., Zhang Y.S. (2020). A hepatocellular carcinoma–bone Metastasis-on-a-Chip model for studying thymoquinone-loaded anticancer nanoparticles. Bio-Des. Manuf..

[185] Li Z., Lin Z., Liu S., Yagi H., Zhang X., Yocum L., Romero‐Lopez M., Rhee C., Makarcyzk M.J., Yu I., Li E.N., Fritch M.R., Gao Q., Goh K.B., O'Donnell B., Hao T., Alexander P.G., Mahadik B., Fisher J.P., Goodman S.B., Bunnell B.A., Tuan R.S., Lin H. (2022). Human mesenchymal stem cell‐derived miniature joint system for disease modeling and drug testing. Adv. Sci..

[186] Oliveira I.M., Carvalho M.R., Fernandes D.C., Abreu C.M., Maia F.R., Pereira H., Caballero D., Kundu S.C., Reis R.L., Oliveira J.M. (2021). Modulation of inflammation by Anti-TNF α mAb-Dendrimer nanoparticles loaded in tyramine-modified gellan gum hydrogels in a Cartilage-on-a-Chip model. J. Mater. Chem. B.

[187] Neto E., Monteiro A.C., Leite Pereira C., Simões M., Conde J.P., Chu V., Sarmento B., Lamghari M. (2022). Micropathological chip modeling the neurovascular unit response to inflammatory bone condition. Adv. Healthcare Mater..

[188] Fayazbakhsh F., Hataminia F., Eslam H.M., Ajoudanian M., Kharrazi S., Sharifi K., Ghanbari H. (2023). Evaluating the antioxidant potential of resveratrol-gold nanoparticles in preventing oxidative stress in endothelium on a chip. Sci. Rep..

[189] Lu R.X.Z., Lai B.F.L., Benge T., Wang E.Y., Davenport Huyer L., Rafatian N., Radisic M. (2021). Heart-on-a-Chip platform for assessing toxicity of air pollution related nanoparticles. Adv. Mater. Technol..

[190] Palma-Florez S., López-Canosa A., Moralez-Zavala F., Castaño O., Kogan M.J., Samitier J., Lagunas A., Mir M. (2023). BBB-on-a-Chip with integrated Micro-TEER for permeability evaluation of multi-functionalized gold nanorods against alzheimer's disease. J. Nanobiotechnol..

[191] Liang J., Qi H., Zhu F., Chen S., Liu B., Sun C., Wang Y. (2024). *In situ* monitor l-Dopa permeability by integrating electrochemical sensor on the blood-brain barrier chip. Sensor. Actuator. B Chem..

[192] Li L., Gokduman K., Gokaltun A., Yarmush M.L., Usta O.B. (2019). A microfluidic 3D hepatocyte chip for hepatotoxicity testing of nanoparticles. Nanomedicine.

[193] Jiang T., Guo H., Xia Y.-N., Liu Y., Chen D., Pang G., Feng Y., Yu H., Wu Y., Zhang S., Wang Y., Wang Y., Wen H., Zhang L.W. (2021). Hepatotoxicity of copper sulfide nanoparticles towards hepatocyte spheroids using a novel multi-concave agarose chip method. Nanomedicine.

[194] Lee H., Shin W., Kim H.J., Kim J. (2021). Turn-On fluorescence sensing of oxygen with dendrimer-encapsulated platinum nanoparticles as tunable oxidase mimics for spatially resolved measurement of oxygen gradient in a human Gut-on-a-Chip. Anal. Chem..

[195] Zhu D., Zheng F., Chen Q.-L., Yang X.-R., Christie P., Ke X., Zhu Y.-G. (2018). Exposure of a soil collembolan to Ag nanoparticles and AgNO_3_ disturbs its associated microbiota and lowers the incidence of antibiotic resistance genes in the gut. Environ. Sci. Technol..

[196] Gokulan K., Williams K., Orr S., Khare S. (2020). Human intestinal tissue explant exposure to silver nanoparticles reveals sex dependent alterations in inflammatory responses and epithelial cell permeability. Int. J. Mol. Sci..

[197] Meghani N., Kim K.H., Kim S.H., Lee S.H., Choi K.H. (2020). Evaluation and live monitoring of pH-Responsive HSA-ZnO nanoparticles using a Lung-on-a-Chip model. Arch Pharm. Res. (Seoul).

[198] A A., J X., A V., M P.V. (2022). L-Cysteine capped zinc oxide nanoparticles induced cellular response on adenocarcinomic human alveolar basal epithelial cells using a conventional and Organ-on-a-Chip approach. Colloids Surf. B Biointerfaces.

[199] Zhang M., Xu C., Jiang L., Qin J. (2018). A 3D human Lung-on-a-Chip model for nanotoxicity testing. Toxicol. Res..

[200] Frantellizzi V., Verrina V., Raso C., Pontico M., Petronella F., Bertana V., Ballesio A., Marasso S.L., Miglietta S., Rosa P., Scibetta S., Petrozza V., De Feo M.S., De Vincentis G., Calogero A., Pani R., Perotto G., De Sio L. (2022). 99mTc-Labeled keratin gold-nanoparticles in a nephron-like microfluidic chip for photo-thermal therapy applications. Mater. Today Adv..

[201] Ji C., Zhang J., Shi L., Shi H., Xu W., Jin J., Qian H. (2024). Engineered extracellular vesicle-encapsulated chip as novel nanotherapeutics for treatment of renal fibrosis. Npj Regen. Med..

[202] Fernandez-Carro E., Salomon-Cambero R., Armero L., Castro-Abril H.A., Ayensa-Jiménez J., Martínez M.A., Ochoa I., Alcaine C., García I., Ciriza J. (2023). Nanoparticles stokes radius assessment through permeability coefficient determination within a new stratified epithelium on-Chip model. Artif. Cells, Nanomed. Biotechnol..

[203] Cao R., Guo Y., Liu J., Guo Y., Li X., Xie F., Wang Y., Qin J. (2024). Assessment of nanotoxicity in a human Placenta-on-a-Chip from trophoblast stem cells. Ecotoxicol. Environ. Saf..

[204] Yin F., Zhu Y., Zhang M., Yu H., Chen W., Qin J. (2019). A 3D human Placenta-on-a-Chip model to probe nanoparticle exposure at the placental barrier, toxicol. In Vitro.

[205] Visone R., Paoletti C., Cordiale A., Nicoletti L., Divieto C., Rasponi M., Chiono V., Occhetta P. (2024). *In vitro* mechanical stimulation to reproduce the pathological hallmarks of human cardiac fibrosis on a beating chip and predict the efficacy of drugs and advanced therapies. Adv. Healthcare Mater..

[206] Wu G., Wu J., Li Z., Shi S., Wu D., Wang X., Xu H., Liu H., Huang Y., Wang R., Shen J., Dong Z., Wang S. (2022). Development of digital Organ-on-a-Chip to assess hepatotoxicity and extracellular vesicle-based anti-liver cancer immunotherapy, bio-des. Manuf.

[207] Fieni C., Ciummo S.L., Sorrentino C., Marchetti S., Vespa S., Lanuti P., Lotti L.V., Di Carlo E. (2024). Prevention of prostate cancer metastasis by a CRISPR-delivering nanoplatform for Interleukin-30 genome editing. Mol. Ther..

[208] Abostait A., Tyrrell J., Abdelkarim M., Shojaei S., Tse W.H., El-Sherbiny I.M., Keijzer R., Labouta H.I. (2022). Placental nanoparticle Uptake-on-a-Chip: the impact of trophoblast syncytialization and shear stress. Mol. Pharm..

[209] Sakolish C.M., Philip B., Mahler G.J. (2019). A human proximal Tubule-on-a-Chip to study renal disease and toxicity. Biomicrofluidics.

[210] Amin Arefi S.M., Tony Yang C.W., Sin D.D., Feng J.J. (2020). Simulation of nanoparticle transport and adsorption in a microfluidic Lung-on-a-Chip device. Biomicrofluidics.

[211] Decsi B., Krammer R., Hegedűs K., Ender F., Gyarmati B., Szilágyi A., Tőtős R., Katona G., Paizs C., Balogh G.T., Poppe L., Balogh-Weiser D. (2019). Liver-on-a-Chip‒Magnetic nanoparticle bound synthetic metalloporphyrin-catalyzed biomimetic oxidation of a drug in a magnechip reactor. Micromachines.

[212] Li Y., Wu Y., Liu Y., Deng Q.-H., Mak M., Yang X. (2019). Atmospheric nanoparticles affect vascular function using a 3D human vascularized organotypic chip. Nanoscale.

[213] Rodrigues R.O., Shin S.-R., Bañobre-López M. (2024). Brain-on-a-Chip: an emerging platform for studying the nanotechnology-biology interface for neurodegenerative disorders. J. Nanobiotechnol..

[214] Costa S., Vilas-Boas V., Lebre F., Granjeiro J.M., Catarino C.M., Moreira Teixeira L., Loskill P., Alfaro-Moreno E., Ribeiro A.R. (2023). Microfluidic-based skin-on-chip systems for safety assessment of nanomaterials. Trends Biotechnol..

[215] Serpico L., Zhu Y., Maia R.F., Sumedha S., Shahbazi M.-A., Santos H.A. (2024). Lipid nanoparticles-based RNA therapies for breast cancer treatment. Drug Deliv. Transl. Res..

[216] Cheng R., Jiang L., Gao H., Liu Z., Mäkilä E., Wang S., Saiding Q., Xiang L., Tang X., Shi M., Liu J., Pang L., Salonen J., Hirvonen J., Zhang H., Cui W., Shen B., Santos H.A. (2022). A ph-responsive cluster metal–organic framework nanoparticle for enhanced tumor accumulation and antitumor effect. Adv. Mater..

[217] Almeida D.R.S., Gil J.F., Guillot A.J., Li J., Pinto R.J.B., Santos H.A., Gonçalves G. (2024). Advances in microfluidic‐based core@Shell nanoparticles fabrication for cancer applications. Adv. Healthcare Mater..

[218] Han H., Santos H.A. (2024). Nano‐ and micro‐platforms in therapeutic proteins delivery for cancer therapy: materials and strategies. Adv. Mater..

[219] Tramontano C., Martins J.P., De Stefano L., Kemell M., Correia A., Terracciano M., Borbone N., Rea I., Santos H.A. (2023). Microfluidic‐assisted production of gastro‐resistant active‐targeted diatomite nanoparticles for the local release of galunisertib in metastatic colorectal cancer cells. Adv. Healthcare Mater..

[220] Li J., Wang S., Fontana F., Tapeinos C., Shahbazi M.-A., Han H., Santos H.A. (2023). Nanoparticles-based phototherapy systems for cancer treatment: current status and clinical potential. Bioact. Mater..

[221] Känkänen V., Fernandes M., Liu Z., Seitsonen J., Hirvonen S.-P., Ruokolainen J., Pinto J.F., Hirvonen J., Balasubramanian V., Santos H.A. (2023). Microfluidic preparation and optimization of Sorafenib-Loaded poly(ethylene glycol-block-caprolactone) nanoparticles for cancer therapy applications. J. Colloid Interface Sci..

[222] Tapeinos C., Torrieri G., Wang S., Martins J.P., Santos H.A. (2023). Evaluation of cell membrane-derived nanoparticles as therapeutic carriers for pancreatic ductal adenocarcinoma using an *in vitro* tumour stroma model. J. Contr. Release.

[223] Azizi M., Jahanban-Esfahlan R., Samadian H., Hamidi M., Seidi K., Dolatshahi-Pirouz A., Yazdi A.A., Shavandi A., Laurent S., Be Omide Hagh M., Kasaiyan N., Santos H.A., Shahbazi M.-A. (2023). Multifunctional nanostructures: intelligent design to overcome biological barriers. Mater. Today Bio.

[224] Joyce P., Allen C.J., Alonso M.J., Ashford M., Bradbury M.S., Germain M., Kavallaris M., Langer R., Lammers T., Peracchia M.T., Popat A., Prestidge C.A., Rijcken C.J.F., Sarmento B., Schmid R.B., Schroeder A., Subramaniam S., Thorn C.R., Whitehead K.A., Zhao C.-X., Santos H.A. (2024). A translational framework to deliver nanomedicines to the clinic. Nat. Nanotechnol..

[225] Gao H., Li S., Lan Z., Pan D., Naidu G.S., Peer D., Ye C., Chen H., Ma M., Liu Z., Santos H.A. (2024). Comparative optimization of polysaccharide-based nanoformulations for cardiac RNAi therapy. Nat. Commun..

[226] Kammala A.K., Richardson L.S., Radnaa E., Han A., Menon R. (2023). Microfluidic technology and simulation models in studying pharmacokinetics during pregnancy. Front. Pharmacol..

[227] Ehlers H., Olivier T., Trietsch S.J., Vulto P., Burton T.P., Van Den Broek L.J. (2025). Microfluidic Artery-on-a-Chip model with unidirectional gravity-driven flow for high-throughput applications. Lab Chip.

[228] Liu J., Du Y., Xiao X., Tan D., He Y., Qin L. (2024). Construction of *in vitro* Liver-on-a-Chip models and application progress. Biomed. Eng. Online.

[229] Sheidaei Z., Akbarzadeh P., Kashaninejad N. (2025). Dynamics of nanoparticles in a 3D breathing Lung-on-a-Chip. Drug Deliv. Transl. Res..

[230] Ofori‐Kwafo A., Sigdel I., Al Mamun E., Zubcevic J., Tang Y. (2025). Gut‐on‐a‐Chip platforms: bridging *in vitro* and *in vivo* models for advanced gastrointestinal research. Phys. Rep..

[231] Nejati B., Shahhosseini R., Hajiabbasi M., Ardabili N.S., Baktash K.B., Alivirdiloo V., Moradi S., Rad M.F., Rahimi F., Farani M.R., Ghazi F., Mobed A., Alipourfard I. (2025). Cancer-on-Chip: a breakthrough Organ-on-a-Chip technology in cancer cell modeling. Med. Biol. Eng. Comput..

[232] Mozneb M., Jenkins A., Sances S., Pohlman S., Workman M.J., West D., Ondatje B., El-Ghazawi K., Woodbury A., Garcia V.J., Patel S., Arzt M., Dezem F., Laperle A.H., Moser V.A., Ho R., Yucer N., Plummer J., Barrett R.J., Svendsen C.N., Sharma A. (2024). Multi-lineage heart-chip models drug cardiotoxicity and enhances maturation of human stem cell-derived cardiovascular cells. Lab Chip.

[233] Deli M.A., Porkoláb G., Kincses A., Mészáros M., Szecskó A., Kocsis A.E., Vigh J.P., Valkai S., Veszelka S., Walter F.R., Dér A. (2024). Lab-on-a-Chip models of the blood–brain barrier: Evolution, problems, perspectives. Lab Chip.

[234] Sanches P.L., Vieira Carias R.B., Alves G.G., Catarino C.M., Bosquetti B., De Castilho Costa M.C., Di Pietro Micali A., Schuck D.C., Granjeiro J.M., Ribeiro A.R. (2025). Pre-validation of a novel reconstructed skin equivalent model for skin irritation and nanoparticle risk assessment. Nanoscale Adv..

[235] Roehm K.D., Chiesa I., Haithcock D., Gottardi R., Prabhakarpandian B. (2025). A vascularized microfluidic model of the osteochondral unit for modeling inflammatory response and therapeutic screening. Lab Chip.

[236] Rupar M.J., Hanson H., Rogers S., Botlick B., Trimmer S., Hickman J.J. (2024). Modelling the innate immune system in microphysiological systems. Lab Chip.

[237] Mohanan P.V. (2022). Microfluidics and Multi Organs on Chip.

[238] Marzagalli M., Pelizzoni G., Fedi A., Vitale C., Fontana F., Bruno S., Poggi A., Dondero A., Aiello M., Castriconi R., Bottino C., Scaglione S. (2022). A multi-organ-on-chip to recapitulate the infiltration and the cytotoxic activity of circulating NK cells in 3D matrix-based tumor model. Front. Bioeng. Biotechnol..

[239] Goekeri C., Linke K.A.K., Hoffmann K., Lopez-Rodriguez E., Gluhovic V., Voß A., Kunder S., Zappe A., Timm S., Nettesheim A., Schickinger S.M.K., Zobel C.M., Pagel K., Gruber A.D., Ochs M., Witzenrath M., Nouailles G. (2024). Enzymatic modulation of the pulmonary glycocalyx enhances susceptibility to *Streptococcus pneumoniae*. Am. J. Respir. Cell Mol. Biol..

[240] Janssen R., Benito‐Zarza L., Cleijpool P., Valverde M.G., Mihăilă S.M., Bastiaan‐Net S., Garssen J., Willemsen L.E.M., Masereeuw R. (2025). Biofabrication directions in recapitulating the immune system-on-a-chip. Adv. Healthcare Mater..

[241] (2025). FDA Announces Plan to Phase Out Animal Testing Requirement for Monoclonal Antibodies and Other Drugs.

